# Design and synthesis of new phosphazine and triazole derivatives for treatment of Alzheimer's disease: modulating ROS/JNK and Wnt/β-catenin signaling pathways

**DOI:** 10.1039/d5ra07584j

**Published:** 2026-01-14

**Authors:** Rania S. Salah, Naglaa F. El-Sayed, Marwa El-Hussieny, Shaimaa T. Mansour, Mohamed Othman, Marwa A. Fouad, Huda R. M. Rashdan, Ewies F. Ewies, Heba S. A. Gharib, Ghada H. Elsayed

**Affiliations:** a Hormones Department, Medical Research and Clinical Studies Institute, National Research Centre 33 El-Bohouth St., Dokki 12622 Giza Egypt ghadanrc@yahoo.com gh.hamdi@nrc.sci.eg; b Organometallic and Organometalloid Chemistry Department, National Research Centre 33ElBohouth St. (Former El Tahrir) Dokki 12622 Giza Egypt ewiesfawzy@yahoo.com ef.ewies@nrc.sci.eg; c Normandie Univ., UNILEHAVRE, FR 3038 CNRS, URCOM 76600 Le Havre France; d UR 3221, INC3M CNRS-FR 3038 UFR ST BP: 1123, 25 Rue Philipe Lebon 76063 Le Havre France; e Pharmaceutical Chemistry Department, Faculty of Pharmacy, Cairo University Kasr El-Aini St. Cairo 11562 Egypt; f Pharmaceutical Chemistry Department, School of Pharmacy, Newgiza University Newgiza, Km 22 Cairo-Alexandria Desert Road Cairo Egypt; g Chemistry of Natural and Microbial Products Department, Pharmaceutical and Drug Industries Research Institute, National Research Centre 33 ElBohouth St. (Former El Tahrir) Dokki 12622 Giza Egypt; h Behaviour and Management of Animal, Poultry and Aquatics Department, Faculty of Veterinary Medicine, Zagazig University 44511 Zagazig Egypt; i Stem Cells Lab, Centre of Excellence for Advanced Sciences, National Research Centre 33 El-Bohouth St., Dokki 12622 Giza Egypt

## Abstract

Alzheimer's disease (AD) is a multifactorial neurodegenerative disorder characterized by progressive cognitive impairment and the accumulation of amyloid-β (Aβ) peptides. In this study, a novel series of triazole and phosphazine derivatives were synthesized and evaluated for neuroprotective activity in an aluminum chloride (AlCl_3_)-induced rat model of AD. Among the synthesized compounds, 3a, 6a, and 6c were structurally characterized and selected for *in vivo* biological evaluation. Behavioral, biochemical, molecular, and histopathological assessments were conducted to determine their efficacy, with Rivastigmine used as a reference drug. Compounds 3a and 6c significantly improved cognitive and memory performance, decreased Aβ_1–42_ production, and reduced reactive oxygen species (ROS) generation. Furthermore, both compounds inhibited the activation of JNK and Puma, promoted Beclin-1 expression, and activated Wnt/β-catenin signaling, as evidenced by increased expression levels of Wnt7a, β-catenin, LRP6, and FZD4, alongside decreased expression levels of GSK-3β and BACE1. Molecular docking studies supported these findings, revealing strong binding affinities of the active compounds, particularly 3a, to the JNK3 active site. Molecular dynamic simulations were performed on the best docking pose of the most potent compound 3a to confirm the formation of a stable complex with JNK3. Compounds 3a, 6a, and 6c demonstrated favorable pharmacokinetic profiles, with predicted good oral bioavailability, blood–brain barrier permeability, and non-substrate behavior toward P-glycoprotein. They are expected to maintain therapeutic availability in systemic circulation, as indicated by the predicted plasma protein binding below 90%, moderate to high steady-state volume of distribution, and lack of substrate affinity for cytochrome P450 enzymes CYP2C9 and CYP2D6. These results suggest that compounds 3a and 6c may serve as promising multi-target therapeutic candidates for AD by modulating oxidative stress, apoptosis, autophagy, and Wnt/β-catenin signaling pathways.

## Abstract 2

New phosphazine and triazole derivatives were synthesized and evaluated as potential anti-Alzheimer's agents. The compounds modulate ROS/JNK and Wnt/β-catenin pathways, showing promising neuroprotective activities.

## Graphical abstract

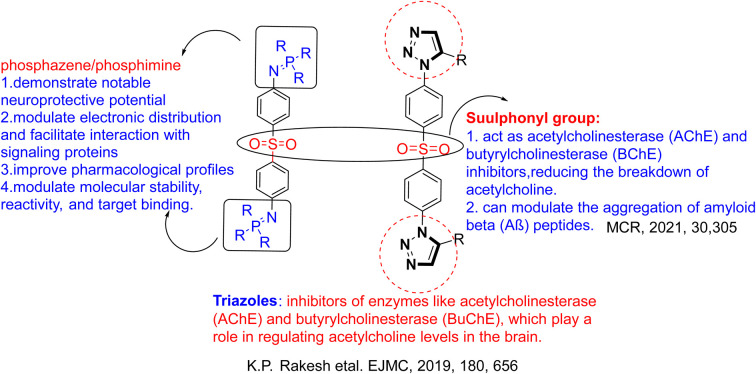

## 1. Introduction

Alzheimer's disease is a progressive age-associated neurodegenerative disease which represents 60–80% of all dementia cases.^1^ It is linked to cognitive dysfunctions including social deterioration, dementia and behavioral abnormality.^2^ AD is a multifactorial illness, which the exact etiology and developing mechanisms are not fully understood. Nonetheless, there are three key histopathological hallmarks for AD including the building-up of hyper-phosphorylated tau protein inside the neurons, the accumulation of the incorrectly cleaved β-amyloid protein (Aβ) in the brain that then clusters together forming the senile plaques extracellularly between neurons, and finally synapses and neurons loss.^3^

Aluminum (Al) is a well-known neurotoxin that plays a key role in AD onset and progression.^4^ It primarily accumulated in the frontal cortex and hippocampus, brain regions principally susceptible to AD.^5^ Prolonged Al exposure can result in neurochemical, neurobehavioral and neuropathological alterations, which hinder the learning capability in various animal studies.^6–8^ Moreover, Al persuades cytoskeleton proteins misfolding, leading to tau neurofibrillary tangles and amyloid beta plaques formation in the brain.^9^

In AD brains, building up of Aβ_1–42_ fibrils are considered the instigator for neurodegenerative pathology *via* a cascade of events involving neurotoxicity and oxidative stress exhibited with augmented reactive oxygen species (ROS) generation.^10^ This excessive ROS production causes activation of several molecular signaling pathways involving the mitogen-activated protein kinase [MAPK]^11^ that participate in cell growth modulation, differentiation, and cell death.^12^ It has been illustrated that c-Jun N-terminal kinase (JNK), a member of MAPKs pathway, is intricate in the pathogenesis of AD and could be activated in response to Aβ accumulation^13^ participating in its-induced neuronal damage.^14^ JNK instigation causes excessive Aβ deposition, activating a positive feedback loop and accelerating the progression of AD.^15^ JNK has a crucial role in controlling mitochondrial apoptotic pathway through modulating the Bcl-2-associated proteins^16^ including p53 up-regulated modulator of apoptosis (Puma)^17^ as a result of ROS production and cellular stress.^18^ It has revealed that Puma, is required for neuronal degeneration and axonal death prompted *via* Aβ treatment.^19^ Research has confirmed that additional significant pathological character of AD is autophagy dysregulation, a cell self-cleaning mechanism through degradation of misfolded proteins and impaired organelles.^20^ Accumulating Aβ and JNK activation cause dysfunction of autophagy *via* depressing Beclin-1level, an autophagy protein involved in the preinitiation complex formation leads to further Aβ plaques deposition and exacerbating neuro-inflammation.^21^ Therefore, motivating autophagy pathways epitomizes a prospective therapeutic approach for AD.^20^

Wnt/β-catenin signaling is activated when Wnt proteins bind to FZD/LRP, which inhibits GSK-3β.^22^ One of the two main kinases that cause β-catenin phosphorylation is GSK-3β, whose activation causes β-catenin to become phosphorylated and degraded.^23^ It has been shown that brains of AD patients have higher GSK-3β activity,^24^ which may be due to the AD brain downregulation of LRP6 and upregulation of DKK1. In the prefrontal cortical lobe structures of human AD brains, a recent study demonstrates that a significant decrease in β-catenin protein levels is inversely associated with increased activation of GSK-3β,^25^ supporting the fact that GSK-3β activity is linked to Wnt/β-catenin signaling in AD brain. Additionally, GSK-3β is a crucial kinase for tau phosphorylation, and its overactivation is closely related to plaque-associated microglial-mediated inflammatory responses, tau hyperphosphorylation, Aβ deposition, and memory impairment.^24,26^ Thus, the production of Aβ is eliminated when the BACE1 gene is deleted in the germline. BACE1 affects the buildup of Aβ in cells and synapses; it is a molecule that is directly related to synaptic functioning.^27–29^ The majority of BACE1 expression occurs in the brain, and neurons express it abundantly.^30^ In the brains of AD patients and mice models, accumulation of BACE1 is shown in both normal and dystrophic presynaptic terminals around amyloid plaques. Probably this vicious loop increases the generation of Aβ close to synapses. Consequently, it makes sense to believe that BACE1 inhibition would mitigate Aβ-mediated synaptic dysfunctions and might be beneficial for AD patients.^31^

Organophosphorus compounds have numerous applications in medicine.^32^ Certain organophosphorus compounds function as acetylcholinesterase inhibitors (AChEIs), which are pivotal in AD treatment. By inhibiting acetylcholinesterase enzyme, these compounds increase acetylcholine levels in the brain, thereby enhancing cholinergic neurotransmission, a pathway often impaired in AD patients. FDA-approved AChEIs such as donepezil, Rivastigmine, and galantamine are commonly prescribed to alleviate cognitive symptoms associated with AD. These medications are designed for selectivity, effective blood–brain barrier penetration, and manageable dosing schedules.^33–35^

**Fig. 1:**
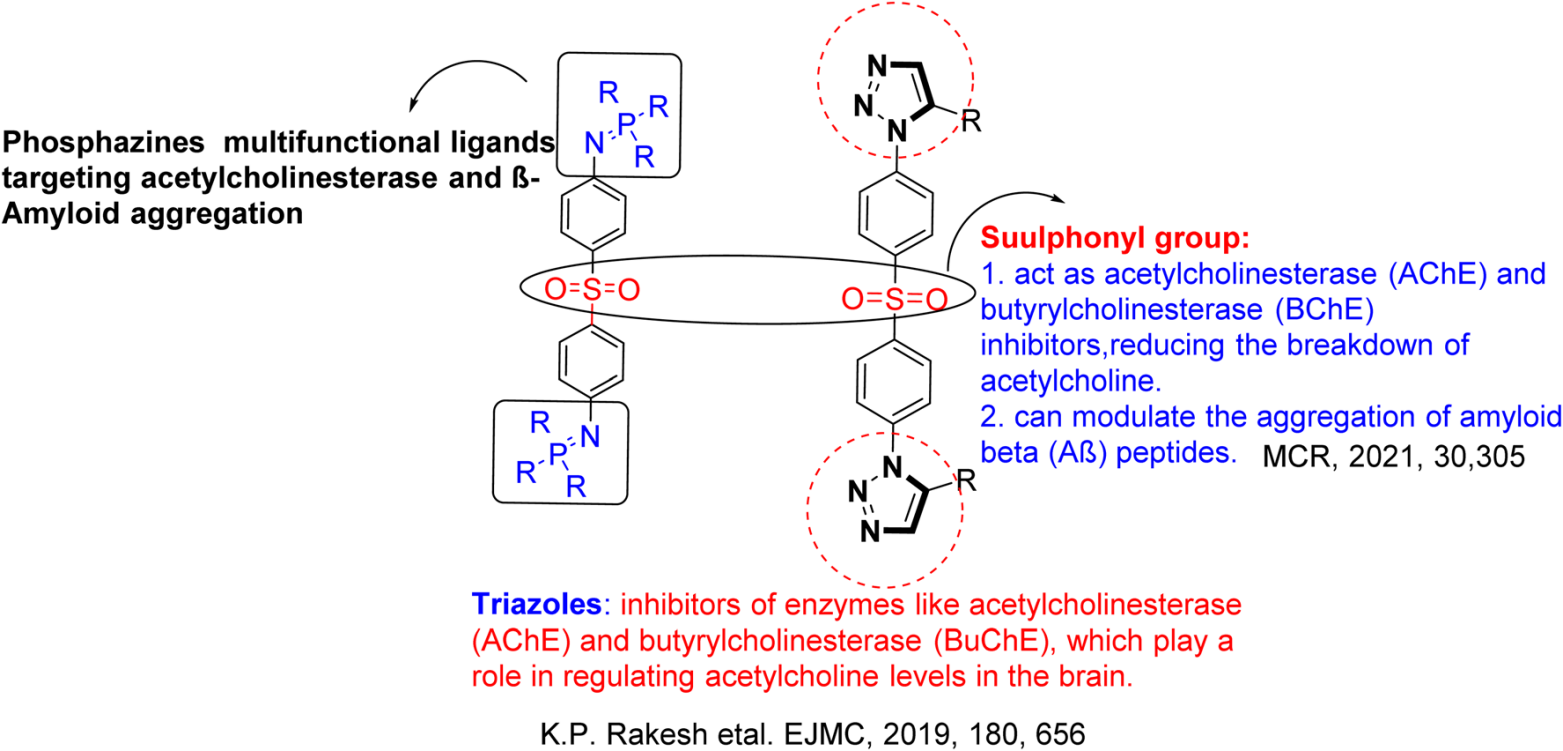
Design of targeted compounds.

The targeted molecule was selected through selection of active groups such as sulphonyl, triazole, and phosphazine groups to facilitate specific interactions within the active site, thereby maximizing biological efficacy while minimizing off-target effects. Phosphazines are multifunctional ligands designed to target both acetylcholinesterase (AChE) and β-amyloid (Aβ) aggregation.^36^ The sulfonyl moiety contributes to AChE and butyrylcholinesterase (BChE) inhibition, thereby reducing acetylcholine hydrolysis and modulating Aβ peptide aggregation.^37^ Triazole units further enhance activity by inhibiting AChE and BChE, enzymes critical for maintaining acetylcholine levels in the brain^38^ (Fig. 1).

Recent research into Alzheimer's disease has explored novel organophosphorus compounds (OPs) for their potential therapeutic applications, particularly focusing on their roles as acetylcholinesterase inhibitors and modulators of amyloid-beta aggregation.^36,39^

The aim of this research was to design, synthesize, and evaluate novel phosphazine and triazole derivatives for their potential therapeutic effects in treating Alzheimer's disease in an AlCl_3_-induced AD rat model. The study focused on investigating compound stability to modulate cognitive deficits and mitigate key signaling pathways ROS/JNK and Wnt/β-catenin implicated in AD pathology, through behavioral, biochemical, molecular, and histopathological analyses. Additionally, evaluating the pharmacokinetic (PK) properties and molecular docking studies were conducted to elucidate the binding interactions of the synthesized compounds with JNK3, further validating their mechanistic potential. Molecular dynamic simulations were performed to investigate the stability of the formed complex between the most active compound and JNK3.

## 2. Results and discussion

### 2.1. Chemistry

**Scheme 1:**
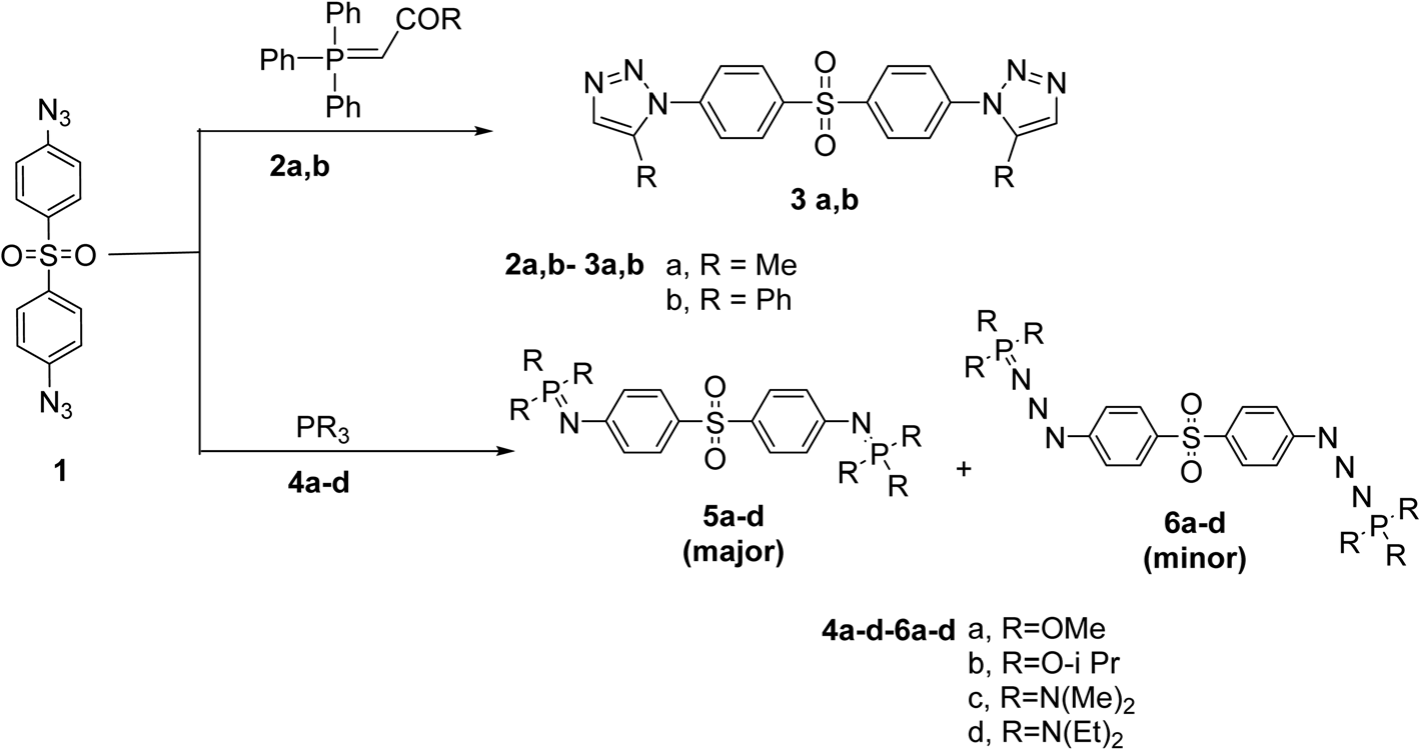
Synthesis of triazole 3a,b, phosphazine 5a–d and phosphazide 6a–d derivatives.

4,4′-Sulfonylbis(azidobenzene) (1) reacted with Wittig reagents namely, 1-(triphenyl-λ^5^-phosphaneylidene)propan-2-one (2a) and 1-phenyl-2-(triphenyl-λ^5^-phosphaneylidene)ethan-1-one (2b) under stirring for 2 h in toluene to get triazole derivatives 3a,b in 50–65% yield, respectively (Scheme 1). The mechanism of this reaction was previously described.^40,41^

Also, compound 1 reacted with trialkylphosphite 4a,b and tris(dimethylamino)phosphine 4c,d under stirring for 30 min in toluene to afford phosphazine derivatives 5a–d as major compounds with separation of phosphazide derivatives 6a–d as minor compounds (*cf.* Experimental, Scheme 1).

Structures of triazole 3a,b, phosphazine 5a–d and phosphazide 6a–d derivatives were confirmed *via* spectroscopic analyses (IR, NMR and mass spectra) (*cf.* Experimental).

The ^1^H NMR spectrum (500 MHz, DMSO-*d*_6_) of compound 3a displayed characteristic signals: doublets for the aromatic protons at *δ* 8.28 and 7.95 (*J* = 8.6 Hz), a singlet for the triazole proton at *δ* 7.74, and a singlet for the methyl group at *δ* 2.38. Furthermore, the ^13^C NMR spectrum (126 MHz, DMSO-*d*_6_) exhibited signals corresponding to the triazole carbons at *δ* 125.4 and 140.6 ppm, the aromatic carbons at *δ* 129.1, 133.8, 134.4, and 140.0 ppm, and the methyl carbon at *δ* 8.8 ppm (*cf*. Experimental, SI file).

A comparative analysis of phosphazine and phosphazide derivatives was conducted using compounds 5a and 6a as representative examples. In the ^13^C NMR spectrum of compound 5a (126 MHz, CDCl_3_), a distinct signal at *δ* 145.6 ppm was observed, corresponding to the aromatic C–N–P linkage. This resonance is absent in the spectrum of the corresponding phosphazide 6a, providing a clear spectroscopic distinction between the two structural classes (see Experimental section and SI file).

**Scheme 2:**
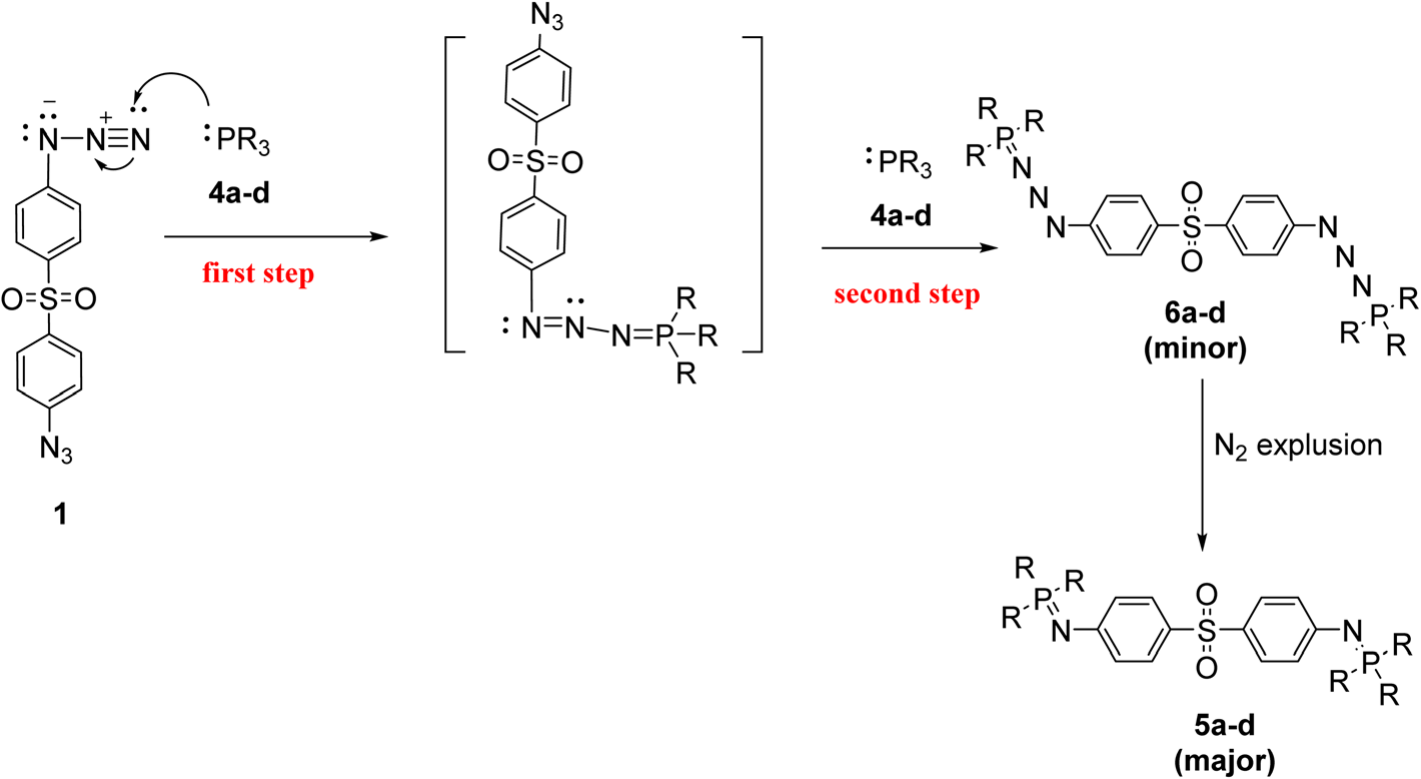
Proposed mechanism for phosphazine 5a–d and phosphazide 6a–d formation.

Mechanistically, the formation of phosphazides 6a–d is proposed to proceed *via* an electrophilic attack, in which the trivalent phosphorus center of reagents 4a–d attacks the terminal nitrogen of the azide group in compound 1. This process is facilitated by electronic interactions between an electron-deficient substituent on the azide and an electron-rich phosphorus atom. Either electron-donating or electron-withdrawing groups on the azide or phosphorus moiety, as shown in Scheme 2 stabilize the resulting phosphazides. Literature precedents, including stable analogues such as (Me_2_N)_3_PN_3_Ph and Ph_3_PN_3_Tos, support the feasibility of this transformation and highlight the stabilizing role of aryl substituents.^42–45^ Subsequent thermally or chemically induced nitrogen extrusion from phosphazides 6a–d affords the corresponding phosphazine derivatives 5a–d in good yields, completing the transformation (Scheme 2).

In addition, phosphazine 5a–d formed by the Staudinger-aza-Wittig reaction, these are mono-condensation products involving one equivalent of phosphorus reagent per azide.

In some cases, especially under extended reaction times or excess phosphorous reagent, further condensation occurs forming triazene-like phosphorus(v) derivatives (6a–d). These compounds arise from further insertion of phosphorus into the azide or imine frameworks, potentially involving rearranged triazene-type structures stabilized by electron-rich P(v) substituents.

### 2.2. Behavioral assessment

The Morris Water Maze (MWM), the Novel Object Recognition (NORT), and the Y-Maze tests were employed to assess cognitive deficits in the rat AD model.

#### 2.2.1. Effect of Rivastigmine and compounds 3a, 6a, and 6c on Morris water maze test in AD-induced group

**Fig. 2:**
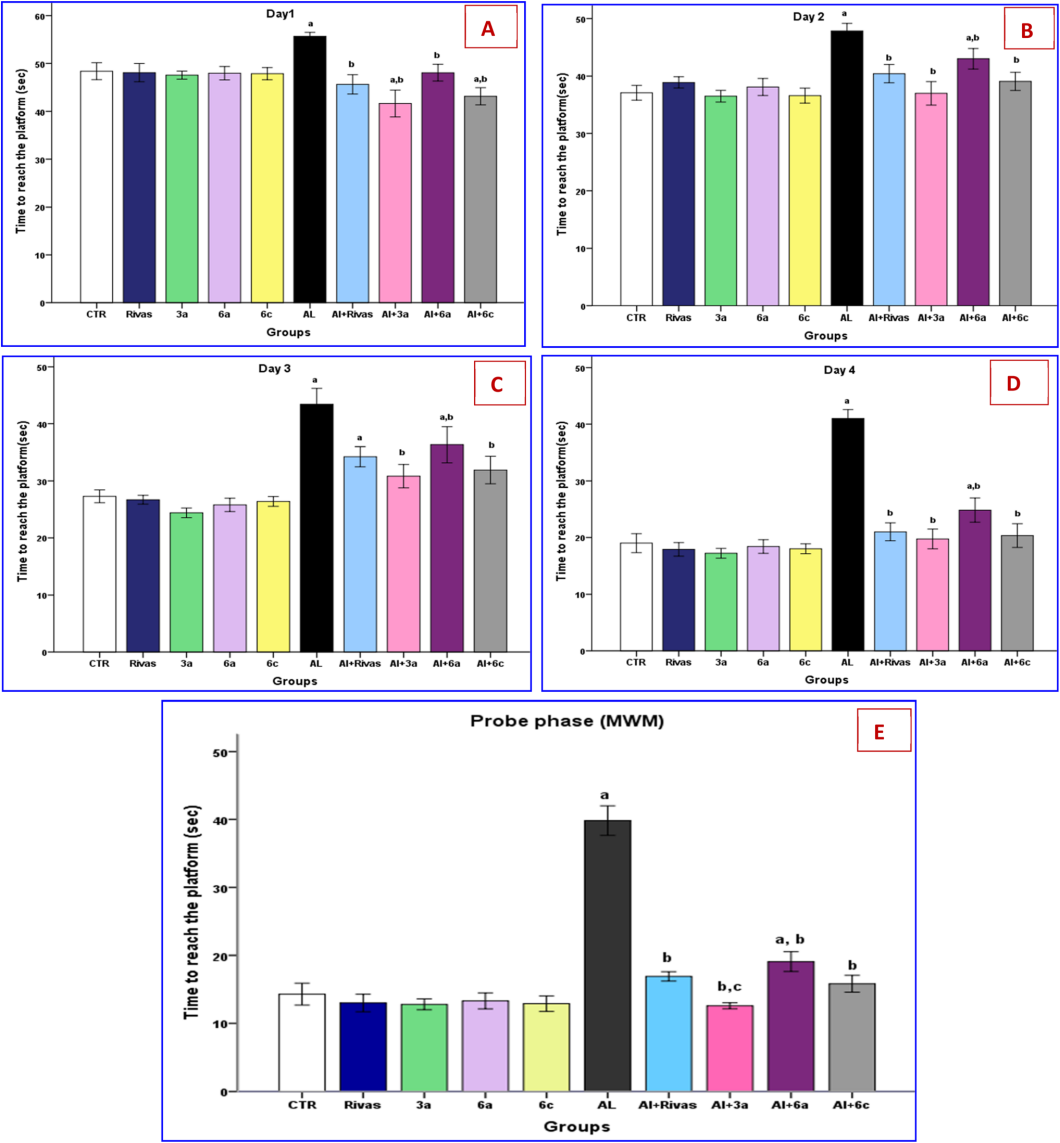
(A–E) Effects of treatment with Rivastigmine and novel synthesized chemical compounds [3a, 6a, and 6c] on escape latency during Acquisition phase (A, B, C and D) and probe phase (E) of Morris Water Maze (MWM) test in AD-induced group (*n* = 6). Data are denoted as mean ± SEM; ^a^*P* < 0.05 as compared to the normal control group, ^b^*P* < 0.05 as compared to AlCl_3_-treated group, ^c^*P* < 0.05 as compared to AlCl_3_ and Rivastigmine-treated group.

Using MWM, spatial memory and learning were evaluated by measuring the escape latency to find the platform through the acquisition and probe phases. The training/acquisition trials showed a change in the escape latency (time to reach the hiding platform). During the four-day water-maze training session, the escape latency progressively decreased, and statistically significant differences were observed among the study groups (Fig. 2A–D). Rats in the AlCl_3_-treated group exhibited the longest latency to reach the platform compared to all other groups. Statistical analysis revealed a significant increase (*p* ≤ 0.05) in the time required to locate the platform during the training trials, relative to the control (CTR) group, consistent with impaired spatial learning and memory associated with aluminum-induced neurotoxicity.^46–48^ Similarly, Zghari *et al.* stated that chronic aluminum exposure impairs spatial learning abilities and results in cognitive problems marked by memory impairment.^49^ Aluminum's impact on working memory becomes noticeable at 1 mg kg^−1^, whereas its effects on spatial learning performance begin at 0.25 mg kg^−1^ and peak at 1 mg kg^−1^. Nevertheless, treatment with Rivastigmine or newly synthesized compounds (3a, 6a, 6c) resulted in a significant decrease in the time to reach the platform during the acquisition phase.

During the probe phase of the MWM test, normal rats treated with different experimental drugs showed no significant difference in activity compared to the CTR groups. Meanwhile AlCl_3_ administered group spent significantly (*p* < 0.001) longer time to reach the platform in the probe session (39.8 s) than the CTR group (14.3 s) (Fig. 2E). Additionally, the AlCl_3_ evoked group had a significant (*p* < 0.001) increase in escape latency to reach the hidden platform in comparison to all treated groups. Our findings align with prior studies^47,48,50,51^ that reported that after administering Al, the time required to overtake the hidden platform in the MWM test significantly increased and spatial memory decreased. This could be because aluminum has a negative impact on the central nervous system (CNS) of mammals. It affects the neurotransmitter synthesis-related enzymes and slows synaptic transmission resulting in spatial memory impairment.^52^ Of note, the administration of the novel synthesized chemical compounds (3a, 6a, and 6c) and Rivastigmine improved the spatial memory of AD induced rats, this was reflected by an obvious reduction in time spent reaching the hidden platform (12.5, 19, 15.8 and 16.9 s, respectively) than AD only (as shown in Fig. 2E). These results agree with the findings of Abdel-Atty *et al*.,^53^ who reported that rats treated with Rivastigmine for AD showed a significantly shorter escape latency time than those of AlCl_3_ group, suggesting that memory performance had improved. Of note, AD group treated with 3a has a considerable enhancement in spatial memory in MWM test compared to Al + Rivas group.

In this regard, Shourkabi *et al*.,^54^ assessed the learning and memory level changes using the MWM test and found that the model (Alzheimer's rats) and control (normal rats) groups showed a significant decrease in the distance walked to locate the platform over the course of four days. On the other hand, Alzheimer's rats traveled farther than normal rats. The probing test on the fifth day showed that the control group spent more time on the target platform while the model group spent less time there.

#### 2.2.2. Effect of Rivastigmine and compounds 3a, 6a, and 6c on novel object recognition test in AD-induced group

**Fig. 3:**
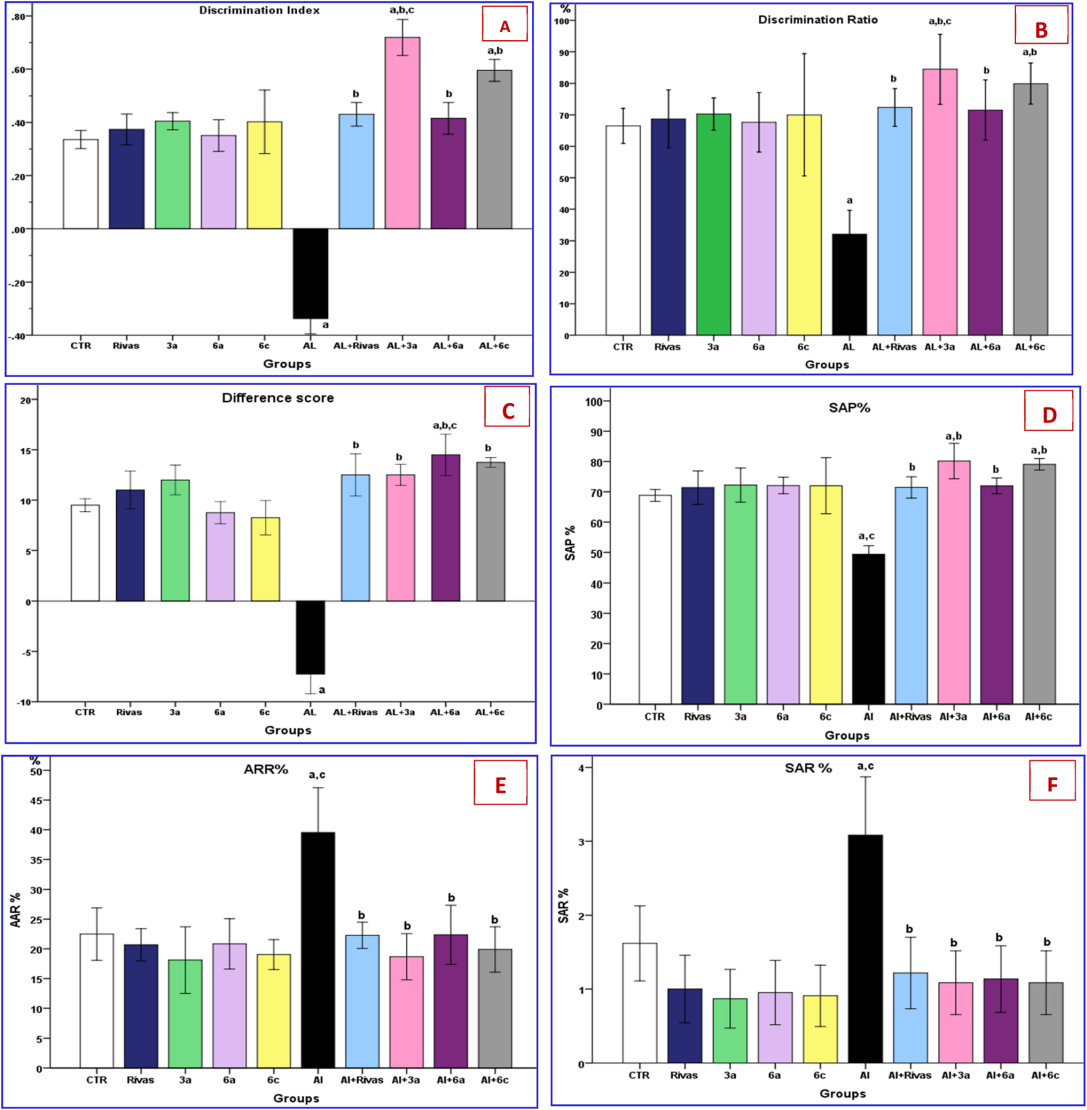
(A–F) Effects of treatment with Rivastigmine and novel synthesized chemical compounds [3a, 6a, and 6c] on novel object recognition (*n* = 6) where, (A: discrimination index; B, discrimination ratio; C: difference score) and Y-maze tests (*n* = 6) where, (D: SAP%; E: AAR%; F: SAR%). Data are denoted as mean ± SEM; ^a^*P* < 0.05 as compared to the normal control group, ^b^*P* < 0.05 as compared to AlCl_3_-treated group, ^c^*P* < 0.05 as compared to AlCl_3_ and Rivastigmine-treated group. [SAP% = spontaneous alternation performance; AAR = alternate arm return; SAR = same arm return].

Novel object discrimination test was used to assess the animals' capacity to memorize previously-visited objects and to explore novel objects,^55^ as shown by DI, DR and DS (Fig. 3A–C). According to the results, the AlCl_3_ induced AD group had significantly lower DI, DR and DS (−200.5%, −51.8%, and −176.3%, respectively) compared to the control group, indicating the decreased recognition memory (*P* = 0.000). These findings were inconsistent with previous studies.^56,57^ Additionally, it was reported that scopolamine-induced Alzheimer's disease (AD) rats exhibited a significantly lower recognition index (RI%) compared to the control group (*P* < 0.001).^58^

On the other hand, AlCl_3_-induced short-term memory impairment was ameliorated by treatment with Rivastigmine and novel synthesized compounds (3a, 6a and 6c), as evidenced by a significant (*P* = 0.000) increase in DI, DR and DS compared to the AD group. In this concern, Abdel-Aal *et al.*,^48^ found that AD rats that received Rivastigmine spent much more time investigating the new object; *p* < 0.0001. Nevertheless, Gothwal *et al.*,^56^ demonstrated that the discrimination index for the Al + Rivastigmine and Al + PAMAM-Rivastigmine treated groups were not considerably greater than that of the Al group however, the discrimination index of PAMAM-Lf-Rivastigmine group showed a substantial increase compared to the Al group indicating the comprehensive efficacy of dendrimeric formulations in the animal model of AD-induced memory.

Interestingly, the Al+3a group had an obvious increase in DI and DR (0.71 and 84.45%, respectively) in comparison with the Al group treated by Rivastigmine (0.43 and 72.36%, respectively) as illustrated in (Fig. 3A and B).

#### 2.2.3. Effect of Rivastigmine and compounds 3a, 6a, and 6c on Y maze test in AD-induced group

To evaluate the short-term spatial and working memory, the Y-maze test was performed.^59^Fig. 3D–F shows a significant decrease (−28.19%) in spontaneous alternation percentage (SAP) and a considerable increase of AAR & SAR (82.9% and 91.3%, respectively) in the AlCl_3_ group (AD group) in comparison with the CTR group. This agrees with the finding of Khalil *et al.*^57^

Likewise, the previous studies reported that Al considerably reduced the SAP%^60,61^ and spontaneous alternations by1.45-fold in the Y-maze test^62^ compared to the group of normal rats.

Concurrently, treatment with Rivastigmine, 3a, 6a and 6c compounds modulates the memory impairment provoked by AlCl_3_ by significantly (*P* < 0.0001) increasing the SAP% (46.9%, 62.2%, 45.6%, 55.5%, respectively) in addition to, decreasing the SAR and AAR compared to the AlCl_3_ group. There was a distinction of SAP between AD-induced groups that were treated by 3a & 6c compounds (10%, 5.3% respectively) than Al + Rivastigmine as well as the Al+3a group which had a marked decrease (−16.1%) in AAR than Al + Rivas group. Our results are in the same line with Bais *et al.*^63^ who found that Rivastigmine improved the memory of Al treated rats. No marked alternation in SAP, SAR and AAR of the control group and normal treated rat groups was noticed.

The aforementioned results revealed that AlCl_3_ poisoning resulted in deficits in cognitive abilities in the Morris Water Maze and novel objective recognition tests as well as memory performance in the Y-maze test, consistent with Khalil *et al.*^57^ This is demonstrated by a significant increase in escape latency, AAR & SAR and a notable decline in DI, DR, DS & SAP. These results align with prior studies.^50,57,64^ The dispersion of the hippocampus circuit and its numerous connections appears to be the cause of these behavioral declines in memory and learning.^53^ The change in cognitive behavior can be improved through treatment with different drugs such as Rivastigmine. In line with previous studies, Rivastigmine had beneficial effects on memory and learning in the chronic d-galactose-induced accelerated aging rat model,^65^ in the streptozotocin rat model of AD^66^ and AlCl_3_ induced AD.^48,56,61,63^ Rivastigmine is a well-known reversible AChE inhibitor that enhances cognitive performance and brain plasticity by preventing the AChE enzyme from metabolizing ACh.^67^

In our study, we examined the impact of different newly synthesized compounds (3a, 6a & 6c) which revealed their better efficacy in enhancing cognitive compared to the Rivastigmine. The potent efficiency of these synthesized compounds may be attributed to their multitarget directed ligands including cholinergic transmission enhancement,^68^ disruption of amyloid-beta aggregation^69,70^ in addition to antioxidant activity *via* free radical scavenging and mitigating oxidative stress.^71^

#### 2.2.4. Effect of Rivastigmine and compounds 3a, 6a, and 6c on amyloid beta 1–42 concentration in AD-induced group

**Fig. 4:**
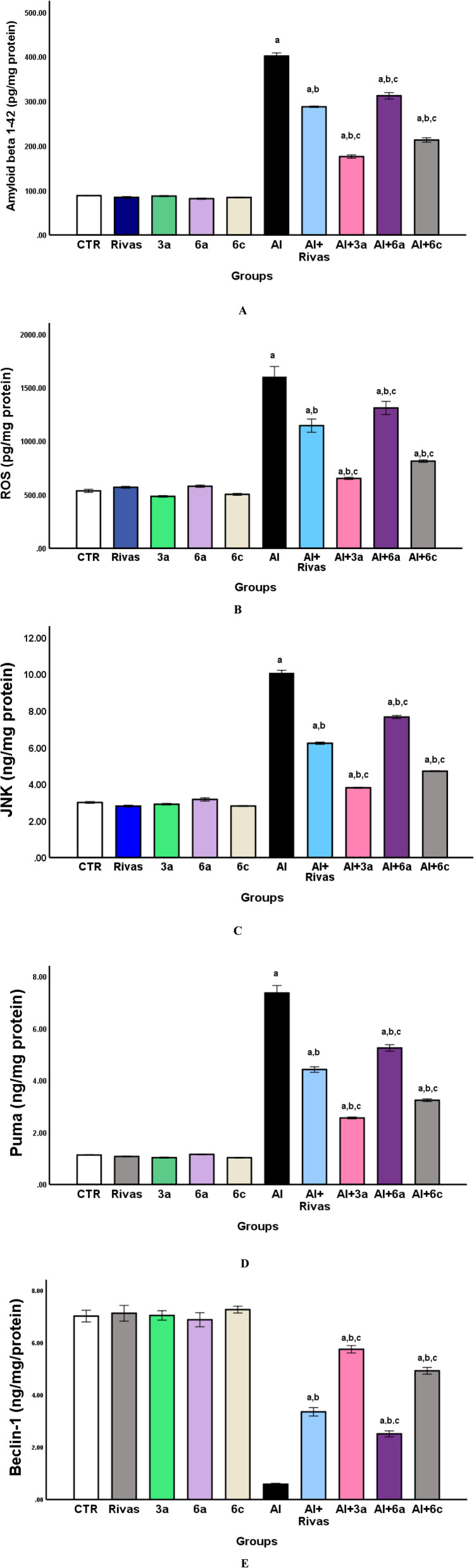
(A–E) Effects of treatment with Rivastigmine and novel synthesized chemical compounds [3a, 6a, and 6c] on concentrations of amyloid beta 1–42 peptide (A), ROS (B), JNK (C), Puma (D) and Beclin-1 (E) in AD-induced group(*n* = 6). Data are denoted as mean ± SEM; ^a^*P* < 0.05 as compared to the normal control group, ^b^*P* < 0.05 as compared to AlCl_3_-treated group, ^c^*P* < 0.05 as compared to AlCl_3_ and Rivastigmine-treated group.

Amyloid-persuaded neurotoxicity is considered a vital factor in the AD etiology.^72^ In the current study, comparing with the CTR group, normal rats administrated with different treatments under investigation manifested no substantial changes in rat's brain Aβ 1–42 level. While AlCl_3_-evoked rats demonstrated a marginal increase in the level of this marker by 354.5% (Fig. 4A), in contrast to the corresponding control rats. This result agrees with Kaur *et al.*^73^ and Ali *et al.*^74^ reported that the Aβ_1–42_ brain level was significantly augmented in AlCl_3_-intoxicated rats as equated to the control group. This observation could be attributed to Al capability to overexpress the amyloid precursor protein (APP) and β-site amyloid precursor protein cleaving enzyme (BACE1) protein levels, speculating that Al boost the cleavage impact of BACE1 on APP to upsurge brain Aβ production^75^ which then aggregate to stimulate β-amyloid plaques generation.^76^

On the other hand, to examine the effects of Rivastigmine and the newly synthesized compounds against the Aβ_1–42_ peptide production, we compare all treated groups with AD-triggered group. All of them considerably recovered changed brain Aβ_1–42_ level by 28.4% for Rivastigmine, 56.2% for 3a, 22.2% for 6a, and 46.9% for 6c (Fig. 4A). Ismail *et al.*^77^ disclosed that co-administration of Rivastigmine with AlCl_3_ exerted marked decline in gene expression level of BACE1, decreasing Aβ production level. Moreover, the aforementioned findings illustrated that the examined compounds produced beneficial impact against formation of Aβ peptides in a statistically significant manner clarifying their neuroprotective action. 3a compound manifested the best outcome; this could be referred to the presence of triazole a five-member heterocyclic ring with two carbon and three nitrogen atoms which have broad biological activities.^78^ In 2019, Kaur and colleagues developed a series of triazole-based compounds that act as inhibitors for Aβ_1–42_ aggregation and as anti-AD agents.^79^ Furthermore, ten new triazinyl-1,2,4-triazines bearing pendant aryl phenoxymethyl-1,2,3-triazoles were examined as a treatment of AD with multifunctional characters.^80^ The supreme potent derivative (*E*)-3-(2-(4-((1-(4-chlorobenzyl)-1*H*-1,2,3-triazol-4-yl)methoxy)benzylidene)hydrazineyl)-5,6-diphenyl-1,2,4-triazine displayed promising *in vitro* BACE1 inhibitory action, neuroprotection against the Aβ25-35-prompted injury and metal chelating capability.^80^ Also, Rastegari *et al.*,^81^ created and tested a series of novel 1,2,3-triazole chromenone carboxamide derivatives for their *in vitro* cholinesterase inhibitory action. The optimal compound *N*-(1-benzylpiperidin-4-yl)-7-((1-(3,4-dimethylbenzyl)-1*H*-1,2,3-triazol-4-yl)methoxy)-2-oxo-2H-chromene-3-carboxamide repressed AChE activity significantly and exhibited as a good BACE1 inhibitor.^81^ The above-mentioned reports support our findings in reducing Aβ 1–42 production due to presence of triazole rings. Additionally, in comparison with Al + Rivas group, treatment with 3a and 6c compounds revealed superior potency than Rivastigmine in reducing brain Aβ_1–42_ level (Fig. 4A).

#### 2.2.5. Effect of Rivastigmine and compounds 3a, 6a, and 6c on ROS and JNK concentrations in AD-induced group

Prominent intracellular ROS generation is an oxidative stress marker that related to AD development and progression.^82^ In the present study, relative to the CTR group, normal rats administrated with various investigated treatments displayed no significant changes in rat's brain ROS level. However, AlCl_3_ gavage caused a substantial raised brain ROS level by 198.51% (Fig. 4B). This finding was in cohort with the prior literature noticed a substantial increased ROS levels in Alzheimer's rat's cortex and hippocampus persuaded by Al ingestion when compared to control ones.^83^ The mechanisms involved Al neurotoxicity is poorly recognized, but it is well known that Al stimulates ROS production and decrease antioxidant defense system instigating obvious oxidative stress in the brain.^84,85^ Al is a pro-oxidant both *in vivo* and *in vitro* but not a redox-active metal. Inside cells Al^3+^ can, on one hand, create the Al-superoxide complex (AlO_2_^2+^), which facilitates the direct O_2_˙^–^ activity and exacerbates oxidative damage by promoting the formation of HO.^86^ On the other hand, through the Fenton reaction, Al converts Fe(iii) to Fe(ii), causing oxidative damage.^87^ Additionally, it is believed that Aβ causes oxidative stress by producing too many ROS, which in turn, harms crucial macromolecules and mitochondria enhancing further ROS production.^13^

Conversely, relative to Al group, Rivastigmine gavage or injection with different synthesized compounds, markedly diminished brain ROS level [28.44% for Rivas, 59.25% for 3a, 18.14% for 6a, and 49.15% for 6c] (Fig. 4B). Our results confirmed that Rivas and tested compounds have an antioxidant activity as they effectively attenuated intracellular ROS formation; however, 3a compound manifested the best effect. In accordance to our findings the earlier report by Gupta *et al.*,^88^ possessed Rivastigmine had anti-oxidative activity. Likewise, Dalvi *et al.*^89^ indicated that a triazole-based compound exposed a promising *in vivo* outcomes owing to its anti-oxidant/anti-inflammatory properties *via* ROS scavenging and metal chelation as Zn^2+^, Cu^2+^, and Fe^3+^ as a result of the presence of heteroatom. In addition, other recent study by Koçak Aslan *et al.*^90^ found that triazole derivatives bearing the naphthalene moiety displayed a substantial antioxidant potency, moderate inhibition capacity against the aggregation of amyloid-β1-42, and momentous neuroprotective impact on H_2_O_2_ and amyloid-β_1-42_ induced SH-SY5Y cell injury. Moreover, in comparison with Al + Rivas group, treatment with 3a and 6c compounds revealed superior potency than Rivastigmine in reducing brain ROS level (Fig. 4B).

JNK signaling pathway has been proposed to be involved in pathology of AD and associated memory impairment.^91^ In AD brains, JNK phosphorylates tau and APP, which ultimately leads to the development of intra-neural neurofibrillary tangles and extra-neural senile plaques.^92^Fig. 4C illustrated that treatment of healthy animals with Rivas, 3a, 6a, or 6c compounds don't show any substantial impact on the brain level of JNK as paralleled to the CTR group, nonetheless, oral AlCl_3_ administration caused a significant elevation in its level by 234.84%. Our results agree with Zhang *et al.*^93^ verified AlCl_3_ administration augmented the JNK and phosphorylated-JNK (p-JNK) protein levels, as well as enhanced the ratio of p-JNK/JNK protein expressions within the hippocampus of exposed rats. It had been noted that the aggregation of ROS instigated by Al initiated MAPKs activation (p-JNK up-regulation) and blocked the entry of NF-κB/p65 to the nucleus.^94^ Besides, previous literature confirmed that formation of Aβ by Al enhances JNK activation.^13^

Contrarily, Fig. 4C presented that gavage with Rivastigmine or synthesized compounds injection to AD-induced group induced a momentous inhibition in JNK amount in their brain tissues as compared to Al-exposed group [38.03% for Rivas, 62.17% for 3a, 23.83% for 6a, and 53.19% for 6c]. Accordingly, it is obvious that treatment with various analyzed compounds under study abolished the increase in level of JNK in a statistically significant manner. Chambers reported a highly selective and orally bioavailable triazole-based compound performing as JNK inhibitor for dopaminergic neurons protection *in vitro* and *in vivo* in Parkinson disease mouse (PD) model, proposing that this triazole-based JNK inhibitor can be a prospective therapeutic neuroprotective agent in PD treatment.^95^ Additionally, medicinal drugs based-triazole derivatives have been widely inspected, as anti-convulsant, anti-inflammatory as well as anti-neuropathic.^96^ Furthermore, compared to Al + Rivas group, injections with 3a and 6c compounds under investigation revealed superior activity than Rivastigmine in decreasing JNK brain level (Fig. 4C).

#### 2.2.6. Effect of Rivastigmine and compounds 3a, 6a, and 6c on Puma and Beclin-1 concentrations in AD-induced group

Stimulation of programmed cell deaths, particularly apoptosis, and autophagy failure critically influences AD pathogenesis.^20^ In this report, relative to control group, examined drugs manifested no discernible alteration in Puma brain level when administrated to normal rats, meanwhile; Al group displayed considerable rising of brain concentration of this apoptotic marker by 7.37% (Fig. 4D). Feng *et al.*^97^ confirmed that Puma activation is critical for Aβ-triggered neural apoptosis by demonstrating a significant enhancement in Puma expression level in Aβ-treated SHSY5Y and primary neurons as well as in the transgenic mice hippocampi (APP/PS1). Numerous investigations indicated that the ROS/JNK/p53 pathway is of pivotal importance in the process of apoptosis^98^ and ROS generated by Al^99^ triggers JNK activation to persuade apoptosis *via* modulating the expression of various apoptotic factors^100^ involving apoptotic p53 protein, a powerful apoptotic inducer and a regulator for Puma transactivation^98,101^ that motivate Puma production.

As depicted in Fig. 4D a substantial deterioration in brain Puma level of rats cured with different drugs under study by 40% for Rivas, 65.24% for 3a, 28.72% for 6a, and 56% for 6c as equated with the Al group. Puma ablation might be helpful for curbing excessive apoptosis related to AD. Consequently, as Puma represent an essential sensor for apoptosis stresses, Puma could be a promising drug target for neurodegenerative diseases.^101^ Consistent with our findings, Gupta *et al.*^88^ suggested that Rivastigmine has a significant ability to inhibit neural apoptotic markers related to AD in both cortex and hippocampus. Besides, owing to the biological significance of triazole and its derivatives, they have drawn a lot of attention recently. Suppression of the JNK pathway by triazole derivative^95^ could offer a momentous defense against oxidative stress-induced apoptosis.^102^ Numerous triazole scaffolds have been described to possess an extensive range of bioactivities, as anti-oxidation^103^ and neuroprotection.^104^ Likewise, compared to Al + Rivas group, treatment with 3a and 6c compounds exposed a superior potency than Rivastigmine in hindering brain Puma level (Fig. 4D).

Autophagy offering a therapeutic potential in the neurodegenerative diseases treatment, by serving as a pathway for clearing abnormally aggregated proteins and damaged organelles.^21^ This process is controlled by several proteins including Beclin-1, a member of BH3-only domain protein family and a key player in autophagy process which initiates the autophagosomes formation by binding with autophagy precursors.^105^ In AD, Beclin-1 expression diminished in the brain, causing weakened autophagy activity.^106^

As denoted in Fig. 4E, brain level of Beclin-1 wasn't changed markedly in normal rats received different therapies under investigation compared to the untreated one. However, AD-induced group illustrated a significant downregulation in concentration of this autophagic marker by 91.6% compared to the CTR group causing autophagy failure. Naseri and colleagues have shown considerable diminution in Beclin-1 expression level in Aβ 1-42-induced AD model comparing with the control group.^20^ Autophagy is suggested to mitigate Al neurotoxicity, and malfunction of this process causing an increase in detrimental proteins.^107^ Sun *et al.*,^21^ presented that autophagy was downstream of JNK-p38MAPK and JNK activation by Al-prompted ROS which mediated the generation and aggregation of Aβ protein *via* inhibiting autophagy.^92^ Additionally, marked Beclin-1 downregulation causes altering APP metabolism and shifts it to generate other subcellular compartments as Aβ peptides.^108^ Autophagy can mediate the transport of BACE1 to reduce the production of Aβ^109^ in AD animal models.

On the other hand, treatment of AD-induced group with various examined compounds clarified enhanced brain level of Beclin-1 comparing with untreated corresponding group [Al group] by 470% for Rivas, 877% for 3a, 327% for 6a, and 737% for 6c (Fig. 4E). Given the vital role of autophagic pathways in clearance of aggregated and misfolded proteins, inhibiting inflammatory mediators, and repairing nerve damage,^21^ it could be represented as an ideal therapeutic candidate against AD.^110^ Interestingly, according to our data, the newly synthesized 3a compound manifested superior potency in enhancing the level of this autophagic protein. In the same line of our findings a triazole derivative augmented autophagy initiation and removal of nuclear Q79-EGFP aggregates *via* enhancing Beclin-1 level, LC3-I to LC3-II turnover and p62 degradation supporting autophagy instigation.^111^ In comparison with Al + Rivas group, treatment with 3a and 6c compounds revealed superior potency than Rivastigmine in improving brain Beclin-1level (Fig. 4E).

#### 2.2.7. **Effects of Rivastigmine and compounds**3a, 6a, and 6c**on Wnt/β-catenin pathway-related gene expression**

**Fig. 5:**
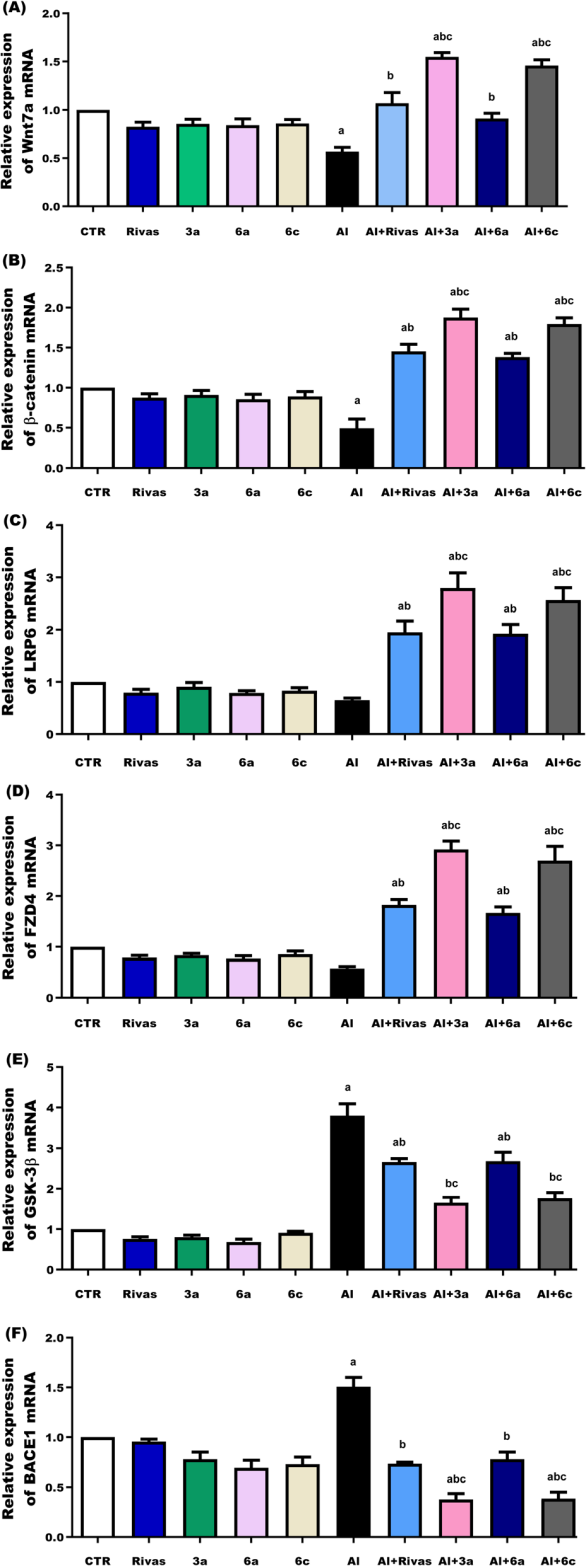
(A–F) Real-time polymerase chain reaction analysis of Wnt7a, β-catenin, LRP6, FZD4, GSK-3β, and BACE1 genes in the studied groups. Data were analyzed using one-way ANOVA followed by the Tukey–Kramer multiple comparison test. All the values were expressed as mean ± SEM (*n* = 6). ^a^*P* < 0.05 as compared to the normal control group, ^b^*P* < 0.05 as compared to AlCl_3_-treated group, ^c^*P* < 0.05 as compared to AlCl_3_ and Rivastigmine-treated group.

The Wnt/β-catenin pathway has been demonstrated to be downregulated in the pathogenesis of AD.^112^ Aβ causes a dysfunction of the Wnt/β-catenin pathway in AD. Aβ increases the levels of Dickkopf-1 (DKK1), which blocks Wnt, connects to LRP 5/6, hinders the Wnt/FZD complex, and decreases the interaction with Wnt signals. There has been an elevation of GSK-3 activity in the hippocampal regions of patients with AD.^113–115^ Increased GSK-3β activity inhibits the Wnt/β-catenin signaling system, impairing memory; decreases phosphorylated GSK-3β levels, which in turn increase β-catenin breakdown, which in turn raises Tau protein phosphorylation and decreases β-catenin and its gene expression.^116^ In the current study, no significant changes were observed in the expression levels of the investigated genes (Wnt7a, β-catenin, LRP6, FZD4, GSK-3β, and BACE1) in normal rats administered the tested treatments compared with the control group. In contrast, AlCl_3_ administration significantly altered gene expression profiles. Specifically, the mRNA expression levels of Wnt7a and β-catenin were significantly reduced (*P* < 0.05) to approximately 44% and 51% of the normal control group, respectively (Fig. 5A and B). Similarly, LRP6 and FZD4 expression levels were decreased to about 35% and 43% of control group, respectively (Fig. 5C and D). Conversely, GSK-3β gene expression was significantly (*P* < 0.05) higher in the AlCl_3_-treated group than in the normal control group by approximately 279% (Fig. 5E). These findings are consistent with those reported by Abu-Elfotuh *et al*.,^117^ who demonstrated that rats intoxicated with AlCl_3_ had a considerably greater level of GSK-3β, almost ten times higher than control rats, while also exhibiting a significantly lower amount of free β-catenin.

GSK-3β also contributes to the production and storage of Aβ *via* regulating the cleavage of the APP (Aβ precursor protein). According to its proteases, APP is cleaved in the brain *via* two distinct pathways: non-amyloidogenic and amyloidogenic.^118^ The non-amyloidogenic pathway is mediated by a α-secretase complex comprising γ-secretase, ADAM-10, and ADAM-17. The non-amyloidogenic pathway cleaves APP to create more degradable peptides.^119^ In contrast, the BACE1 (β-secretase) enzyme facilitates the amyloidogenic process. After processing by the γ-secretase complex, this enzyme produces Aβ peptides, which subsequently fibrilize and oligomerize to form Aβ deposits in the brain.^120^ It is well established that aluminum accelerates oxidative stress, Aβ oligomer cross-linking and deposition, and plaque development in the hippocampus and cortex of the brain. The cleavage of amyloid precursor protein (APP) by β-secretase, also referred to as beta-site amyloid precursor protein cleaving enzyme 1 (BACE1), and β-secretase enzymes starts the formation of Aβ.^121^ In the cortex and hippocampus, Aβ may easily diffuse across the brain parenchyma and trigger a series of harmful processes, including neuronal apoptosis/necrosis, oxidative stress generation, and neuroinflammation.^122,123^ In the current investigation, BACE1 gene expression was significantly (*P* < 0.05) higher in the AlCl_3_-treated group than in the normal control group by about 50% (Fig. 5F). These observations concur with those of Wang *et al.*,^124^ who stated that Al treatment enhanced BACE1 expression, contributing to an increase in brain levels of Aβ (1–42). Furthermore, according to Hamdan *et al.*,^125^ AlCl_3_ therapy dramatically raised the activity of BACE1 and APP levels in brain tissues, which led to an increase in Aβ levels in the ALAD group when compared to control groups (*P* < 0.05).

Stimulation of the Wnt/β-catenin signaling pathway is neuroprotective because it lowers the amount of synaptic protein, improves memory, suppresses BACE1 expression, and decreases Aβ deposits in the cortex and hippocampus in the animal AD model.^126^ Our results indicated that the expression levels of Wnt7a, β-catenin, LRP6, and FZD4 were significantly higher in all treated groups (by approximately 90%, 196%, 200%, and 220% for Rivas; 175%, 282%, 330%, and 411% for 3a; 63%, 182%, 196%, and 192% for 6a; 159%, 265%, 294%, and 374% for 6c), particularly in treatments 3a and 6c which were higher than those found in the AlCl_3_-treated group (*P* < 0.05), while the expression levels of GSK-3β and BACE1 were significantly lower in all treated groups (by approximately 30% and 51% for Rivas; 56% and 75% for 3a; 29% and 49% for 6a; 53% and 74% for 6c), especially in treatments 3a and 6c, which were lower than those observed in the AlCl_3_-treated group (*P* < 0.05) (Fig. 5A–F). In comparison to AlCl_3_ + Rivas group, administration with 6a and 6c exhibited significantly greater levels of Wnt7a, β-catenin, LRP6, and FZD4 gene expression (*P* < 0.05), while administration with 3a and 6c exhibited significantly lower levels of GSK-3β and BACE1 gene expression (*P* < 0.05), whereas injection with 6a exhibited the same potency as Rivastigmine in the aforementioned genes (Fig. 5A–F). This finding comes in line with that of Ray *et al*.,^127^ who demonstrated that Rivastigmine is a cholinesterase inhibitor, currently used as a symptomatic treatment for mild-to-moderate Alzheimer's disease. In addition, co treatment with Rivastigmine for AlCl_3_-treated rats succeeded in exerting a significant decrease in BACE1, AChE, and IL1B gene expression when compared to AlCl_3_-treated animals.^77^ Also, Pan *et al.*^128^ stated that synthetic and semi-synthetic GSK-3β inhibitors, such as 17 β-carboline-1,2,3-triazole hybrids, exhibit remarkable anti-AD and therapeutic advantages. According to previous investigations, curcumin may improve Wnt/β-catenin signaling by downregulating the expression of the Wnt antagonist DKK1 and upregulating the expression of Wnt proteins and the Wnt co-receptor LRP5/6.^23,129^ Abu-Elfotuh *et al*.^117^ also reported that the combined therapy of sesamol and *L. rhamnosus* dramatically decreased brain amyloid-β, p-tau, GSK-3β, inflammatory, and apoptotic markers while increasing brain free β-catenin and Wnt3a in rats compared to animals intoxicated with AlCl_3_. These results conclude that synthetic compounds 3a and 6c treatment activated the expression of Wnt/β-catenin pathway-related genes, Wnt7a, β-catenin, LRP6, and FZD4, and suppressed the GSK-3β and BACE1 genes, contributing to both investigated compounds potential role as an anti-Alzheimeric effect against AlCl_3_-induced Alzheimer's disease.

#### 2.2.8. Histopathological examination

**Fig. 6:**
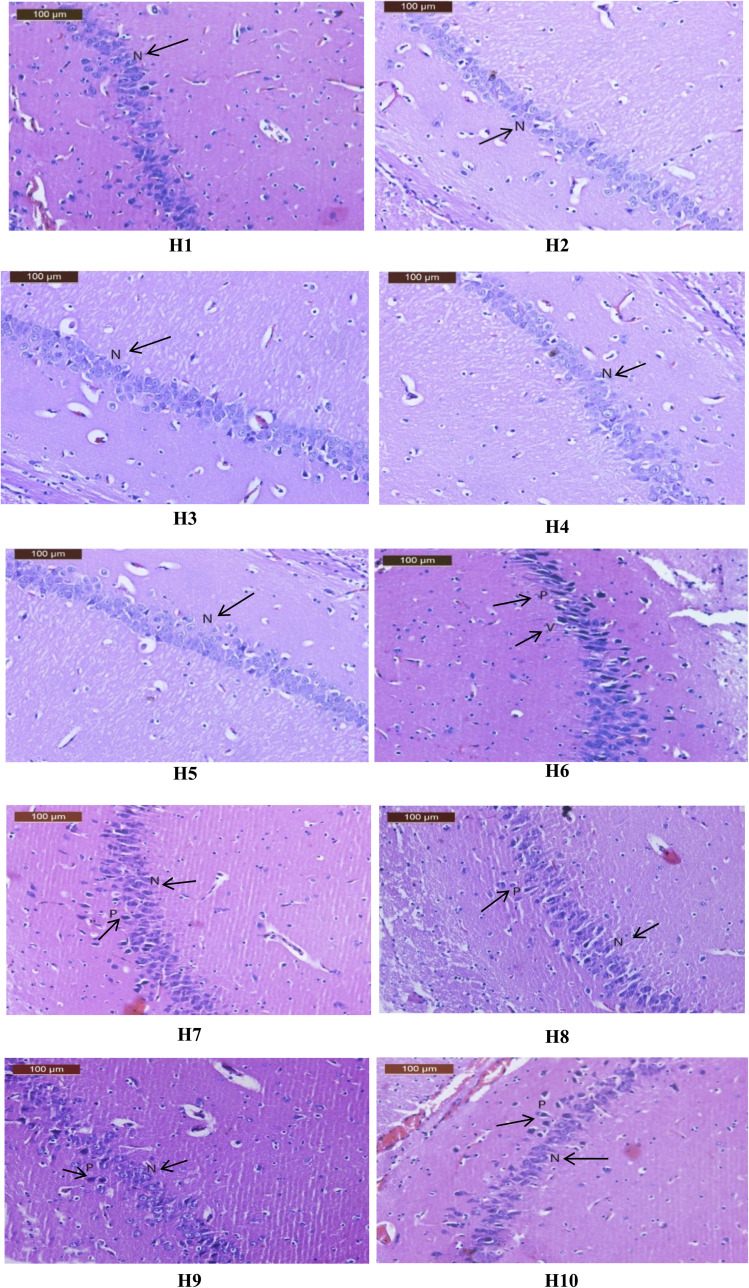
(H1-10) Effects of treatment with Rivastigmine and novel synthesized chemical compounds [3a, 6a, and 6c] on hippocampal tissues in AD-induced group (*n* = 4). (H1-5) A photomicrographs for hippocampi of CTR, Rivas, 3a, 6a, or 6c groups showing well organized pyramidal cells with prominent nuclei (N) (black arrow), (H6) a photomicrograph for hippocampus of Al group showing neurodegeneration in the pyramidal layer with some cell appeared normal nuclei and other shrunken pyramidal cells which have dark eosinophilic cytoplasm and pyknotic nuclei (P) cytoplasmic vacuolation (V) (black arrow), (H7) a photomicrograph for hippocampus of Al + Rivas group showing nearly normal histological structure of preserved pyramidal cells with prominent nuclei (N) and few pyknotic nuclei (P) (black arrow), (H8) a photo-micrograph of hippocampus of Al+3a group showing nearly normal histological structure of preserved pyramidal cells with prominent nuclei (N) and few pyknotic nuclei (P) (black arrow), (H9) a photo-micrograph of hippocampus of Al+6a group showing moderate improvement of pyramidal layer with prominent nuclei (N) and mild pyknotic nuclei (P) (black arrow), (H10) a photomicrograph of hippocampus of Al+6c group showing more or less nearly normal histological structure of preserved pyramidal cells with prominent nuclei (N) and few pyknotic nuclei (P) (black arrow).

Brain sections from the CTR, Rivas, 3a, 6a, or 6c groups showed the hippocampal pyramidal layers illustrated the pyramidal cells with prominent nucleoli (Fig. 6(H1-5)). However, the hippocampal sections of AlCl_3_-exposed group manifested neurodegeneration in the pyramidal layer with some cell appeared with normal nuclei and other shrunken pyramidal cells which have dark eosinophilic cytoplasm, pyknotic nuclei and cytoplasmic vacuolation (black arrow) (Fig. 6(H6)). In contrast, hippocampus of Al + Rivas group displayed nearly normal histological structure of preserved pyramidal cells with prominent nuclei (N) and few pyknotic nuclei (P) (black arrow) (Fig. 6(H7)). Treatment of AD-induced group with 3a compound instigated more or less nearly normal hippocampal histological structure of preserved pyramidal cells with prominent nuclei and few pyknotic nuclei (black arrow) (Fig. 6(H8)). While hippocampus sample of Al+6a group illustrated moderate improvement of pyramidal layer with prominent nuclei and mild pyknotic nuclei (black arrow) (Fig. 6(H9)). Hippocampus samples of Al+6c group showed nearly normal histological structure of preserved pyramidal cells with prominent nuclei and few pyknotic nuclei (black arrow) (Fig. 6(H10)).

**Fig. 7:**
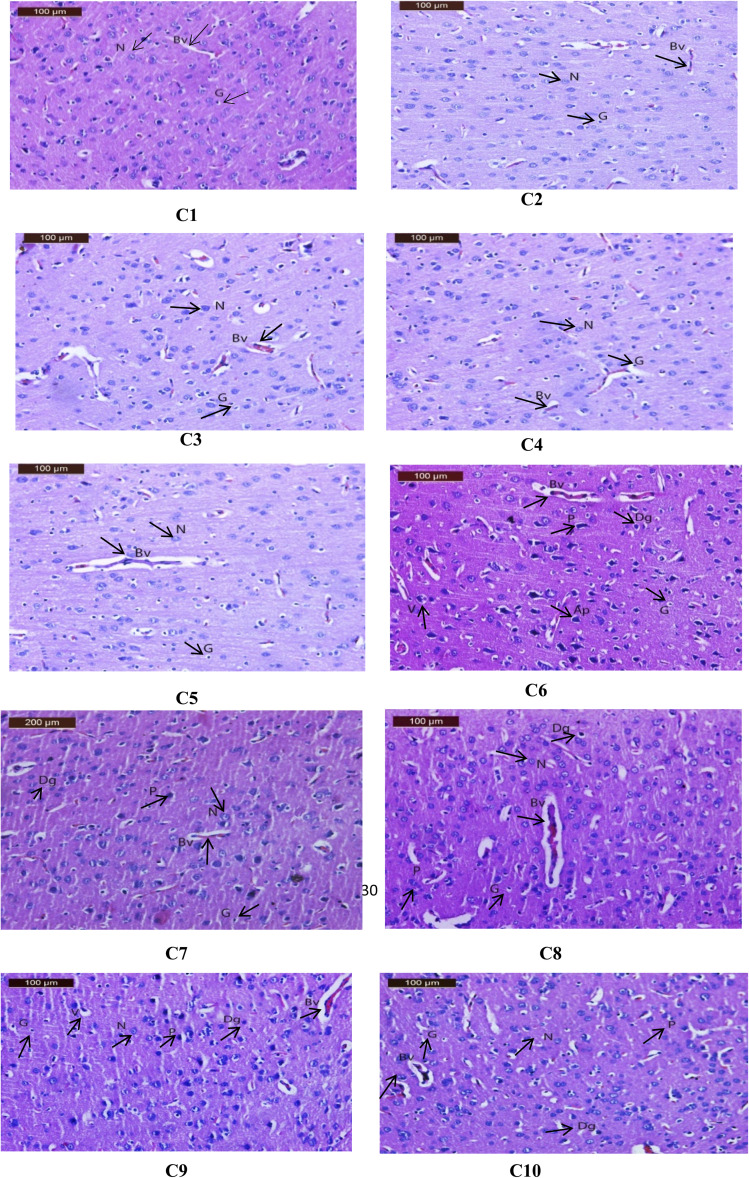
(C1-10) Effects of treatment with Rivastigmine and novel synthesized chemical compounds [3a, 6a, and 6c] on cerebral cortical tissues in AD-induced group (*n* = 4). (C1-5) A photomicrograph of cerebral cortex of CTR, Rivas, 3a, 6a, or 6c groups showing normal histological structure and well-organized brain tissue with normal neurons (N), with glial cells are lightly (G) and with cerebral blood vessels (Bv), (black arrow) (C6) a photomicrograph of cerebral cortex of AlCl_3_ administrated group showing disorganization of cortical layers, degenerated neurocytes with dilated and congested cerebral blood vessels (Bv), slight vacuolated neuropils (V), pyknotic nuclei (P), apoptotic cells (Ap), and glial cells with either many lightly (G) or dark (Dg) stained nuclei, (C7) (black arrow) a photomicrograph of cerebral cortex of Al + Rivason group showing moderate improvement of structure, nearly normal of neuron (N), pyknotic of some nuclei (P), glial cells with either many lightly (G) or dark (Dg) stained nuclei and slight dilated of cerebral blood vessels (Bv|) (black arrow) (C8). A photomicrograph of cerebral cortex of Al+3a group showing almost nearly normal neuronal cells of cortex with few histopathological changes such as minimal pyknotic nuclei (P), and glial cells with either many lightly (G) or few dark (Dg) stained nuclei and slight dilated of cerebral blood vessels (Bv), (black arrow) (C9). A photomicrograph of cerebral cortex of Al+6a group showing moderate improvement of structure, nearly normal of neuron (N), pyknotic of some nuclei (P), pe Exlcular vacuolation (V), glial cells with either many lightly (G) or dark (Dg) stained nuclei and slight dilated of cerebral blood vessels (Bv), (black arrow), (C10) a photomicrograph of cerebral cortex of Al+6c group showing almost nearly normal neuronal cells of cortex with few histopathological changes such as minimal pyknotic nuclei (P), and glial cells with either many lightly (G) or dark (Dg) stained nuclei and slight with normally of cerebral blood vessels (Bv) (black arrow).

Meanwhile, brain cortical sections from the CTR, Rivas,3a, 6a, or 6c groups showed normal histological appearance in the neurons, the blood vessels and glial cells (black arrow) (Fig. 7(C1-5)). Contrarily, sections of the cerebral cortex from the Al group presented disorganization of cortical layers, degenerated neurocytes with dilated and congested cerebral blood vessels, slight vacuolated neuropils, pyknotic nuclei, apoptotic cells, and glial cells with either many lightly or dark stained nuclei (black arrow) (Fig. 7(C6)). The group Al + Rivas displayed moderate improvement of cerebral cortical structure, nearly normal neuron, pyknotic of some nuclei, glial cells with either many lightly or dark stained nuclei and slight dilated cerebral blood vessels (black arrow) (Fig. 7(C7)). Cortical sections of the Al+3a group exhibited nearly normal neuronal cells with few histopathological changes such as minimal pyknotic nuclei, and glial cells with either many lightly or few dark stained nuclei and slight dilated cerebral blood vessels (black arrow) (Fig. 7(C8)). The cortical tissues of Al+6a group showed moderate improvement of structure, nearly normal neuron pyknotic of some nuclei, pe Exlcular vacuolation, glial cells with either many lightly or dark stained nuclei and slight dilated of cerebral blood vessels (black arrow) (Fig. 7(C9)). In cortex sections of Al+6c group disclosed almost nearly normal neuronal cells of cortex with few histopathological changes such as minimal pyknotic nuclei, and glial cells with either many lightly or dark stained nuclei and slight with normal cerebral blood vessels (black arrow) (Fig. 7(C10)).

### 2.3. Molecular docking study

JNK has been shown to play a role in the pathogenesis of Alzheimer's disease with its activation leading to increased Aβ deposition and triggering a positive feedback loop that accelerates AD progression.^15^ JNK consists of two terminal lobes, the N-terminal and C-terminal, connected by a flexible hinge-like region. Notably, the interface between the C and N lobes features a deep cleft that houses the ATP-binding site, which is regarded as an important target for JNK interactions with new drug candidates.^130^ In this study, the protein structure (PDB: 4KKH) of JNK3 co-crystallized with the inhibitor, cyclopropyl[(3*R*)-3-({4-[6-hydroxy-2-(naphthalen-2-yl)-1*H*-benzimidazol-1-yl]pyrimidin-2-yl}amino)piperidin-1-yl]methanone was downloaded from the protein databank to be used for the molecular docking study.^131^ In the co-crystal structure, it was found that the ligand interacted within the binding site *via* two hydrogen bonds with Met149 and one water-bridged hydrogen bond with Lys93, in addition to two H–π interactions with Lys93 and Val196, Table 1. Validation of the docking protocol was carried out through self-docking of the co-crystallized ligand and produced a similar interaction pattern with a low RMSD of 0.5948 Å.

**Table 1:** Docking energy scores (*S*) (kcal mol^−1^) and binding interactions details for the co-crystallized ligand as well as compounds 3a, 6a, and 6c in JNK3 binding site

Compound	Energy score (*S*) kcal mol^−1^	Interactions	Moiety	Residue
3a	−11.8598	Hydrogen bond	N of triazole ring	Met149
		Hydrogen bond	O of sulfonyl group	Asn152
		Water-bridged HB	O of sulfonyl group	Gly71
		H–π interaction	Triazole ring	Gly73
		H–π interaction	Triazole ring	Val196
		H–π interaction	Phenyl ring	Lys93
6a	−11.6482	Hydrogen bond	O of sulfonyl group	Met149
		Water-bridged HB	2 O of phosphate group	Gly71
		Water-bridged HB	2 N of azide group	Lys93
		H–π interaction	Phenyl ring	Ile70
6c	−11.3219	Hydrogen bond	O of sulfonyl group	Met149
		Water-bridged HB	N of phosphanetriamine	Gly71
		Water-bridged HB	N of phosphanetriamine	Lys93
		H–π interaction	Phenyl ring	Ile70
Co-crystallized ligand	−6.9689	Hydrogen bond	N of pyrimidine	Met149
		Hydrogen bond	NH of amine	Met149
		Water-bridged HB	N of benzimidazole	Lys93
		H–π interaction	Pyrimidine ring	Val196
		H–π interaction	Naphthalene ring	Lys93

**Fig. 8:**
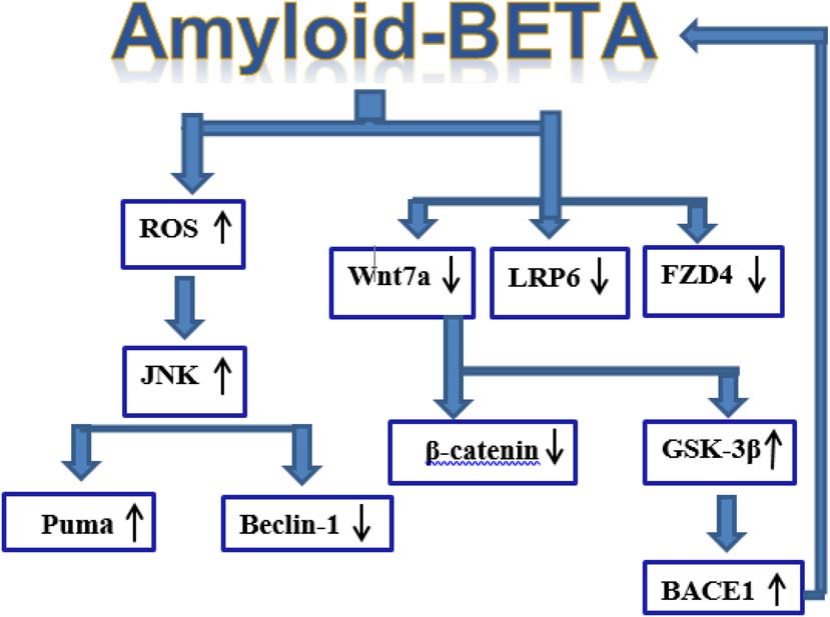
Schematic diagram illustrating effect of amyloid-beta accumulation on apoptosis, autophagy and Wnt/β-catenin signaling pathway in AD-induced rats. Amyloid-β up-regulates Puma and decline Beclin-1 levels *via* activation of the ROS/JNK pathway to promote apoptosis and autophagy. Amyloid-β accumulation leads to inhibition of the Wnt/β-catenin signaling pathway by altering the expression of target genes, including Wnt7a, LRP6, FZD4, and β-catenin, leading to impaired neuronal protection. LRP6 and FZD4 form the receptor complex that mediates Wnt7a signaling, promoting β-catenin stabilization and nuclear translocation to regulate neuronal survival genes. Dysregulation of this pathway results in GSK-3β activation and BACE1 upregulation, further enhancing amyloid-β production and causing neurodegeneration.

The validated docking study was conducted for the most active compounds (3a, 6a, and 6c) within the active site of JNK3 (PDB: 4KKH). All the three compounds formed a hydrogen bond with JNK3 active site key residue, Met149, at the hinge region, highlighting the significance of this hydrogen bond for the activity^132^,^133–135^.

One of the triazole rings of compound 3a was oriented towards the hinge region and hydrogen bonded the backbone nitrogen of Met149 through one of two nitrogen atoms. The sulfonyl group of compound 3a was anchored in the binding site by one hydrogen bond with Asn152 and a water-bridged hydrogen bond with Gly71 using its two sulfonyl oxygens. Moreover, compound 3a exhibited three H–π interactions through its two triazoles and one of the two phenyl rings with three amino acids, Gly73, Val196 and Lys93, respectively.

**Fig. 9:**
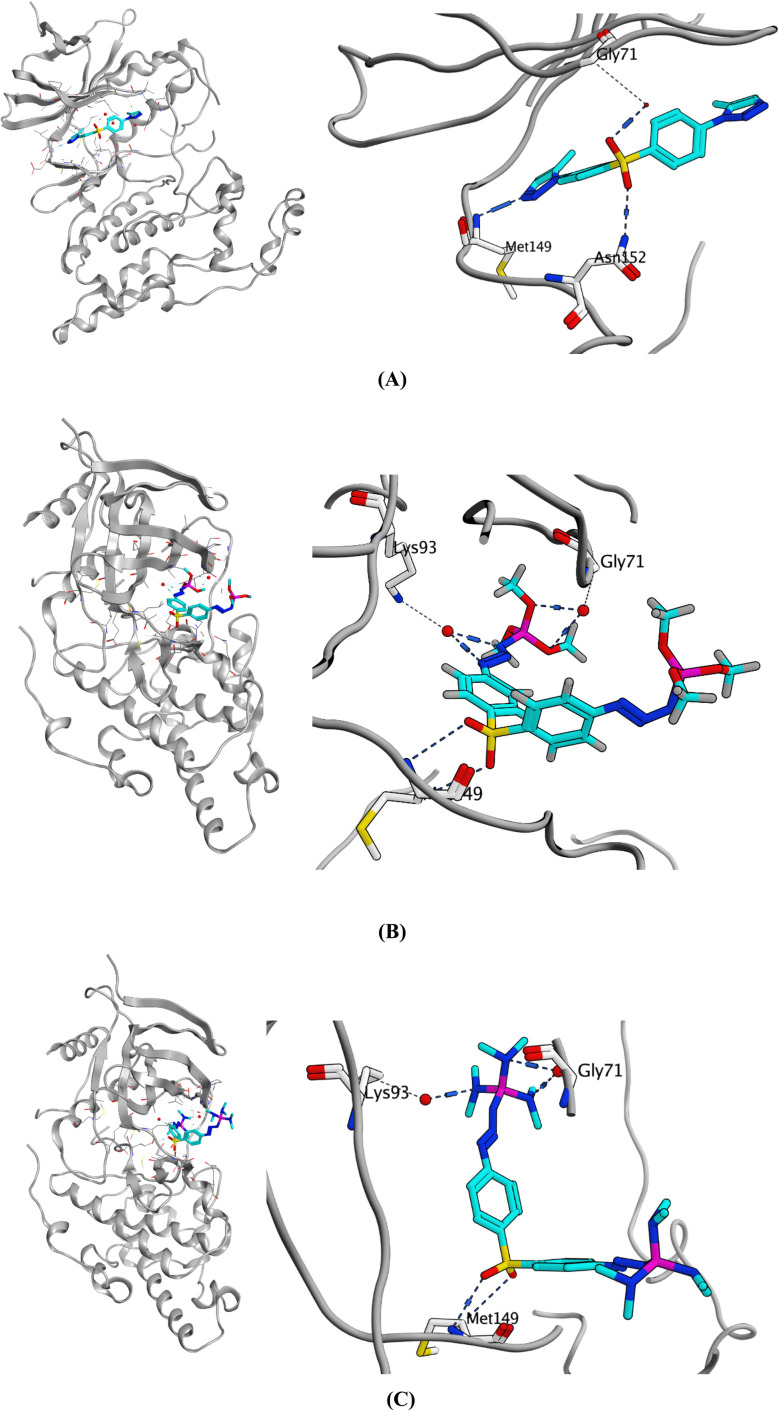
(A–C) Comparative analysis of flexible docking of (A) compound 3a, (B) compound 6a and (C) compound 6c within the binding pocket of JNK3 (protein data bank (PDB) ID: 4KKH).

On the other hand, the sulfonyl groups of compounds 6a and 6c were oriented towards the hinge region, allowing the sulfonyl groups to form one hydrogen bond with the backbone amide NH group of Met149. Both compounds made another water-bridged hydrogen bond with the amino acid residue Gly71 through two oxygen atoms of the phosphate group and the nitrogen atom of the phosphanetriamine group, respectively. Moreover, another water-bridged hydrogen bond was formed between the amino acid Lys93 and two nitrogen atoms of the azide linker in compound 6a and the nitrogen atom of the phosphanetriamine group in compound 6c. In addition, one of the two central phenyl rings of the two compounds was well-positioned allowing sufficient space to establish H–π interactions with the amino acid residue Ile70. The details of the docking results of the three compounds within the JNK3 binding pocket, including orientation and binding interactions in addition to its docking scores, are presented in Table 1 and Fig. 9A–C.

### 2.4. Molecular dynamic simulation study in JNK3 binding site

**Fig. 10:**
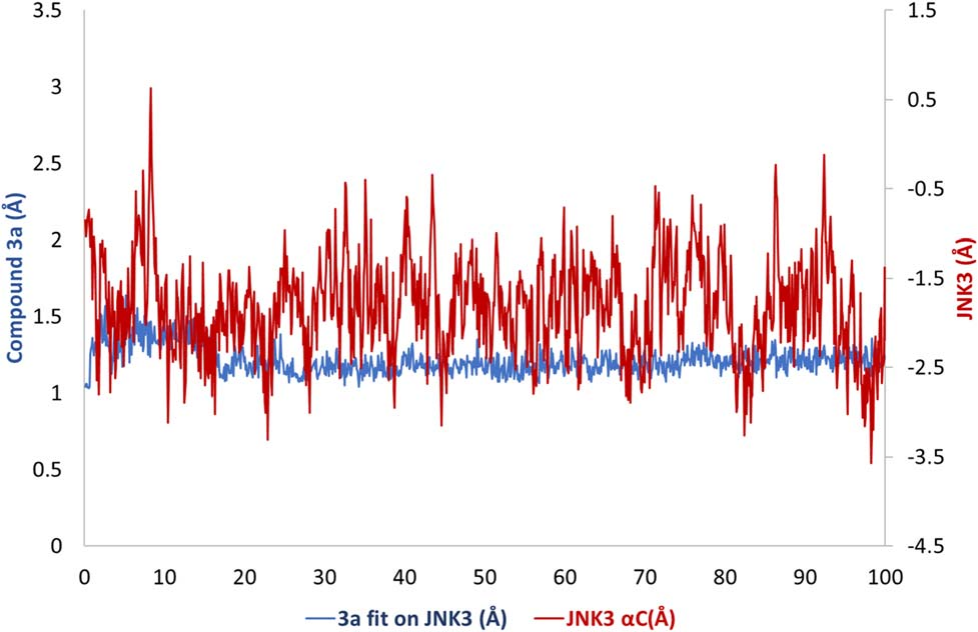
MD trajectory (RMSD *vs.* time) of 3a into JNK3 binding site and Cα atoms of protein.

A molecular dynamics simulation was conducted to investigate the stability of the JNK3–3a complex and to examine how solvation influences its binding energetics. The best-scoring docking pose of compound 3a within the binding pocket was used as the starting structure for a 100 ns molecular dynamics simulation enabling assessment of the predicted ligand–protein binding stability and the interaction persistence over time (Fig. 10).

**Fig. 11:**
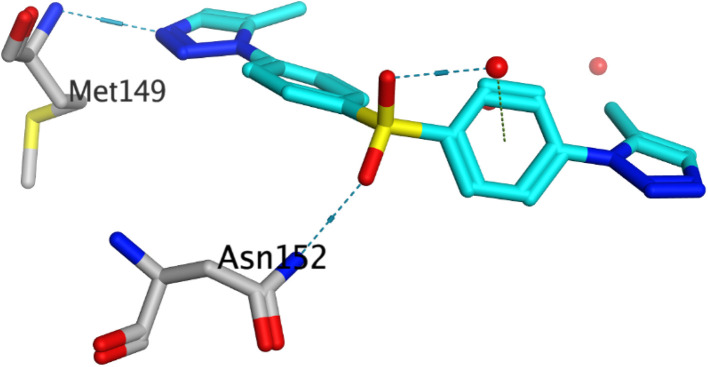
Most persistent conformer of compound 3a into JNK3 active site over a runtime of 100 ns MD simulation (90% persistence).

Within the JNK3 binding site, compound 3a transitions among eleven conformational clusters, showing an average RMSD of 0.63 Å relative to the initial docked pose (Fig. 10A). Notably, one dominant conformer accounts for 90% of the trajectory (Fig. 11). Compound 3a consistently forms three robust hydrogen bonds, with Met149 in the hinge region (78%), Asn152 (88%), and Met146 (61%). In addition, it preserves H–π interactions with Val196 (75%) and Gly73 (18%).

### 2.5. *In silico* prediction of the ADME properties and BBB permeability

Evaluating the pharmacokinetic (PK) properties of small molecules is considered a key feature in most drug development and high-throughput screening processes. Since obtaining PK and toxicity data from *in vivo* or pre-clinical stages is time-consuming and expensive, many efforts have been made to predict ADMET properties *via* computational approaches. Deep-PK is a new deep learning-based PK and toxicity prediction, analysis and optimization platform. Graph neural networks and graph-based signatures are applied as a graph-level feature to yield the best predictive performance across 73 endpoints including 64 ADMET and 9 general properties. In addition, permeability of the blood–brain barrier (BBB) is a critical *in silico* parameter for CNS active drugs that suggests the ability of a chemical to penetrate the BBB into CNS. Hence, we predicted ADME properties and BBB permeability for compounds 3a, 6a, and 6c using Deep-PK web tool (https://biosig.lab.uq.edu.au/deeppk/).^136^

Human oral bioavailability determines the proportion (%) of an orally administered drug that enters the systemic circulation. The results shown in Table 2 indicate that the chance of the oral bioavailability of compounds 3a, 6a and 6c in humans is greater than 50% with high confidence for compound 3a. BBB permeability is predicted by calculating the permeability-surface area (PS) in its logarithmic ratio (log PS) based on *in vivo* in animal models. The distinction of positively (CNSp+) and negatively (CNSp−) classified molecules refers to compounds with log PS values ≥−2 and ≤−3, respectively. As shown in Table 2, all the active compounds were predicted to be BBB penetrable with high confidence. However, brain permeability does not alone dictate efficacy, since many compounds can also be substrates for P-glycoprotein mediated efflux from the brain. The model predicted that all the three compounds are non-P-glycoprotein substrates based on transgenic MDR knockout mice and *in vitro* cell systems.

**Table 2:** Predicted absorption and BBB permeability for compounds 3a, 6a and 6c

Cpd	Absorption		BBB permeability	
	Human oral bioavailability 50%	P-glycoprotein substrate	Log PS	Interpretation
3a	Bioavailable (high confidence)	Non-substrate (low confidence)	−3.07	Penetrable (high confidence)
6a	Bioavailable (medium confidence)	Non-substrate (low confidence)	−2.77	Penetrable (high confidence)
6c	Bioavailable (low confidence)	Non-substrate (low confidence)	−1.17	Penetrable (high confidence)

Cytochrome P450 substrates represent drugs whose pharmacokinetics are largely driven by their metabolism by P450's. The main isoforms responsible for drug metabolism are CYP2D6, CYP2C9 and CYP3A4. As predicted in Table 3, all the tested compounds were assessed to be non-CYP2D6 andCYP2C9substrateswhile compounds 3a and 6c were found to be cytochrome P 3A4 substrates.

**Table 3:** Predicted metabolic cytochromes P enzymes for compounds 3a, 6a and 6c

Cpd	CYP 1A2	CYP 2C19	CYP 2C9	CYP 2D6	CYP 3A4
3a	Substrate (low confidence)	Substrate (low confidence)	Non-substrate (low confidence)	Non-substrate (medium confidence)	Substrate (high confidence)
6a	Substrate (high confidence)	Substrate (low confidence)	Non-substrate (low confidence)	Non-substrate (medium confidence)	Non-substrate (medium confidence)
6c	Substrate (high confidence)	Substrate (high confidence)	Non-substrate (high confidence)	Non-substrate (medium confidence)	Substrate (high confidence)

The plasma protein binding (PPB) measures the non-specific binding of a drug to plasma proteins, which can affect the amount of free drug in the body. This is an important pharmacokinetic property because only the unbound fraction of a drug is typically available to exert pharmacological effects or be metabolized and eliminated from the body. Therefore, it directly influences the drug's potency, efficacy, and potential for adverse effects. The plasma protein binding % of the three compounds are predicted to be lower than 90%, which indicates their availability for therapeutic effect, ranging from 18.19 to 76.87% value with compound 6c having the lowest value, Table 4.

**Table 4:** Predicted distribution and excretion properties for compounds 3a, 6a and 6c

Cpd	Distribution		Excretion	
	Plasma protein binding %	Log VDss	Total clearance (mL min^−1^ kg^−1^)	Half-life (*t*_1/2_) (h)
3a	76.87	0.28	0.62	Half-life < 3 h (medium confidence)
6a	45.83	0.9	1.27	Half-life < 3 h (high confidence)
6c	18.19	1.07	2.59	Half-life < 3 h (high confidence)

The steady state volume of distribution (VDss) is the theoretical volume that the total dose of a drug would need to be uniformly distributed to give the same concentration as in blood plasma. The higher the VD is, the more of a drug is distributed in tissue rather than plasma. VDss is considered low if below 0.71 L kg^−1^ (log VDss < −0.15) and high if above 2.81 L kg^−1^ (log VDss > 0.45). The log VDss values of compounds 6a and 6c, as shown in Table 4, are considered high while that of compound 3a is considered moderate.

Deep-PK estimates the total clearance (mL min^−1^ kg^−1^) of the compound which is primarily a combination of hepatic clearance and renal clearance. It is important for determining dosing rates to achieve steady-state concentrations. The predicted total clearance is presented in Table 4, showing that compound 6c has the highest clearance rate. For half-life (*t*_1/2_), deep-PK predictor only determines whether a given compound is likely to have an elimination half-life that is greater than or equal to 3 hours or less than 3 hours. All the compounds were found to have half-life (*t*_1/2_) less than 3 hours.

## 3. Conclusion

The present study successfully synthesized and characterized novel triazole and phosphazine derivatives, which demonstrated significant neuroprotective activity in an AlCl_3_-induced AD rat model. Compounds 3a and 6c notably outperformed Rivastigmine® in improving memory and cognitive performance, reducing Aβ_1–42_ generation, oxidative stress, JNK and Puma activation, while enhancing Beclin-1 expression, downregulating GSK-3β and BACE1 gene expression levels and upregulating Wnt/β-catenin signaling-related genes, including Wnt7a, β-catenin, LRP6, and FZD4 (Fig. 8). These findings were supported by molecular docking and dynamics analysis, confirming strong interactions with JNK3, a key mediator in AD progression. Additionally, compounds 3a, 6a, and 6c were predicted to exhibit favorable oral bioavailability, with the ability to cross the blood–brain barrier and avoid recognition as substrates by P-glycoprotein. These compounds are anticipated to remain available in the bloodstream for therapeutic action, showing predicted plasma protein binding below 90%, moderate to high steady-state volume of distribution, and no substrate affinity for cytochrome P450 isoforms 2C9 and 2D6. Taken together, the data highlight compound 3a, in particular, as a promising multifunctional lead for further development as an anti-Alzheimer's agent targeting multiple pathological pathways.

## 4. Experimental

### 4.1. Chemistry

All chemicals were supplied by either Fluka or Aldrich chemical companies and were used without further purification. For more information, see SI Appendix A. 4,4′-Sulfonylbis(azidobenzene) (1) was prepared according to a reported method.^137^

#### 4.1.1 General procedure for the reaction of 4,4′-sulfonylbis(azidobenzene) (1) with Wittig reagents (2a,b)

To a dry, inert atmosphere flask (argon), 4,4′-sulfonylbis(azidobenzene) (1) (1.0 mmol) was dissolved in dry toluene (15 mL) under stirring. The reaction mixture was cooled to 0 °C in an ice bath. A pre-generated Wittig reagent (2a or 2b) (2.2 mmol), prepared by treating the corresponding phosphonium salt with a strong base such as sodium carbonate (2.2 mmol), was added dropwise to the reaction mixture under argon atmosphere.

The reaction was stirred for 2 hours. The progress of the reaction was monitored by thin-layer chromatography (TLC). After completion, the crude product was evaporated under reduced pressure and was purified by column chromatography on silica gel using an appropriate eluent (petroleum ether (60 : 80 °C)/ethyl acetate gradient) to afford the desired 3a,b derivative as a solid.

#### 4.1.2 1,1′-(Sulfonylbis(4,1-phenylene))bis(5-methyl-1*H*-1,2,3-triazole) (3a)

Eluent: petroleum ether (60 : 80 °C)/ethylacetate (40/60 v/v). Compound 3a was separated as brown crystals, mp 233–235 °C, yield 65%. IR (KBr), cm^−1^, *ν* 3200 (C–N), 2950 (C–H), 1550 (C=N), 1300 (C=C), 1200 (S=O), 1137 (S=O). ^1^H NMR (500 MHz, DMSO-*d*_6_) *δ* 8.28 (d, *J* = 8.6 Hz, 2H, CH_aromatic_), 7.95 (d, *J* = 8.6 Hz, 2H, CH_aromatic_), 7.74 (s, 1H, CH_triazole_), 2.38 (s, 3H, Me). ^13^C NMR (126 MHz, DMSO-*d*_6_) *δ* 140.6 (triazole C), 140.0 (aromatic C–N), 134.4 (aromatic C–S), 133.8 (aromatic CH), 129.1 (aromatic CH), 125.4 (CH triazole), 8.8 (Me). MS (*m*/*z* 379, 5%).

#### 4.1.3 1,1′-(Sulfonylbis(4,1-phenylene))bis(5-phenyl-1*H*-1,2,3-triazole) (3b)

Eluent : petroleum ether (60 : 80 °C)/ethyl acetate (30 : 70, v/v). Compound 3b was separated as colorless crystals, mp 215–216 °C, yield 50%. IR (KBr), cm^−1^, *ν* 3150 (C–N), 2950 (C–H), 1560 (C=N), 1350 (C=C), 1150 (S=O), 1137 (S=O). ^1^H NMR (500 MHz, DMSO-*d*_6_) *δ* 8.16 (s, 1H, CH_triazole_), 7.68 (d, *J* = 8.9 Hz, 2H, CH_aromatic_), 7.43 (d, *J* = 8.9 Hz, 2H, CH_aromatic_), 7.40 (t, *J* = 8.9 Hz, 3H, CH_aromatic_), 7.30 (d, *J* = 8.2 Hz, 2H, CH_aromatic_). ^13^C NMR (126 MHz, DMSO-*d*_6_) *δ* 141.1 (triazole C), 140.4 (aromatic C–N), 138.1 (aromatic CH), 133.9 (aromatic C–S), 129.7 (aromatic CH), 129.3 (aromatic CH), 128.9 (aromatic CH), 128.3 (aromatic CH), 126.7 (aromatic CH), 126.0 (CH triazole). MS (*m*/*z* 504, 15%).

#### 4.1.4 General procedure for the reaction of 4,4′-Sulfonylbis(azidobenzene) (1) with phosphorus reagents (4a–d)

In a dry, nitrogen-purged 100 mL round-bottom flask, 4,4′-sulfonylbis(azidobenzene) (1) (1.0 mmol) was dissolved in dry toluene (20 mL) under an inert atmosphere (argon). The solution was stirred at room temperature for 10 minutes.

To this stirred solution, trialkylphosphite (4a: trimethylphosphite or 4b: triisopropylphosphite) or tridialkylaminophosphine (4c: hexamethylphosphoroustriamide or 4d: hexaethylphosphoroustriamide) was added dropwise (2.2 mmol, 2.2 equiv.). The reaction mixture was stirred for 30 min, during which evolution of nitrogen gas was observed, indicating progress of the Staudinger and subsequent rearrangement processes.

Upon cooling to room temperature, the solvent was evaporated under reduced pressure. The residue was purified by silica gel column chromatography using a gradient of hexane/ethyl acetate to isolate products 5a–5d, and 6a–6d.

#### 4.1.5 Trimethyl (4-((4-(((trimethoxy-λ^5^-phosphaneylidene)methylene)amino)phenyl)sulfonyl)phenyl)phosphorimidate (5a)

Eluent : ethyl acetate (60 : 80 °C)/ethyl alcohol (85 : 15, v/v). Compound 5a was separated as colorless crystals, mp 213–215 °C, yield 50%. IR (KBr), cm^−1^, *ν* 3100 (P–N–C), 1300 (C=C), 1250 (P=N), 1075 (SO_2_). ^1^H NMR (500 MHz, CDCl_3_) *δ* 7.62 (d, *J* = 8.9 Hz, 2H, CH_aromatic_), 7.11 (d, 2H, *J* = 8.9 Hz, CH_aromatic_), 3.62 (m, 9H, OMe). ^13^C NMR (126 MHz, CDCl_3_) *δ* 145.6 (aromatic C–N), 133.5 (aromatic C–S), 128.5 (aromatic CH), 117.2 (aromatic CH), 53.0–53.1 (OMe). ^31^P NMR (121 MHz, CDCl_3_) *δ* 20.9 ppm. MS (*m*/*z* 491, 5%).

#### 4.1.6 Triisopropyl (4-((4-(((triisopropoxy-λ^5^-phosphaneylidene)methylene)amino)phenyl)sulfonyl)phenyl)phosphorimidate (5b)

Eluent : petroleum ether (60 : 80 °C)/ethyl acetate (90 : 10, v/v). Compound 5b was separated as colorless crystals, mp 137–139 °C, yield 50%. IR (KBr), cm^−1^, *ν* 3050 (P–N–C), 1300 (C=C), 1250 (P=N), 1075 (SO_2_). ^1^H NMR (500 MHz, CDCl_3_) *δ* 7.77 (d, *J* = 7.5 Hz, 2H, CH_aromatic_), 7.03 (d, *J* = 7.5 Hz, 2H, CH_aromatic_), 4.67 (m, 3H, CH), 1.74 (s, 3H, Me), 1.37 (d, *J* = 5.8 Hz, 6H, 2 Me), 1.23 (dd, *J* = 14.2, 6.1 Hz, 9H, 3 Me). ^13^C NMR (126 MHz, CDCl_3_) *δ* 131.2 (aromatic C–N), 128.49 (aromatic C–S), 122.9 (d, *J* = 19.2 Hz, CH_aromatic_), 122.8 (CH_aromatic_), 72.8 (d, ^2^*J*_C–P_ = 32.6 Hz, P–O–CH), 23.7 (d, ^4^*J*_C–P_ = 5.2 Hz, P–O–CH–*C*H_3_). ^31^P NMR (121 MHz, CDCl_3_) *δ* 21.98 ppm. MS (*m*/*z* 660, 3%).

#### 4.1.7 *N*,*N*'–(Sulfonylbis(4,1-phenylene))bis(1,1,1-tri(dimethylamino)–λ^5^-phosphanimine) (5c)

Eluent : ethyl acetate (60 : 80 °C)/ethyl alcohol (90/10, v/v). Compound 5c was separated as yellow crystals, mp 165–166 °C, yield 80%. IR (KBr), cm^−1^, *ν* 3150 (P–N–C), 1600 (C=C), 1250 (P=N), 1100 (SO_2_). ^1^H NMR (500 MHz, CDCl_3_) *δ* 7.85 (d, *J* = 7.5 Hz, 2H, CH_aromatic_), 7.54 (d, *J* = 7.5 Hz, 2H, CH_aromatic_), 2.62–3.01 (m, 18H, 6Me). ^13^C NMR (126 MHz, CDCl_3_) *δ* 155.4 (aromatic C–N), 136.8 (aromatic C–S), 127.6 (s, CH_aromatic_), 120.2 (CH_aromatic_), 36.3 (m, P–N(Me)_2_). ^31^P NMR (121 MHz, CDCl_3_) *δ* 21.55 ppm. MS (*m*/*z* 570, 45%).

#### 4.1.8 *N*,*N*'–(Sulfonylbis(4,1-phenylene))bis(1,1,1-tri(diethylamino)-λ^5^-phosphanimine) (5d)

Eluent : ethyl acetate (60 : 80 °C)/ethyl alcohol (80 : 20, v/v). Compound 5d was separated as yellow crystals, mp 158–159 °C, yield 65%. IR (KBr), cm^−1^, *ν* 3150 (P–N–C), 1600 (C=C), 1250 (P=N), 1100 (SO_2_). ^1^H NMR (500 MHz, CDCl_3_) *δ* 7.81 (d, *J* = 7.5 Hz, 2H, CH_aromatic_), 7.52 (d, *J* = 7.5 Hz, 2H, CH_aromatic_), 3.15–3.47 (m, 12H, 6CH_2_), 1.15–1.29 (m, 18H, 6Me). ^13^C NMR (126 MHz, CDCl_3_) *δ* 155.4 (aromatic C–N), 132.8 (s, aromatic C–S), 128.3 (s, CH_aromatic_), 120.3 (s, CH_aromatic_), 39.7 (m, P–N–*CH*_*2*_–CH_3_), 13.5 (m, P–N–CH_2_–*CH*_*3*_). ^31^P NMR (121 MHz, CDCl_3_) *δ* 22.64 ppm. MS (*m*/*z* 792, 8%).

#### 4.1.9 Hexamethyl((sulfonylbis(4,1-phenylene))bis(triaz-2-en-3-yl-1-ylidene))bis(phosphate) (6a)

Eluent : ethyl acetate (60 : 80 °C)/ethyl alcohol (95 : 5, v/v). Compound 6a was separated as page crystals, mp 165–66 °C, yield 10% %.IR (KBr), cm^−1^, *ν* 3150 (P–N–C), 1600 (C=C), 1287 (C–P–N), 1250 (P=N), 1100 (SO_2_),1080 (=N–N). ^1^H NMR (500 MHz, CDCl_3_) *δ* 7.70 (d, *J* = 8.9 Hz, 2H, CH_aromatic_), 7.12 (d, 2H, *J* = 8.9 Hz, CH_aromatic_), 6.59 (d, 2H, *J* = 8.9 Hz, CH_aromatic_), 6.10 (d, 2H, *J* = 8.9 Hz, CH_aromatic_), 3.62 (m, 9H, OMe). ^13^C NMR (126 MHz, CDCl_3_) *δ* 135.4 (aromatic C–N), 131.0 (aromatic C–S), 128.2 (aromatic CH), 129.1 (aromatic CH), 117.1 (aromatic CH), 113.0 (aromatic CH), 53.2 (OMe). ^31^P NMR (121 MHz, CDCl_3_) *δ* 30.4 ppm. MS (*m*/*z* 548, >5%).

#### 4.1.10 Hexaisopropyl((sulfonylbis(4,1-phenylene))bis(triaz-2-en-3-yl-1-ylidene))bis(phosphate) (6b)

Eluent : petroleum ether (60 : 80 °C)/ethyl acetate (70 : 30, v/v). Compound 6b was separated as colorless crystals, mp 123–125 °C, yield35%. IR (KBr), cm^−1^, *ν* 3130 (P–N–C), 1650 (C=C), 1280 (C–P–N), 1180 (=N–N), 1100 (SO_2_). ^1^H NMR (500 MHz, CDCl_3_) *δ* 7.77 (d, *J* = 7.5 Hz, 2H, CH_aromatic_), 7.03 (d, *J* = 7.5 Hz, 2H, CH_aromatic_), 4.67 (m, 3H, CH), 1.74 (s, 3H, Me), 1.37 (d, *J* = 5.8 Hz, 6H, 2 Me), 1.23 (dd, *J* = 14.2, 6.1 Hz, 9H, 3 Me). ^13^C NMR (126 MHz, CDCl_3_) *δ* 131.2 (aromatic C–N), 128.49 (aromatic C–S), 122.9 (d, *J* = 19.2 Hz, CH_aromatic_), 122.8 (CH_aromatic_), 72.8 (d, ^2^*J*_C–P_ = 32.6 Hz, P–O–CH), 23.7 (d,^4^*J*_C–P_ = 5.2 Hz, P–O–CH–*CH*_*3*_). ^31^P NMR (121 MHz, DMSO) *δ* 31.1 ppm. MS (*m*/*z* 714, >5%).

#### 4.1.11 1,1′-((Sulfonylbis(4,1-phenylene))bis(triaz-2-en-3-yl-1-ylidene))bis(*N*,*N*,*N*′,*N*′,*N*″,*N*″-hexamethyl-l5-phosphanetriamine) (6c)

Eluent : ethyl acetate (60 : 80 °C)/ethyl alcohol (80 : 20, v/v). Compound 6c was separated as yellow crystals, mp 91–93 °C, yield 10%. ^1^H NMR (500 MHz, CDCl_3_) *δ* 7.83 (d, *J* = 7.5 Hz, 2H, CH_aromatic_), 7.51 (d, *J* = 7.5 Hz, 2H, CH_aromatic_), 2.60–2.98 (m, 18H, 6Me). ^13^C NMR (126 MHz, CDCl_3_) *δ* 134.4 (aromatic C–N), 131.8 (aromatic C–S), 125.9 (s, CH_aromatic_), 120.3 (CH_aromatic_), 36.1 (m, P–N(Me)_2_). ^31^P NMR (121 MHz, DMSO) *δ* 30.7 ppm.

#### 4.1.12 1,1′-((Sulfonylbis(4,1-phenylene))bis(triaz-2-en-3-yl-1-ylidene))bis(*N*,*N*,*N*′,*N*′,*N*″,*N*″-hexaethyl-l5-phosphanetriamine) (6d)

Eluent : ethyl acetate (60 : 80 °C)/ethyl alcohol (90 : 10, v/v). Compound 6d was separated as yellow crystals, mp 90–92 °C, yield 20%. IR (KBr), cm^−1^, *ν* 3150 (P–N–C), 1600 (C=C), 1287 (C–P–N), 1250 (P=N), 1100 (SO_2_), 1080 (=N–N). ^1^H NMR (500 MHz, CDCl_3_) *δ* 7.83 (d, *J* = 7.5 Hz, 2H, CH_aromatic_), 7.55 (d, *J* = 7.5 Hz, 2H, CH_aromatic_), 3.0–3.46 (m, 12H, 6CH_2_), 1.12–1.20 (m, 18H, 6Me). ^13^C NMR (126 MHz, CDCl_3_) *δ* 136.4 (aromatic C–N), 132.8 (s, aromatic C–S), 128.1 (s, CH_aromatic_), 120.1 (s, CH_aromatic_), 39.4 (m, P–N–*CH*_*2*_–CH_3_), 13.1 (m, P–N–CH_2_–*CH*_*3*_). ^31^P NMR (121 MHz, DMSO) *δ* 30.9 ppm, MS (*m*/*z* 796, 7%).

### 4.2. Biological inspections

#### 4.2.1 Reagents and drugs

Aluminum Chloride was acquired from Merc Company, Germany. Exelon® capsules (Rivastigmine; 1.5 MG) manufactured by Novartis Company, Switzerland was purchased from a local pharmacy. Distilled water was used to reconstitute each; however, synthesized chemical compounds dissolved in DMSO 5%. Unless otherwise noted, every additional chemical used for analysis satisfied the worldwide quality requirements.

#### 4.2.2 Animals housing

The Animal House Colony of the National Research Centre, Giza, Egypt, provided seventy adult fit male *Wister* rats [230–250 g]. They are accommodated in pathogen-free environments and standard laboratory conditions [room temperature (22 °C ± 2 °C), consistent light–dark cycle (12 : 12 h), and humidity (65% ± 5%)]. Animals housed for seven days prior to the experimentation in a good-ventilated room for acclimating with unconstrained access to water and diet. All animals procedures were performed in accordance with the guidelines for care and use of laboratory animals of National Research Centre, Egypt and approved by the animal ethics committee of Medical Research Ethical Committee with approval number [13010102-1].

#### 4.2.3 Animal grouping and treatment procedures

In the current research, the therapeutic impacts of various novel synthesized chemical compounds on the attenuation of AD progression were evaluated in AlCl_3_-intoxicated rat model. Animals were blindly segregated into ten distinct groups (*n* = 7), the normal control group (CTR), normal Rivastigmine-treated group (Rivas), normal 3a-treated group (3a), normal 6a-treated group (6a), normal 6c-treated group (6c), AlCl_3_-treated group (Al), AlCl_3_ and Rivastigmine-treated group (Al + Rivas), AlCl_3_ and 3a-treated group (Al+3a), AlCl_3_ and 6a-treated group (Al+6a), AlCl_3_ and 6c-treated group (Al+6c). Animal grouping is described in Table 5. The rats in all groups received their corresponding treatment daily. Finally, the rats were subjected to several behavioral investigations. Consequently, the rats sacrificed, and brain tissue samples were collected for further studies.

**Table 5:** Experimental strategy for AD induction and treatment in rat model

Groups	Groups descriptions	Number of animals	References
CTR	Normal healthy rats injected daily with DMSO 5% i.p. for 45 days	7	
Rivas	Normal rats gavage daily with Rivastigmine [3 mg per kg body wt] for 45 days	7	138
3a	Normal rats injected daily with 3a compound i.p. [10 mg per kg body wt] for 45 days	7	139
6a	Normal rats injected daily with 6a compound i.p. [10 mg per kg body wt] for 45 days	7	139
6c	Normal rats injected daily with 6c compound i.p. [10 mg per kg body wt] for 45 days	7	139
Al	Normal rats intoxicated with AlCl_3_ [100 mg per kg body wt] for four months for AD induction by oral gastric tube	7	21
Al + Rivas	Rats received AlCl_3_ and consequently treated with Rivastigmine for 45 days	7	138
Al+3a	Rats received AlCl_3_ then injected with 3a compound for 45 days	7	139
Al+6a	Rats received AlCl_3_ then injected with 6a compound for 45 days	7	139
Al+6c	Rats received AlCl_3_ then injected with 6c compound for 45 days	7	139

#### 4.2.4 Neurobehavioral studies

##### 4.2.4.1 Assessment of Morris water maze test

Morris water maze test^140^ was applied for spatial memory testing. The water maze consisted of a plastic circular water pool (150 cm diameter and 60 cm height), filled with 24 ± 2 °C water up to 45 cm height and made opaque using a nontoxic colored dye. The pool was divided into virtually four equal quadrants (N, S, E, and W) and an invisible platform (10 × 10 cm) was submerged 2–3 cm below the water at one quadrant which remained in the same quadrant throughout the experiment. The test was conducted for 5 consecutive days where the acquisition training phase was done for 4 days and the last day for the probe testing. During the training phase, rats were placed in the pool at three different starting places daily rather than the fourth where the platform was located (target quadrant) and each animal had 60 seconds to find the platform and was allowed to stand on it for 20 seconds. If the animal failed to locate the platform within one minute the observer directed it to swim there and gave it 20 seconds to remain there. Three training attempts were carried out for each tested animal daily beginning from three distinct starting places. The water was plain for the first 2 days of the training phase, meanwhile, a nontoxic colored dye was used to make the water opaque for the second 2 days of the acquisition. On the fifth day of the test (probe phase), the platform was withdrawn, and each rat was positioned in the quadrant that faced the target quadrant. A digital camera was used to record the probing trial. The escape latency (in sec) (The time spent reaching the platform) during acquisition phase and probe phase was recorded.^47,62^

##### 4.2.4.2 Evaluation of novel object recognition test

Based on the rodents' natural preference for novelty, a 2 day NOR test was performed to evaluate short term recognition memory in rats. This task was conducted in a square arena measuring 50 × 50 cm with a 70 cm height and a video camera was suspended 100 cm above for recording the exploratory behavior of rats. The test had two stages (the familiarization phase and the test phase). On the first day (familiarization phase), two familiar small objects were placed in the square arena equally spaced from each other and the walls of the test box. The animal was allowed to explore the objects for approximately 10–15 minutes freely. After 24 hours, the second phase (testing phase) was conducted again, during which each rat was exposed for five minutes to a novel object and one familiar object. The test arena and object were disinfected with 70% alcohol between trials. When the rat touched or pointed its nose, mouth or paw at the object, it was considered to be engaging in exploratory behavior. The time (sec) spent exploring each object is recorded.

The discrimination index (DI), discrimination ratio (DR), and difference score (DS) were calculated as follows:

DI = (time spent exploring the novel object – time spent exploring the familiar object)/total exploration time.^141,142^

DR = (time spent exploring the novel object/total exploration time) × 100.

DS = time spent exploring the novel object-time spent exploring the familiar object.

A higher exploration time for the novel object and discrimination index greater than 0.5 indicated successful object recognition and memory function.

##### 4.2.4.3 Estimation of Y maze test

The Y maze test was performed to assess the spatial working memory. To perform this test, a y-shaped apparatus consisted of three similar dark-colored wooden arms (labeled A, B and C). The measurement of each arm was 40 cm long, 40 cm high, and 18 cm wide. We tested each rat for eight minutes and it was allowed to move freely throughout the Y maze's three arms. A complete arm entrance occurs when the rat uses all of his limbs to enter the maze's arm. Good spatial memory is demonstrated by a high proportion of three arms alternation. Between sessions, 70 percent alcohol was used to wipe the maze. A digital camera was used to record animal's behavior, including spontaneous alternation (ie the entry into the three different arms in concession, ABC, CBA, BAC…), alternate arm returns (AAR), same arm returns (SAR), the total arm entries (TAE) were calculated.

Spontaneous alternation percentage (SAP) = [(number of alternations)/(TAE − 2)] × 100.^62,143^

#### 4.2.5 Brain tissue preparation

Finally, after the animal's cervical dislocation, brain samples of each group were gathered and homogenized in an ice-cold phosphate buffer saline [PBS, pH 7.4, 0.1 M] producing 20% (w/v) homogenate then centrifuged for 10 min at 10 000 rpm and 4 °C, yielding a clear supernatant suitable for biochemical assays. Using a total protein colorimetric assay kit with the bicinchoninic acid (BCA) method (Thermo Fisher Scientific, USA) the total protein content was determined and the various biochemical parameters' concentrations expressed in mg^−1^ protein. Hippocampi were isolated then preserved at −80 °C for gene expression investigations. For histopathological examination, brain tissues were fixed in a 10% neutral buffered formalin solution.

#### 4.2.6 Biochemical assessment

All estimated biochemical markers were detected in brain homogenate of six rats from each group using rat ELISA assay kits, according to the manufacturers' procedure provided with the kits. Absorbance measured at 450 nm.

Brain amount of amyloid beta1-42 peptide (MyBioSource, USA) and reactive oxygen species (Sunlong Biotech Co., CHINA) were detected and expressed as pg mg^−1^ protein. Besides, C-Jun N-terminal kinases (JNK; Sunlong Biotech Co., CHINA), p53 upregulated modulator of apoptosis (Puma; Cloud-Clone Corp, USA), and Beclin-1 (MyBioSource, USA) levels were quantified and reported as ng mg^−1^ protein.

#### 4.2.7 Quantitative real-time PCR analysis (qRT-PCR)

RNA was extracted from hippocampus tissues of sex rats from each group using the RNeasymini-Kit (Qiagen, Hilden, Germany) (Cat. no.: 74104). A NanoDrop 2000 spectrophotometer (Thermo Fisher Scientific, USA) was then used to measure the concentration and purity of the total extracted RNA. The Revert Aid First Strand cDNA Synthesis Kit (Thermo Fisher Scientific, Waltham, MA, USA) (Cat. no.: K1621) was used to convert the RNA from each treatment to first-strand cDNA in accordance with the manufacturer's instructions. Table 6 lists primer sequences. Using the Maxima SYBR Green qPCR Master Mix (2×) (Thermo Fisher Scientific, Waltham, MA, USA) (Cat. no.: K0221), the expression levels of the genes Wnt7a, β-catenin, GSK-3β, LRP6, FZD4, and BACE1 were normalized with respect to the β-actin transcript and computed using the 2^−ΔΔ*CT*^ procedure.^144^ 40 cycles of amplification were performed using the following reaction conditions: 95 °C for 10 min, 95 °C for 15 s, 55 °C for 30 s, and 72 °C for 30 s. Gene expression was measured using a DNA Technology Detecting Thermocycler DT Lite 4S1 (Russia).

**Table 6:** Primer sequences used for qRT-PCR analysis

Gene	Forward primer (5′–3′)	Reverse primer (5′–3′)
β-Actin	CACGTGGGCCGCTCTAGGCACCAA	CTCTTTGATGTCACGCACGATTTC
Wnt7a	ACACTGCCACAATTCCGAGA	ATGGACGGCCTCGTTGTATT
β-Catenin	ATGGAGCCGGACAGAAAAGC	CTTGCCACTCAGGGAAGGA
GSK-3β	CTTTGGAAGTGCAAAGCAG	CCAACTGATCCACACCAC
LRP6	TACTCTGTAACGGGCTGGTG	ACAAGCTTGACCGGAGACAA
FZD4	GCTACAACGTGACCAAGATG	GAATTGCTTCCCACGGAGT
BACE1	TCACCAATCAGTCCTTCCGC	GGGCTCGATCAAAGACCACA

#### 4.2.8 Histopathological examination

After sacrificing control and experimental animals, the cerebral cortex and hippocampi were sequestered from four rats per group and fixed immediately in 10% neutral buffered formalin. Paraffin-embedded blocks were organized for both tissues. After sectioning to 5 µm thickness using microtome, they were stained with hematoxylin–eosin and finally examined with light microscope.^145^

#### 4.2.9 Statistical analysis

All findings in this report were defined as mean ± SEM of the mean. Concerning the biochemical outcomes, the Statistical Package for Social Science (SPSS), version 25, was useful in analyzing the data using one-way analysis of variance (ANOVA), then least significant difference (LSD) was utilized to compare significance through the groups. Besides, for molecular data one-way ANOVA was used to establish if there was a statistically significant difference across the groups; the Tukey–Kramer multiple comparison test was then used. GraphPad Prism version 8 was utilized for data analysis. When the *P* value was less than 0.05 for each test, the difference was assumed significant.

### 4.3. Molecular docking study

For carrying out the docking studies, Molecular Operating Environment software (MOE 2024.06) was utilized. The crystal structure of JNK3 in complex with cyclopropyl[(3*R*)-3-({4-[6-hydroxy-2-(naphthalen-2-yl)-1*H*-benzimidazol-1-yl]pyrimidin-2-yl}amino)piperidin-1-yl]methanone (PDB code: 4KKH) was downloaded from the protein data bank (PDB) and used for the docking study. All extraneous ligands and ions were removed from the downloaded protein, retaining only the co-crystallized inhibitor and the binding sites water molecules. Enzyme preparation was performed using the default settings in MOE with the QuickPrep protocol. Energy minimization was conducted using the same software until a root-mean-square deviation (RMSD) gradient of 0.1 kcal mol^−1^ Å^−1^ was reached, employing the Amber 10: EHT forcefield. The partial charges were automatically calculated. The Triangle Matcher method was used for placement, while the scoring function employed was London dG, with GBVI/WSA dG as the refinement scoring function, utilizing flexible docking. The docking protocol was validated by redocking the co-crystallized ligand into the binding site using the aforementioned settings. The validated protocol was then applied to dock the active compounds 3a, 6a, and 6c, then their binding behavior within the binding pocket were analyzed to predict their potential activity.

### 4.4. Molecular dynamic simulation study

Molecular dynamics (MD) simulations were performed using Molecular Operating Environment (2024.06), NPA simulation algorithm and Amber:EHT forcefield. Partial charges have been calculated and the energy of the molecular system minimized to an RMS gradient of 1.0. All systems were solvated in an orthorhombic box using simple point charge water molecules extended 15 Å away from any protein atom. The system was neutralized with 0.15 M NaCl. The simulation protocol included a starting relaxation step for 10 picoseconds (ps), heating step for 100 ps with temperature gradient from 10 to 300 K, NVT step at temperature 300 K for 100 ps, NPT step at temperature 300 K and pressure 100 kPa for 200 ps, followed by a final production phase of 100 ns. During the MD simulation, a time step of 2 femtoseconds (fs) was used while constraining the bond lengths of H atoms. The atomic coordinates of the system were saved every 500 ps along the MD trajectory. Protein RMSD, ligand RMSD and conformational clustering were calculated using MOE svl commands. The Dynamic Player tool reads the MD trajectory file and identifies ligand/target interactions repeatedly occurring during the simulation time.

### 4.5. *In silico* prediction of the ADME properties and BBB permeability

ADME properties and BBB permeability for compounds 3a, 6a, and 6c were predicted using Deep-PK web tool (https://biosig.lab.uq.edu.au/deeppk/).^136^

## Conflicts of interest

The authors declare that there is no conflict of interest regarding the publication of this work.

## Supplementary Material

RA-016-D5RA07584J-s001

## Data Availability

All data generated or analyzed during this study are included in this article and its supplementary information (SI) files. Supplementary information is available. See DOI: https://doi.org/10.1039/d5ra07584j.
