# Supplementary material for: Design and synthesis of new phosphazine and triazole derivatives for treatment of Alzheimer's disease: modulating ROS/JNK and Wnt/β-catenin signaling pathways
Source: RSC Adv. 2026 Jan 14;16(4):3077–100. doi: 10.1039/d5ra07584j (PMC12800931; doi:10.1039/d5ra07584j)
Supplement: RA-016-D5RA07584J-s001 [file RA-016-D5RA07584J-s001.pdf]

Supplementary Information (SI) for RSC Advances.
This journal is © The Royal Society of Chemistry 2025

Appendix A

Synthesis of new phosphazine and triazole derivatives for treatment of Alzheimer's disease: modulating ROS/JNK and Wnt/β-catenin signaling pathways

Rania S. Salah[a], Naglaa F. El- Sayed[b], Marwa El-Hussieny[b], Shaimaa T. Mansour[b], Mohamed Othman[c], Marwa A. Fouad[d,e], Huda R. M. Rashdan[f], Ewies F. Ewies[b]*, Heba S.A. Gharib[g], Ghada H. Elsayed[a,h]*

[a] Hormones Department, Medical Research and Clinical Studies Institute, National Research Centre, 33 El-Bohouth St., Dokki, 12622, Giza, Egypt.
[b] Organometallic and Organometalloid Chemistry Department, National Research Centre, 33ElBohouth St., (Former El Tahrir) Dokki, P.O. 12622, Giza, Egypt.
[c] Normandie Univ.; UNILEHAVRE, FR 3038 CNRS, URCOM 76600 Le Havre, France. UR 3221, INC3M CNRS-FR 3038 UFR ST, BP: 1123, 25 rue Philipe Lebon, 76063 Le Havre Cedex (France).
[d] Pharmaceutical Chemistry Department, Faculty of Pharmacy, Cairo University, Kasr El-Aini St., Cairo 11562, Egypt
[e] Pharmaceutical Chemistry Department, School of Pharmacy, Newgiza University, NewGiza, Km 22 Cairo-Alexandria Desert Road, Cairo, Egypt
[f] Chemistry of Natural and Microbial Products Department, Pharmaceutical and Drug Industries Research Institute, National Research Centre, 33 ElBohouth St., (Former El Tahrir) Dokki, P.O. 12622, Giza, Egypt.
[g] Behaviour and Management of animal, Poultry and Aquatics Department, Faculty of Veterinary Medicine, Zagazig University, P.O. Box 44511, Zagazig, Egypt.
[h] Stem Cells Lab, Centre of Excellence for Advanced Sciences, National Research Centre, 33 El-Bohouth St., Dokki, 12622, Giza, Egypt.

Corresponding authors:
Ewies F. Ewies: Organometallic and Organometalloid Chemistry Department, National Research Centre, 33 ElBohouth St., (Former El Tahrir) Dokki, P.O. 12622, Giza, Egypt. email: ewiesfawzy@yahoo.com ; ef.ewies@nrc.sci.eg
Ghada H. Elsayed: Hormones Department, Medical Research and Clinical Studies Institute, National Research Centre, 33 El-Bohouth St., Dokki, 12622, Giza, Egypt.
Stem Cells Lab, Centre of Excellence for Advanced Sciences, National Research Centre, 33 El-Bohouth St., Dokki, 12622, Giza, Egypt. email: ghadanrc@yahoo.com

Experimental

Chemistry

All chemicals were supplied by either Fluka or Aldrich chemical companies and were used without further purification. All melting points are uncorrected and were taken in open capillary tubes using Electrothermal apparatus 9100. Elemental microanalyses were carried out at Microanalytical Unit, Central Services Laboratory, National Research Centre, Dokki, Giza, Egypt, using Vario Elementar and were found within ±0.4% of the theoretical values. **FT-IR** spectra were recorded with a Perkin-Elmer Frontier. Routine **NMR** spectra were recorded at room temperature on a Bruker Avance TM 300 or 400 spectrometer as solutions in dimethyl sulfoxide (DMSO) or in chloroform ($CDCl_3$). All chemical shifts are quoted in δ relative to the trace resonance of protonated chloroform (δ 7.25 ppm), $CDCl_3$ (δ 77.0 ppm) or dimethyl sulfoxide (δ 2.50 ppm), DMSO (δ 39.51 ppm) and external 85% aqueous H_3PO_4 (δ 0.0 ppm). The mass spectra were measured with a GC Finnigan MAT SSQ-7000 mass spectrometer. Follow up of the reactions and checking the purity of the compounds were made by Thin Layer Chromatography (TLC) on silica gel-precoated aluminum sheets (Type 60, F 254, Merck, Darmstadt, Germany) with eluent of petroleum ether (b.r. 60-80°C)/ethyl acetate and the spots were detected by exposure to UV lamp at λ_{254} nanometer for a few seconds. The chemical names given for the prepared compounds are according to the IUPAC system. The reported yields are based upon pure materials isolated by column chromatography with eluent of petroleum ether (b.r. 60-80°C)/ethyl acetate. Solvents were dried/purified according to conventional procedures.

Experimental: Spectral Data

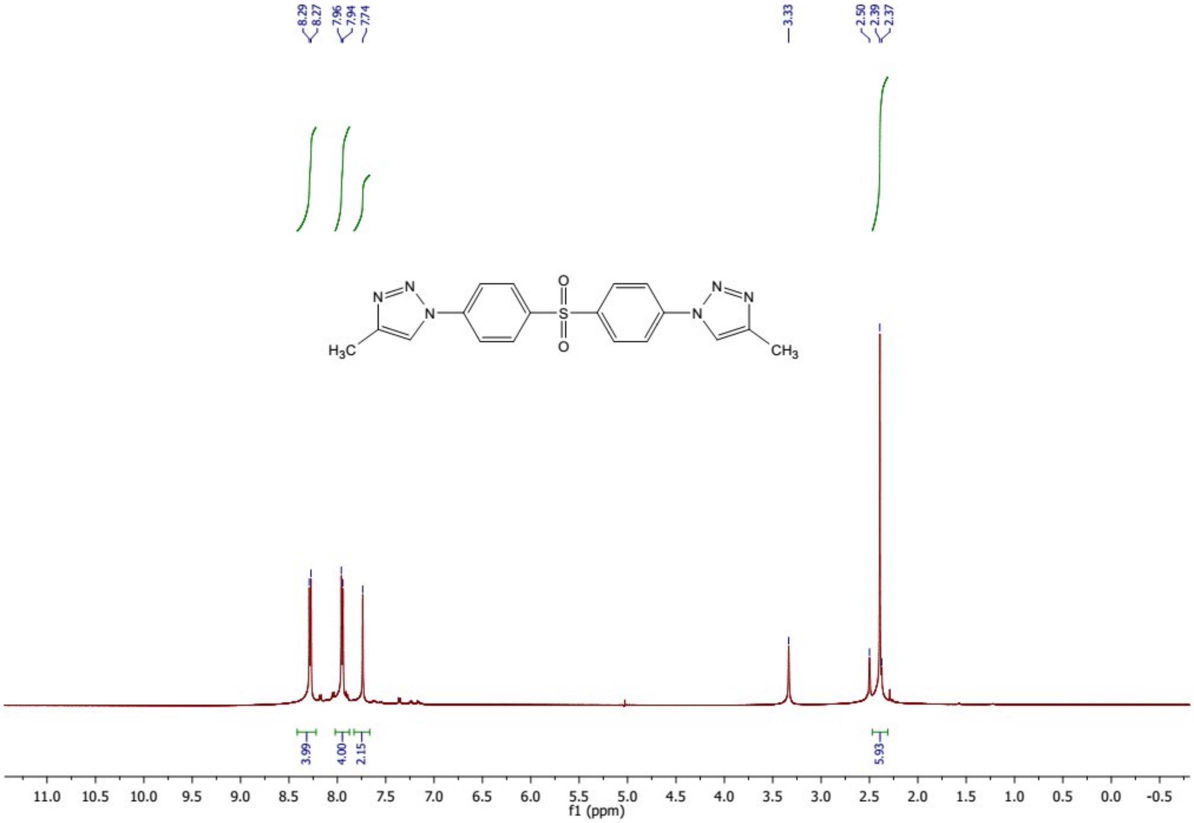

Figure : 1H NMR of compound 3a

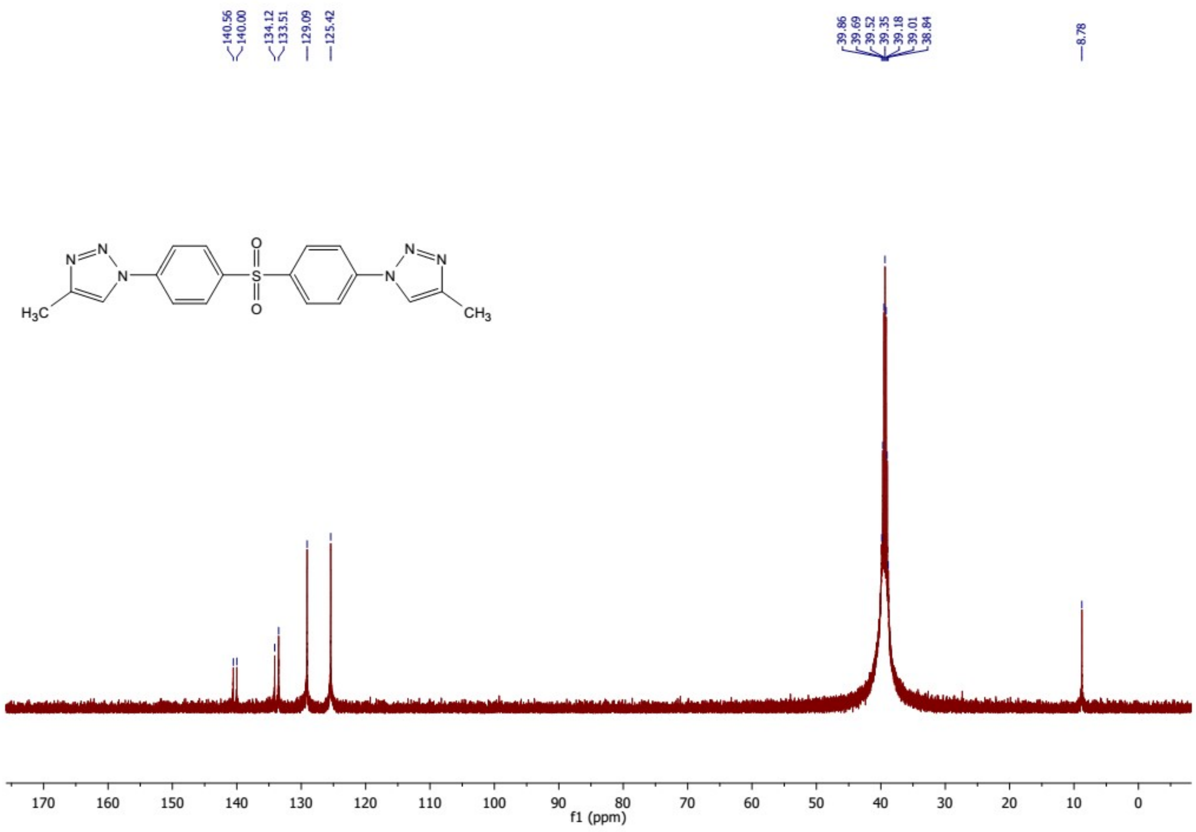

Figure : 13C NMR of compound 3a

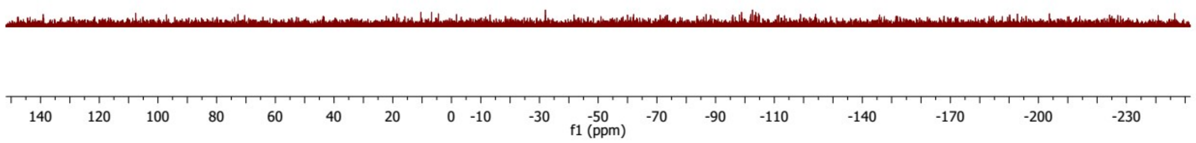

Figure : 13P NMR of compound 3a

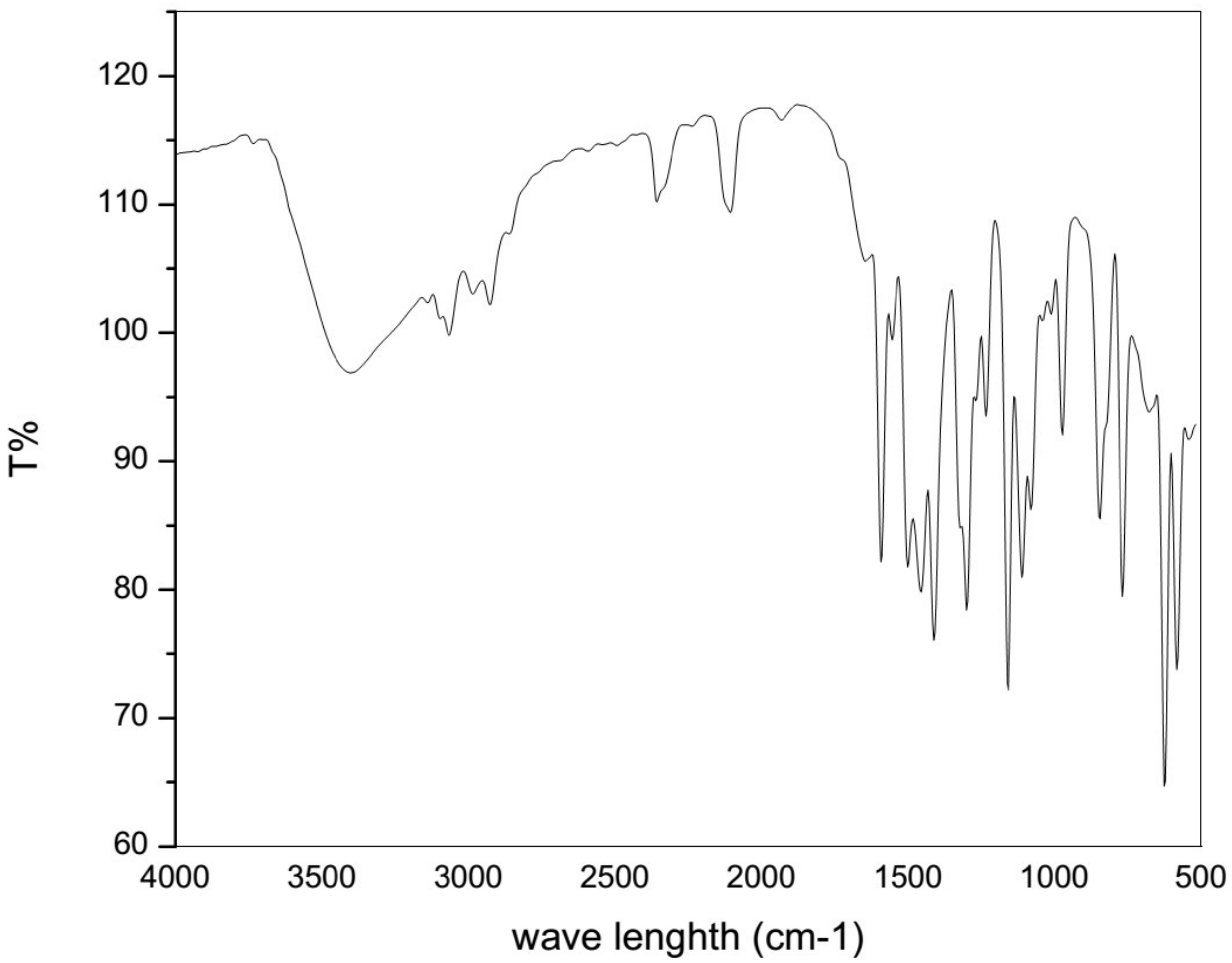

Figure : IR chart of compound 3a

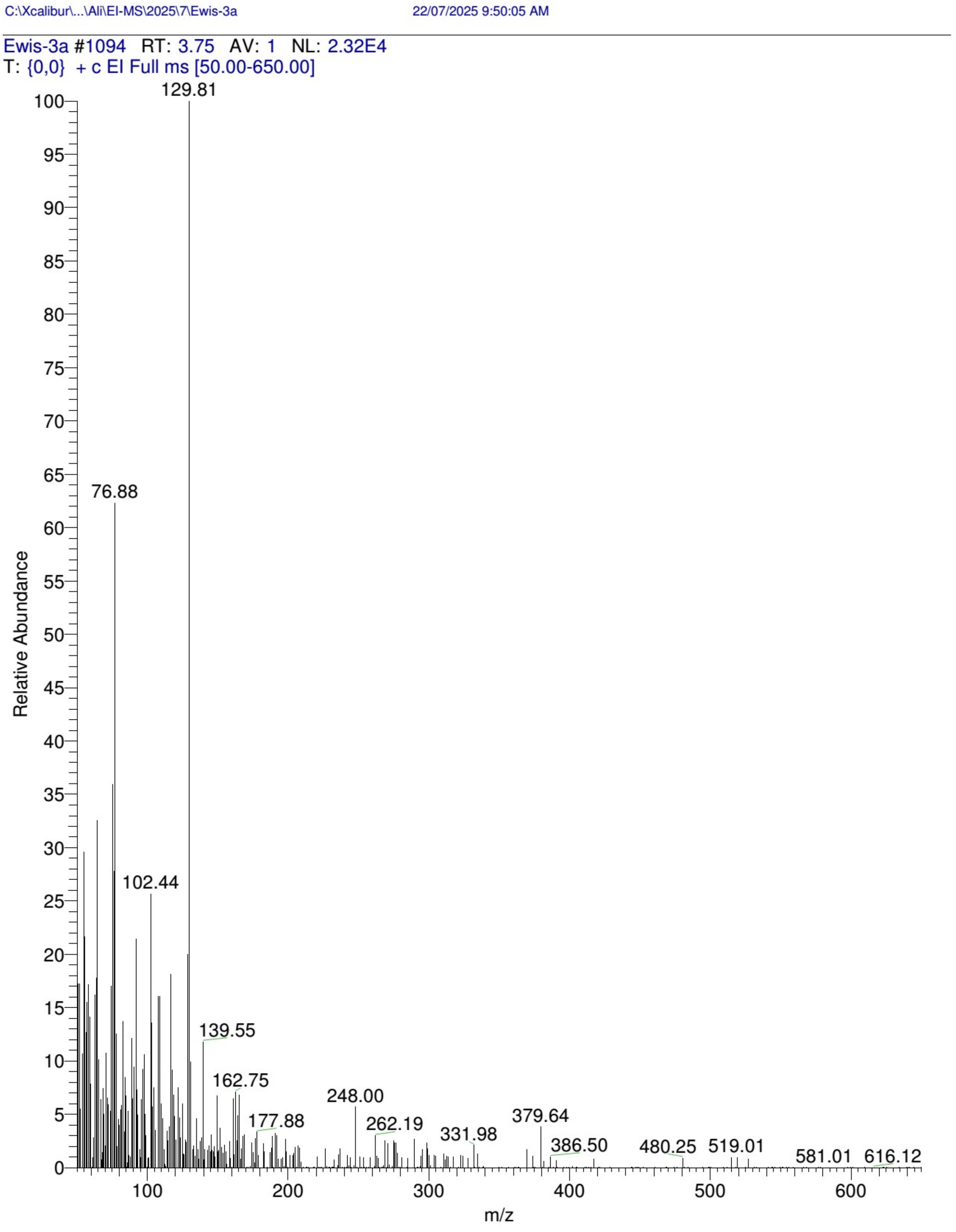

Figure : Mass spectrum of compound 3a

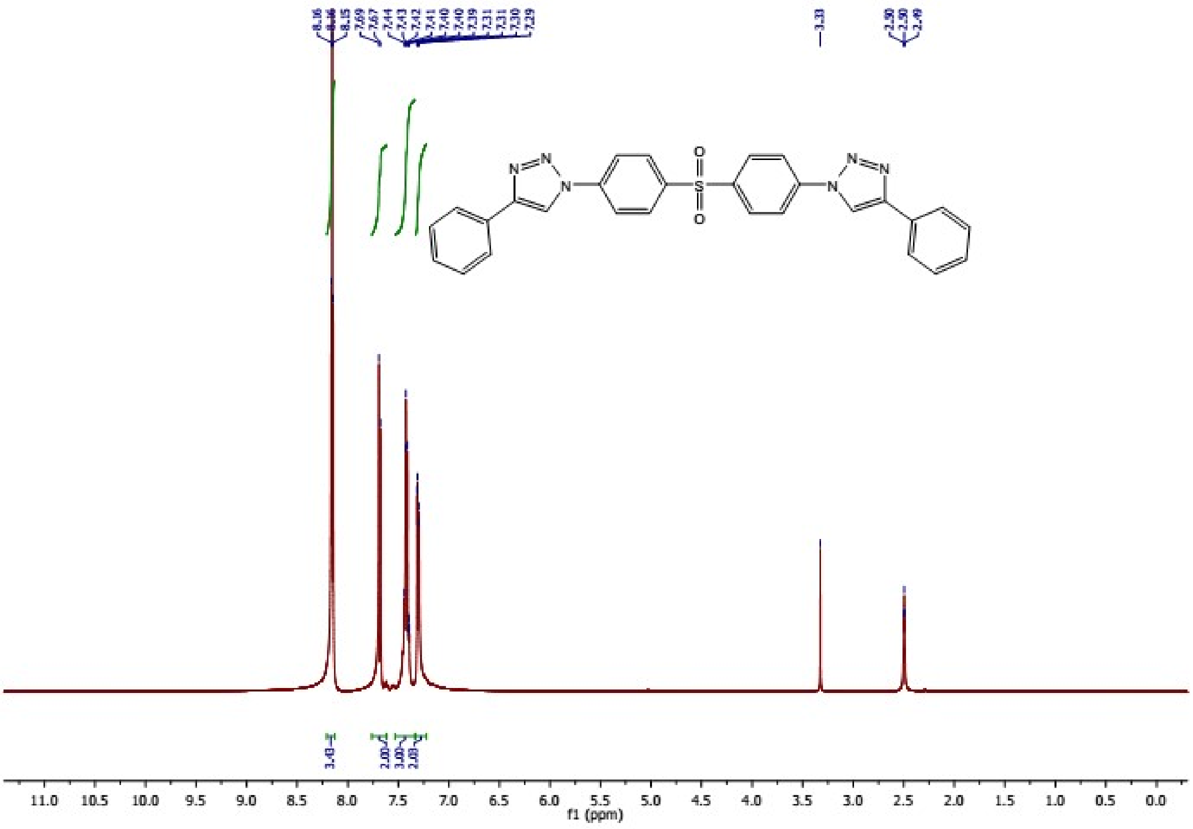

Figure : 1H NMR of compound 3b

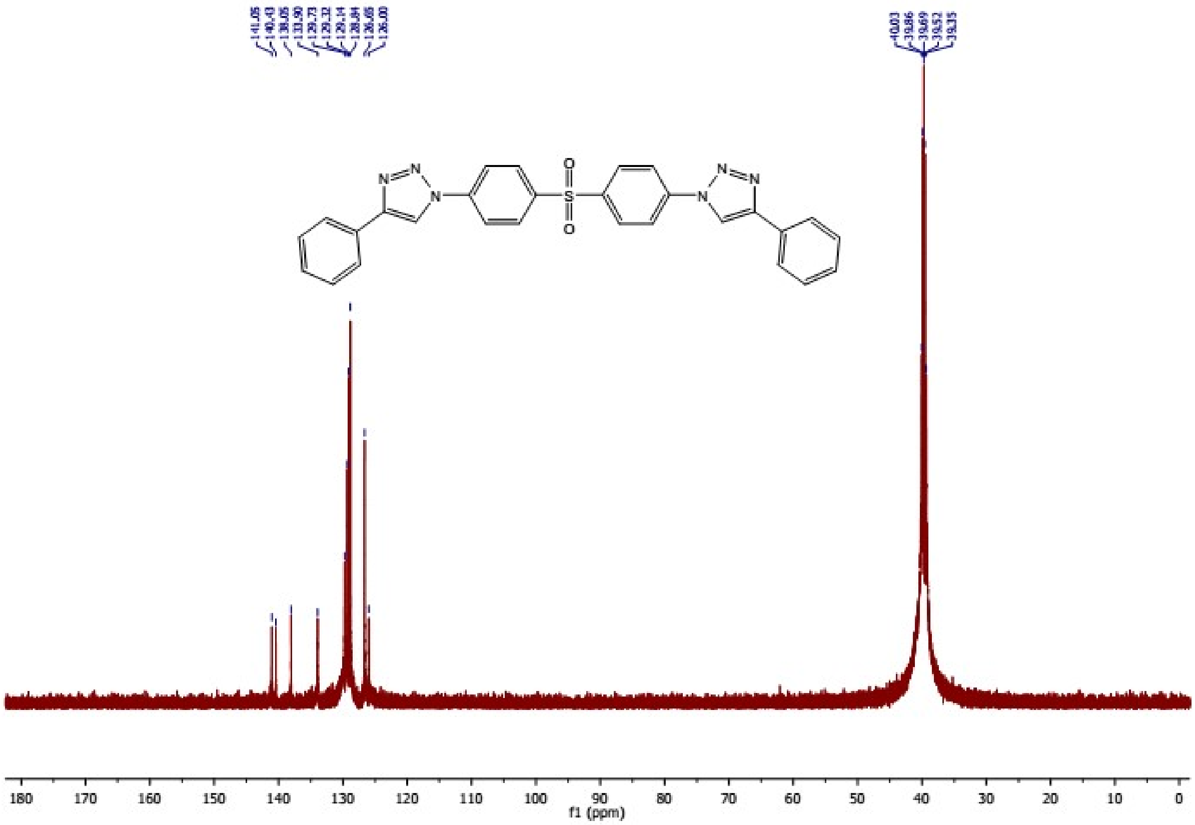

Figure : 13C NMR of compound 3b

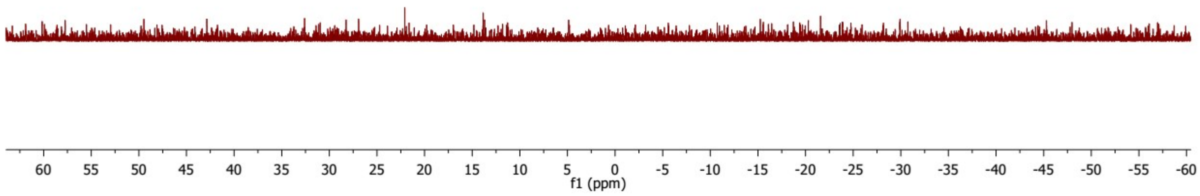

Figure : 13P NMR of compound 3b

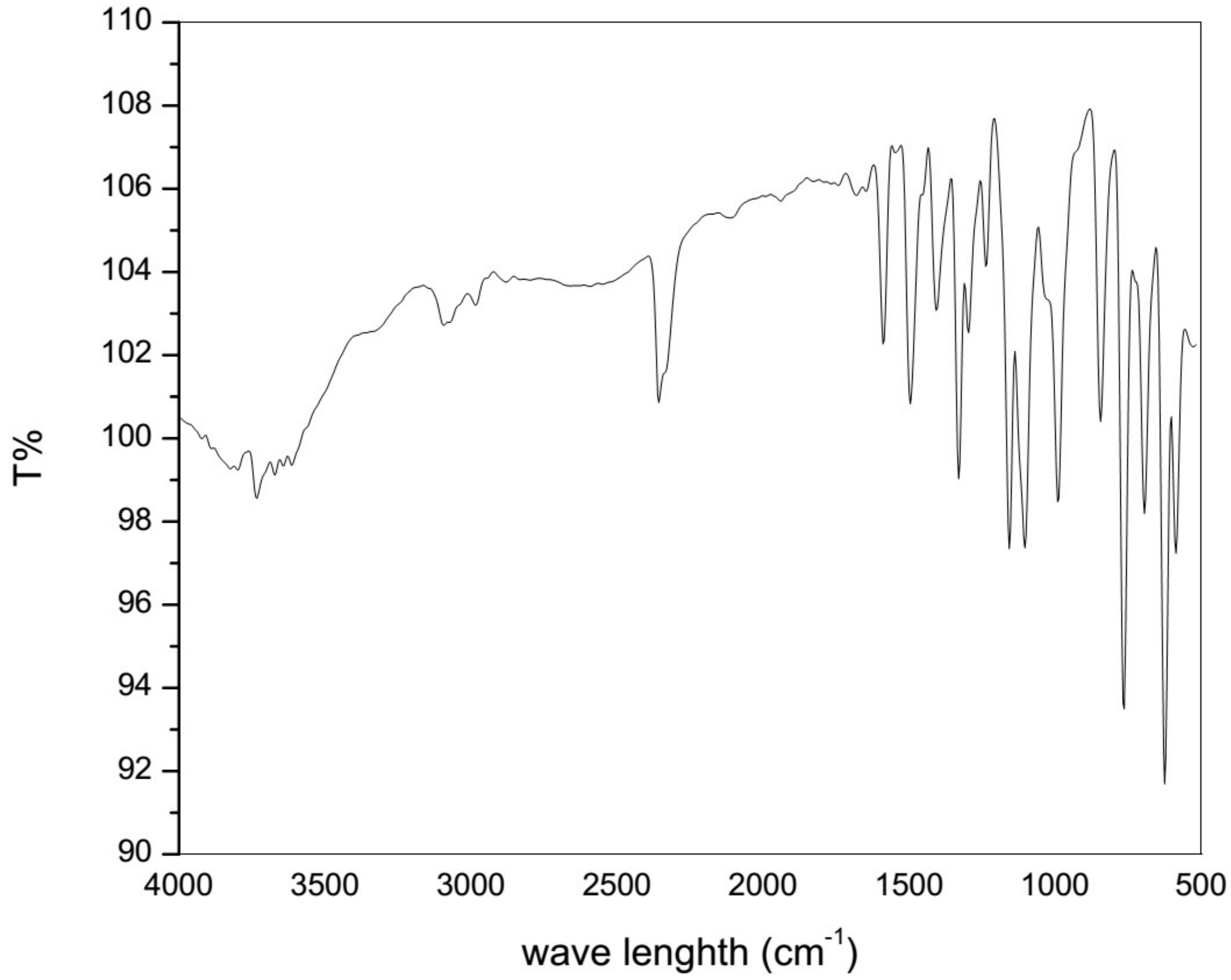

Figure : IR chart of compound 3b

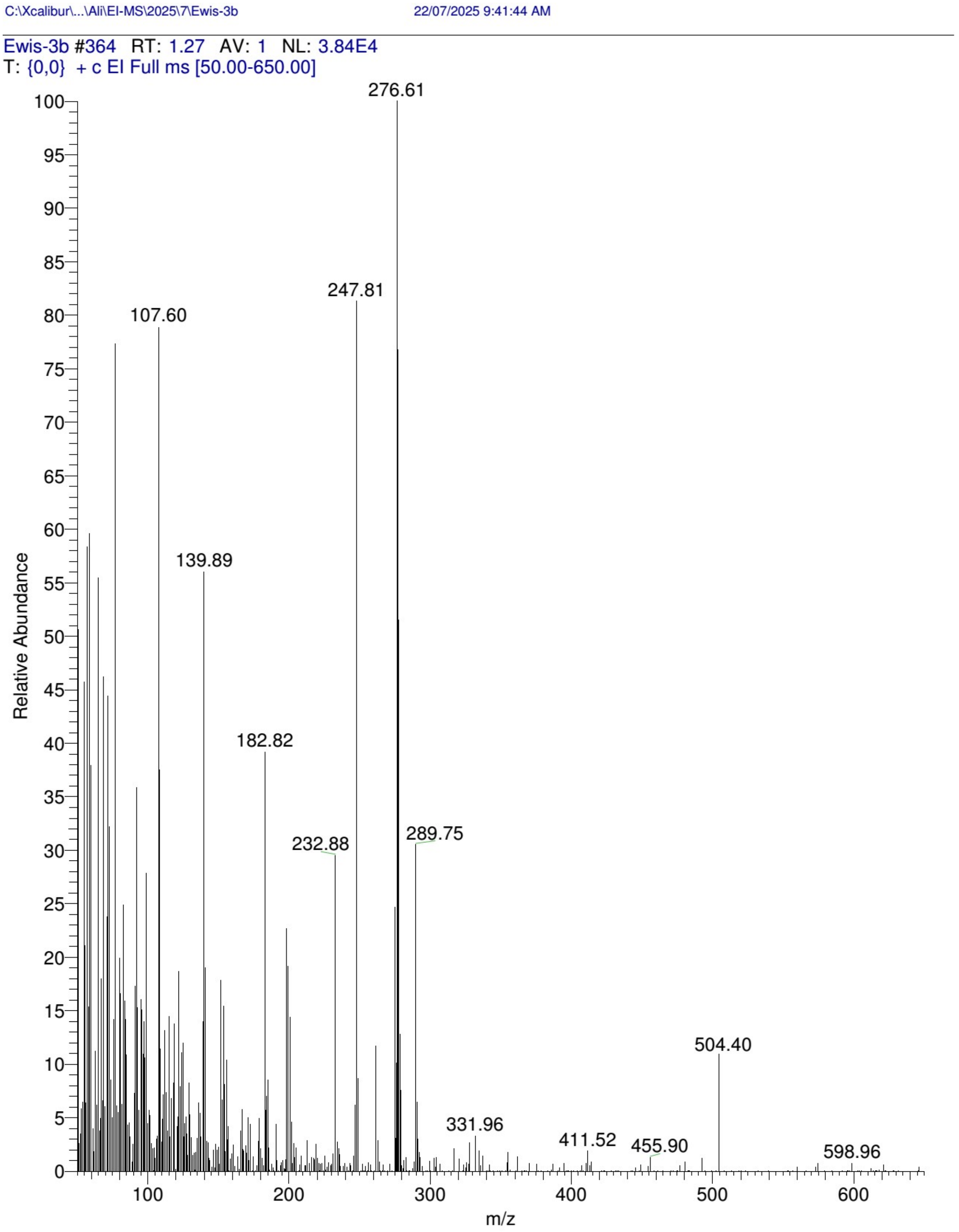

Figure : Mass spectrum of compound 3b

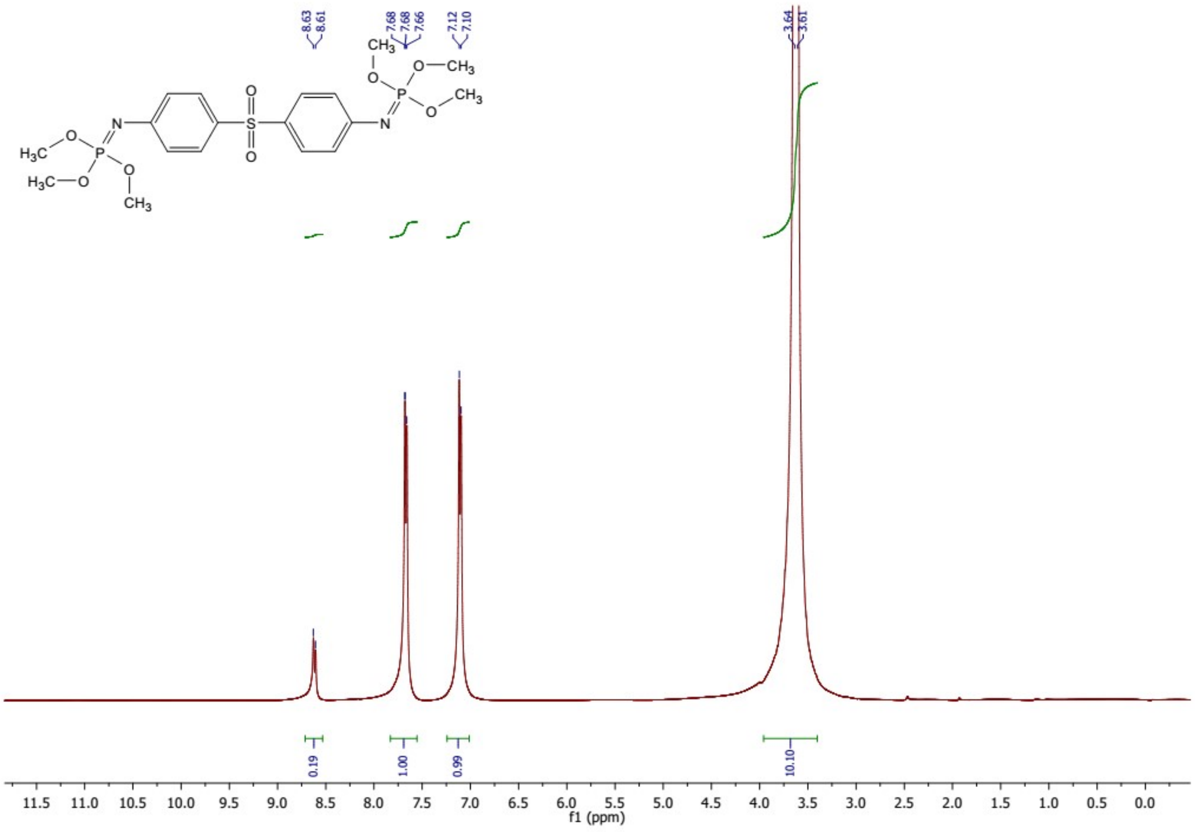

Figure : 1H NMR of compound 5a

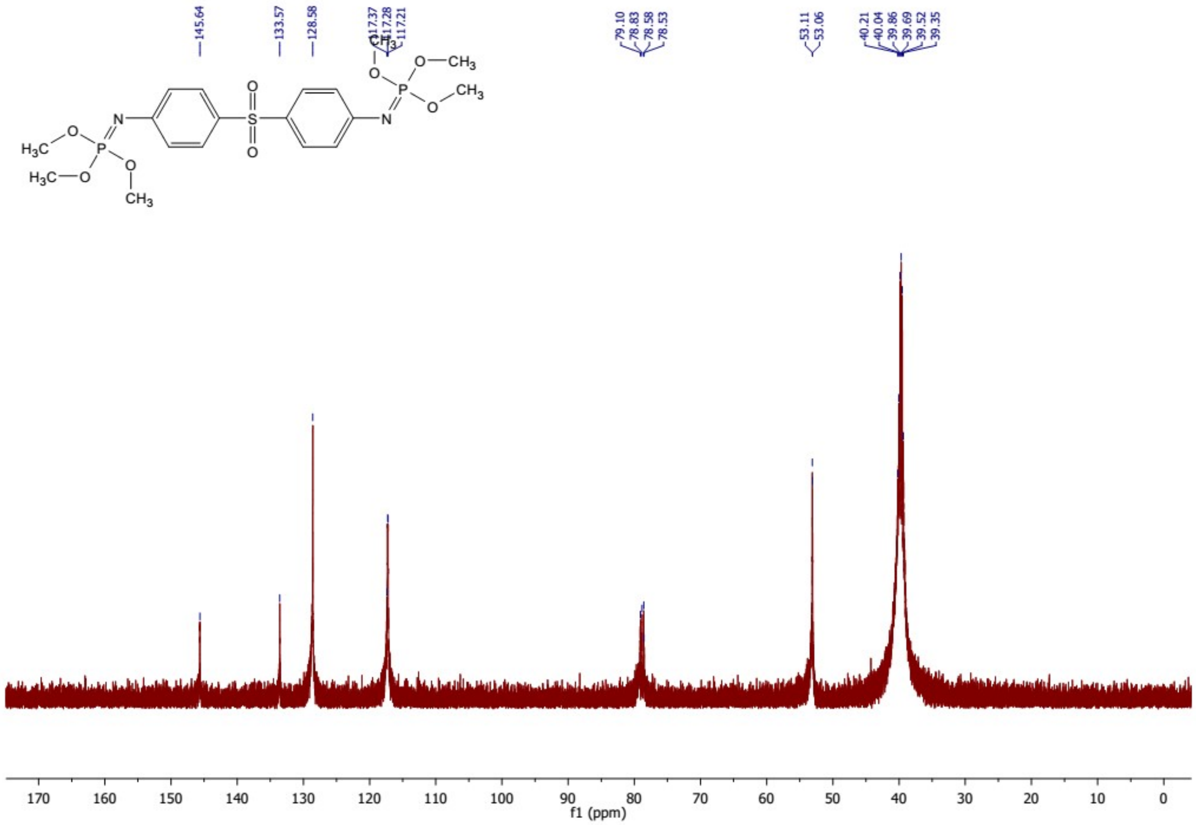

Figure : 13C NMR of compound 5a

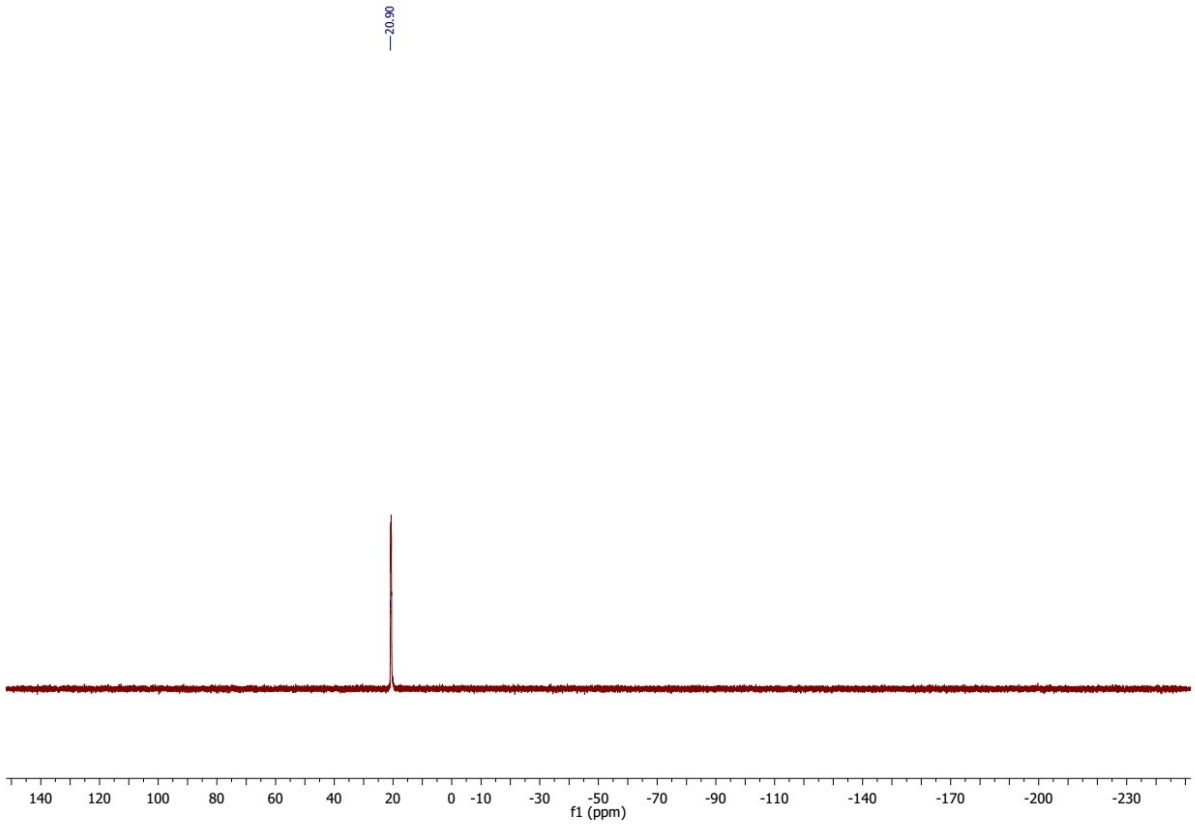

Figure : 13P NMR of compound 5a

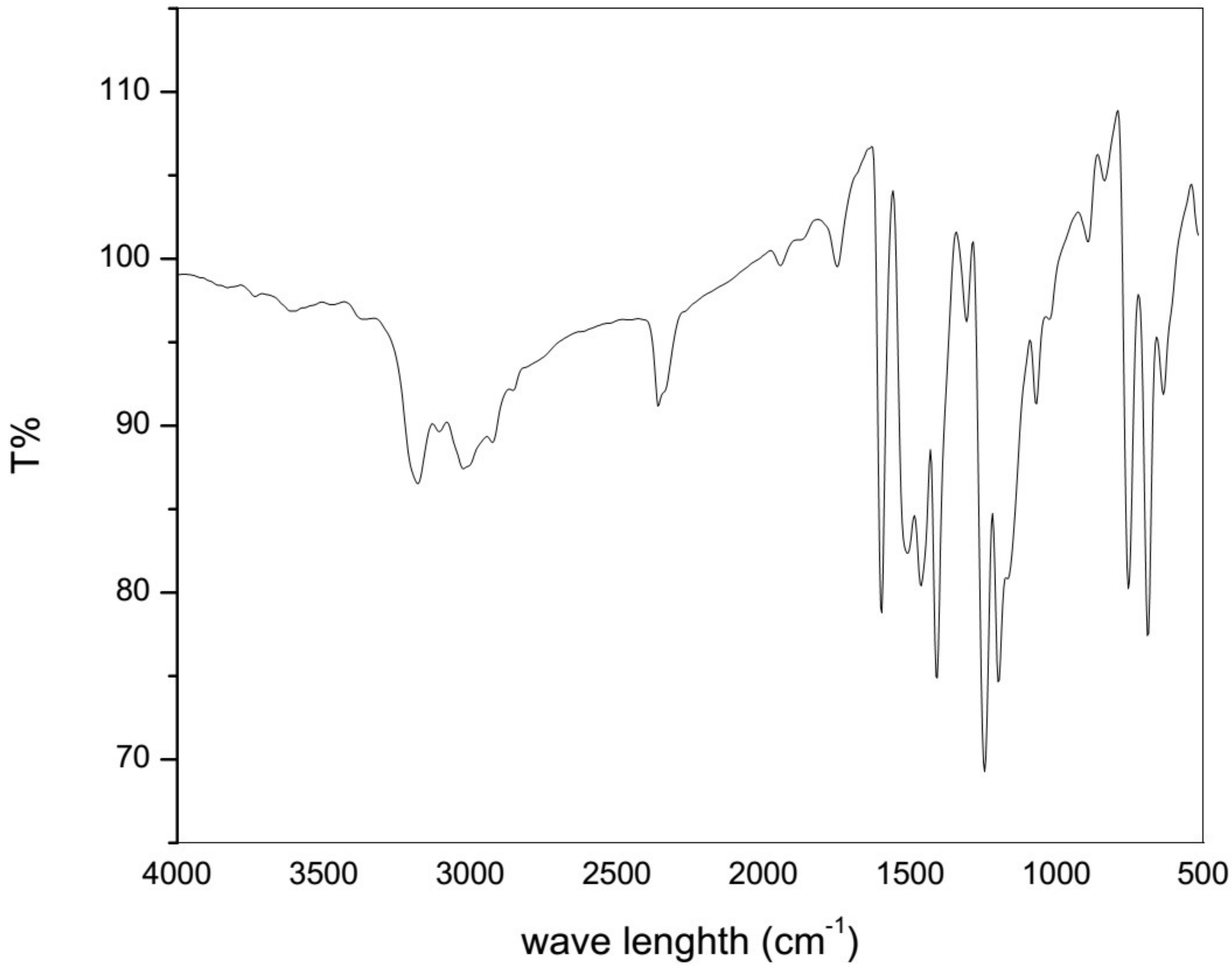

Figure : IR chart of compound 5a

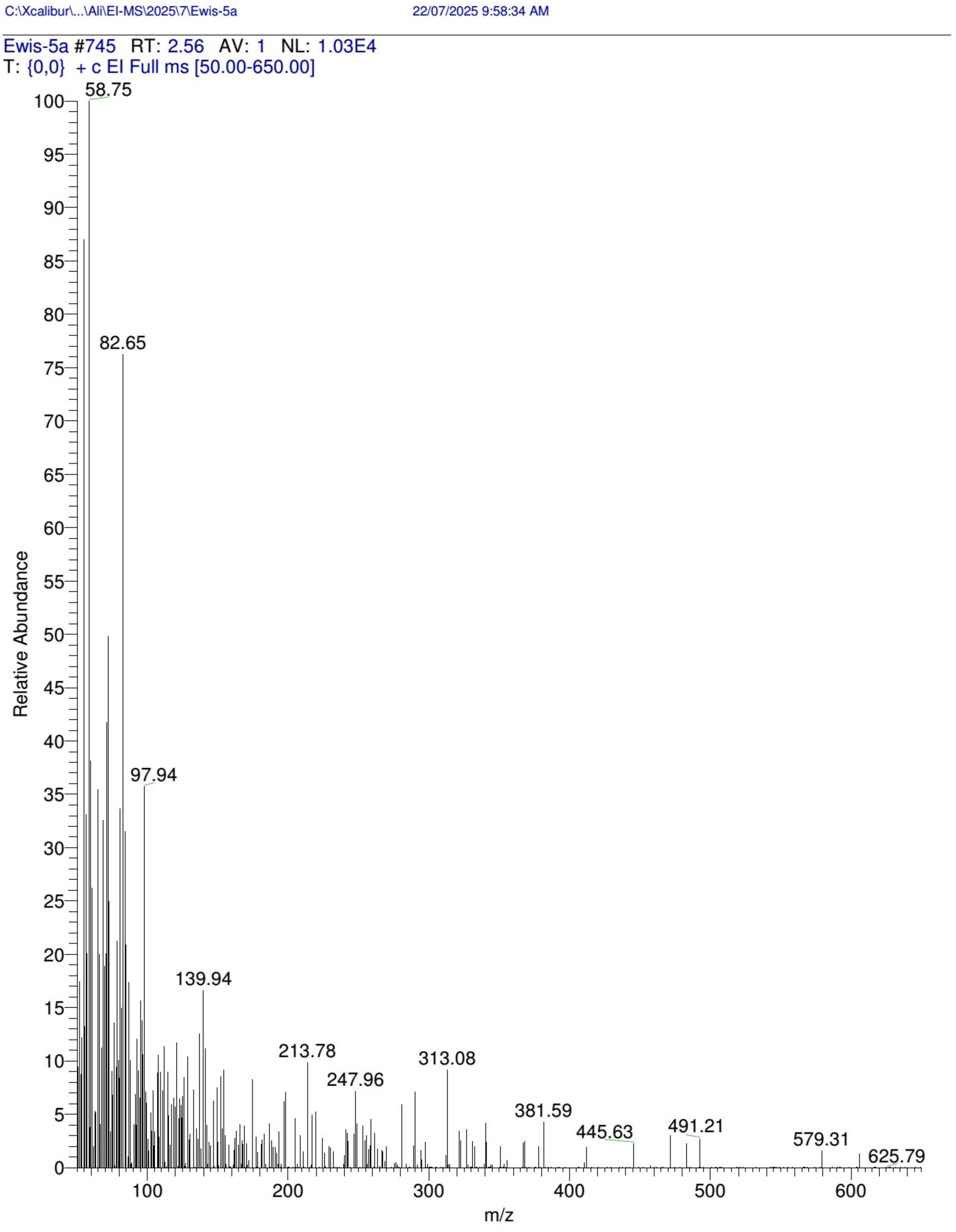

Figure : Mass spectrum of compound 5a

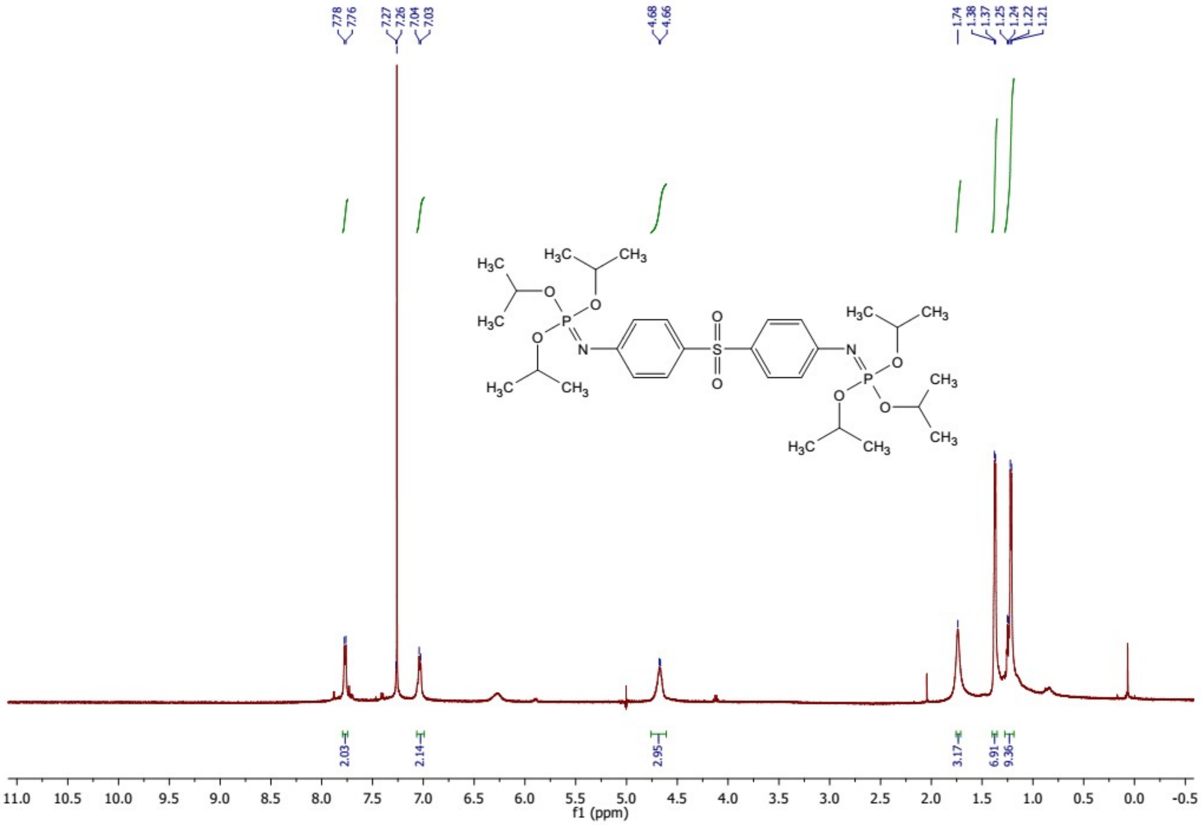

Figure : 1H NMR of compound 5b

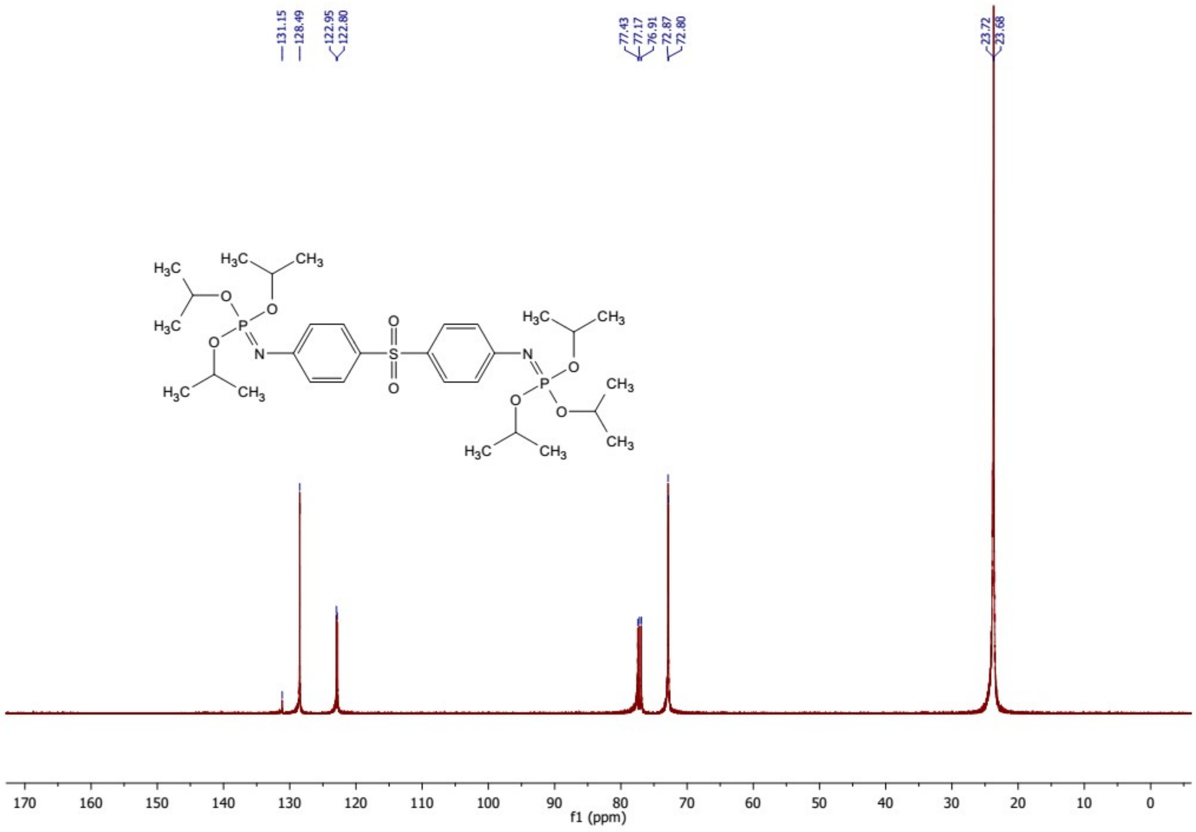

Figure : 13C NMR of compound 5b

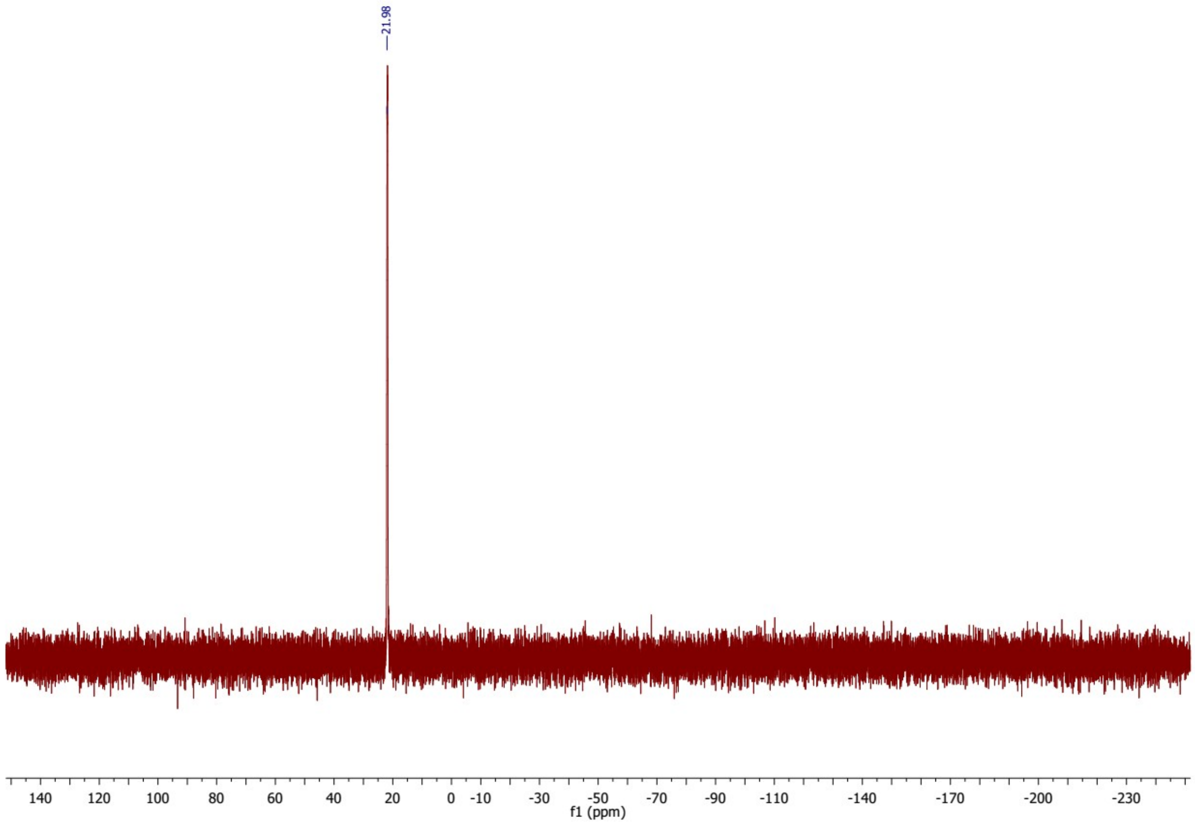

Figure : 13P NMR of compound 5b

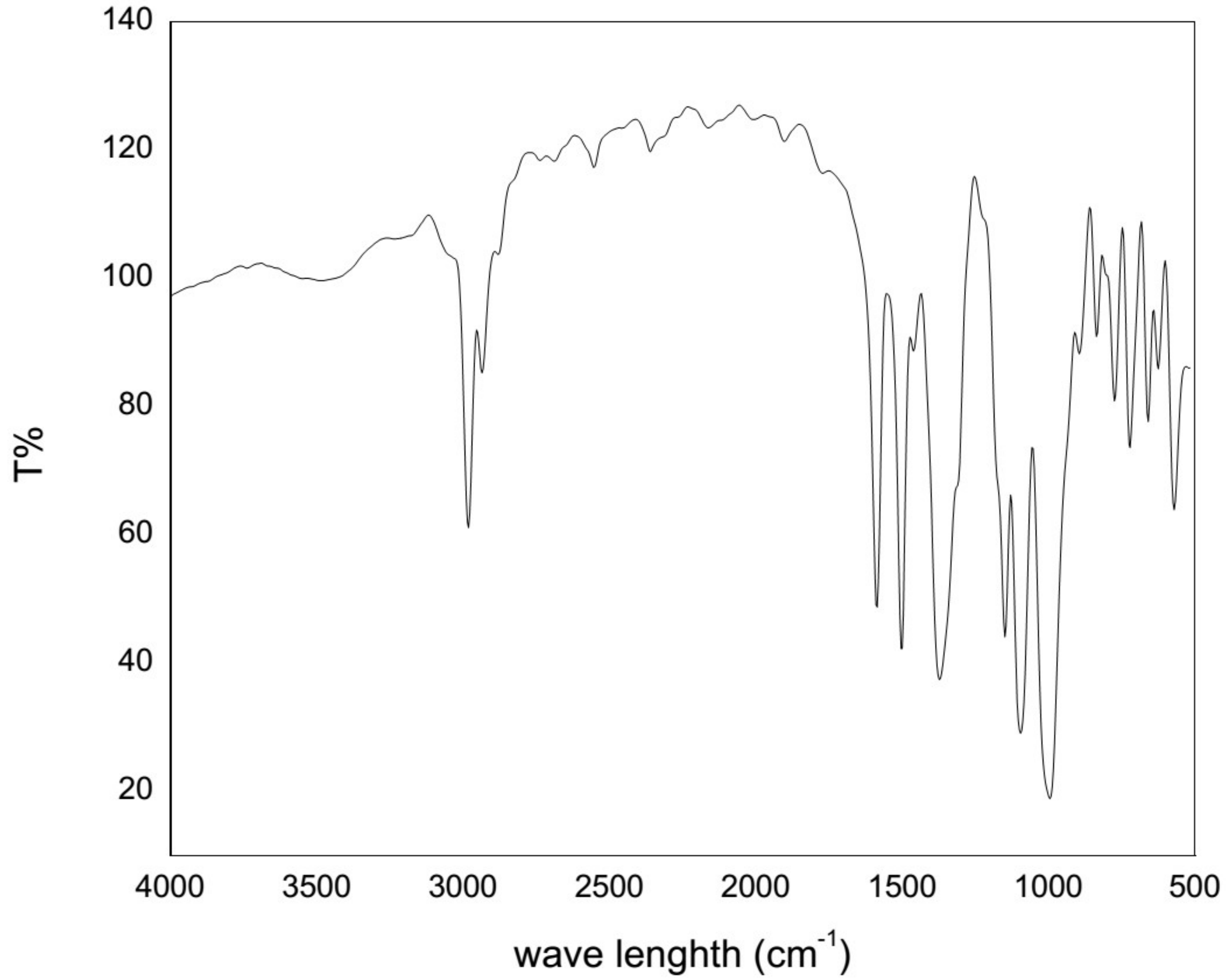

Figure : IR of compound 5b

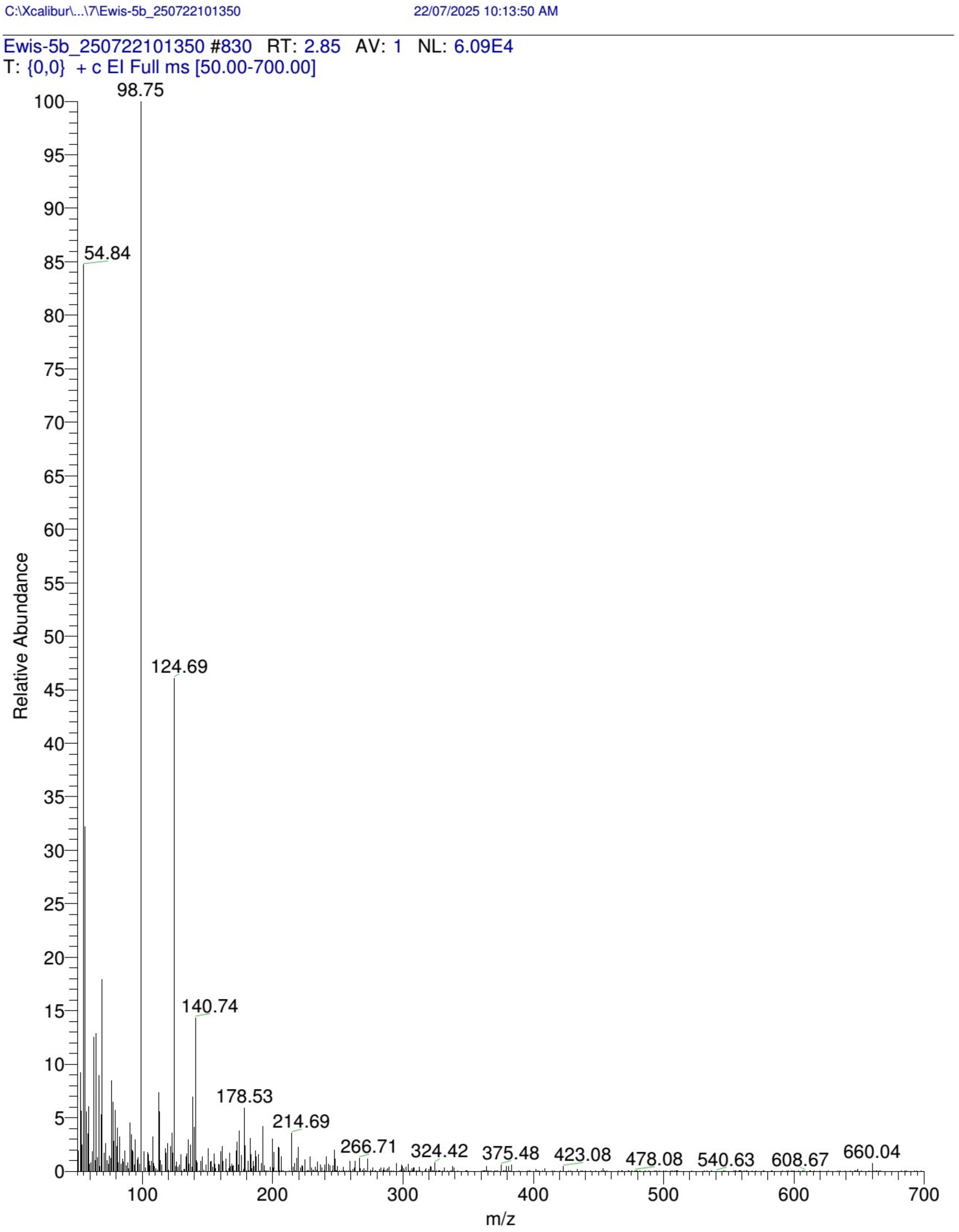

Figure : mass spectrum of compound 5b

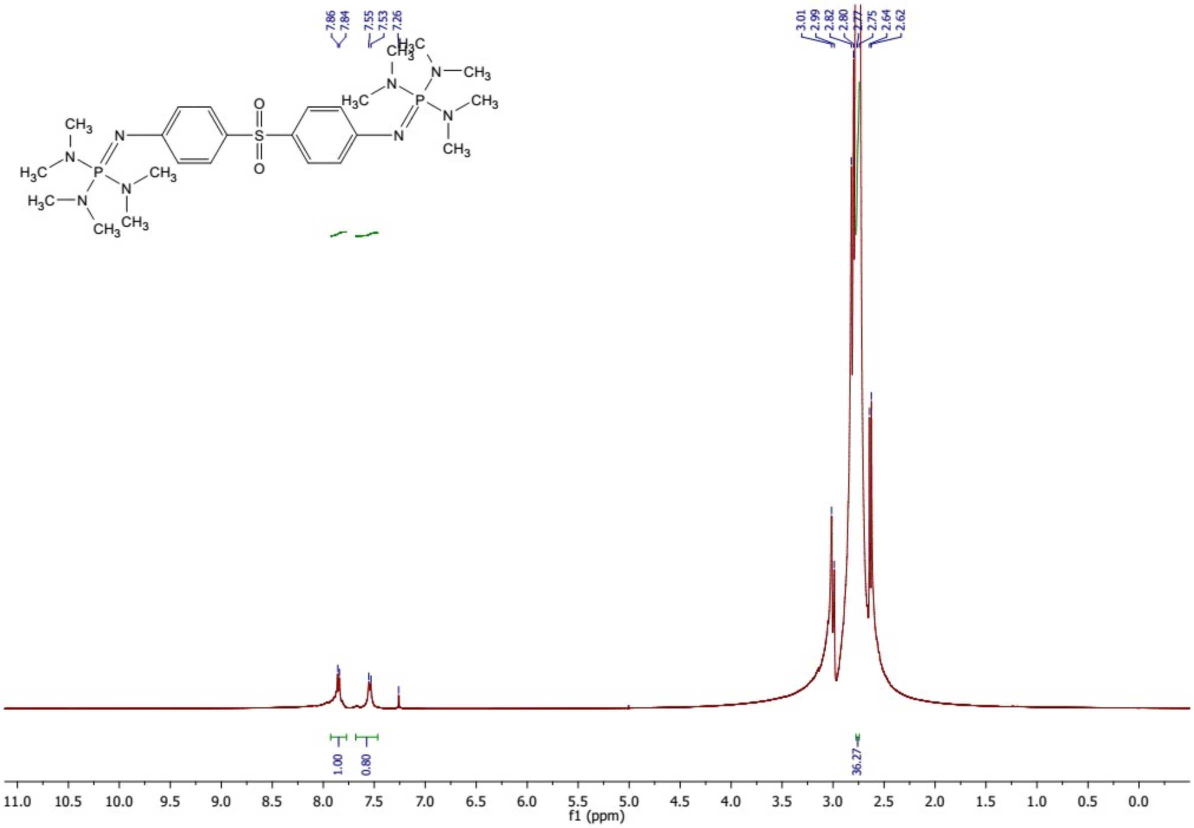

Figure : 1H NMR of compound 5c

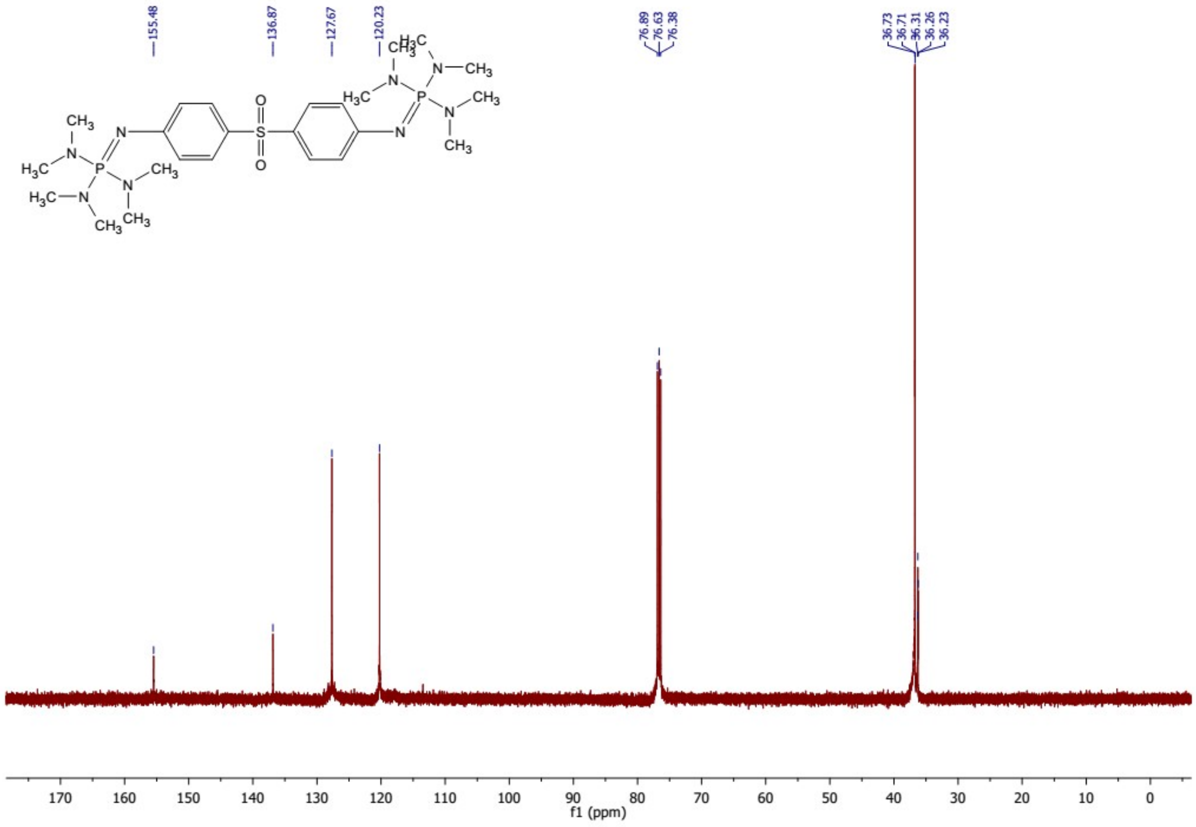

Figure : 13C NMR of compound 5c

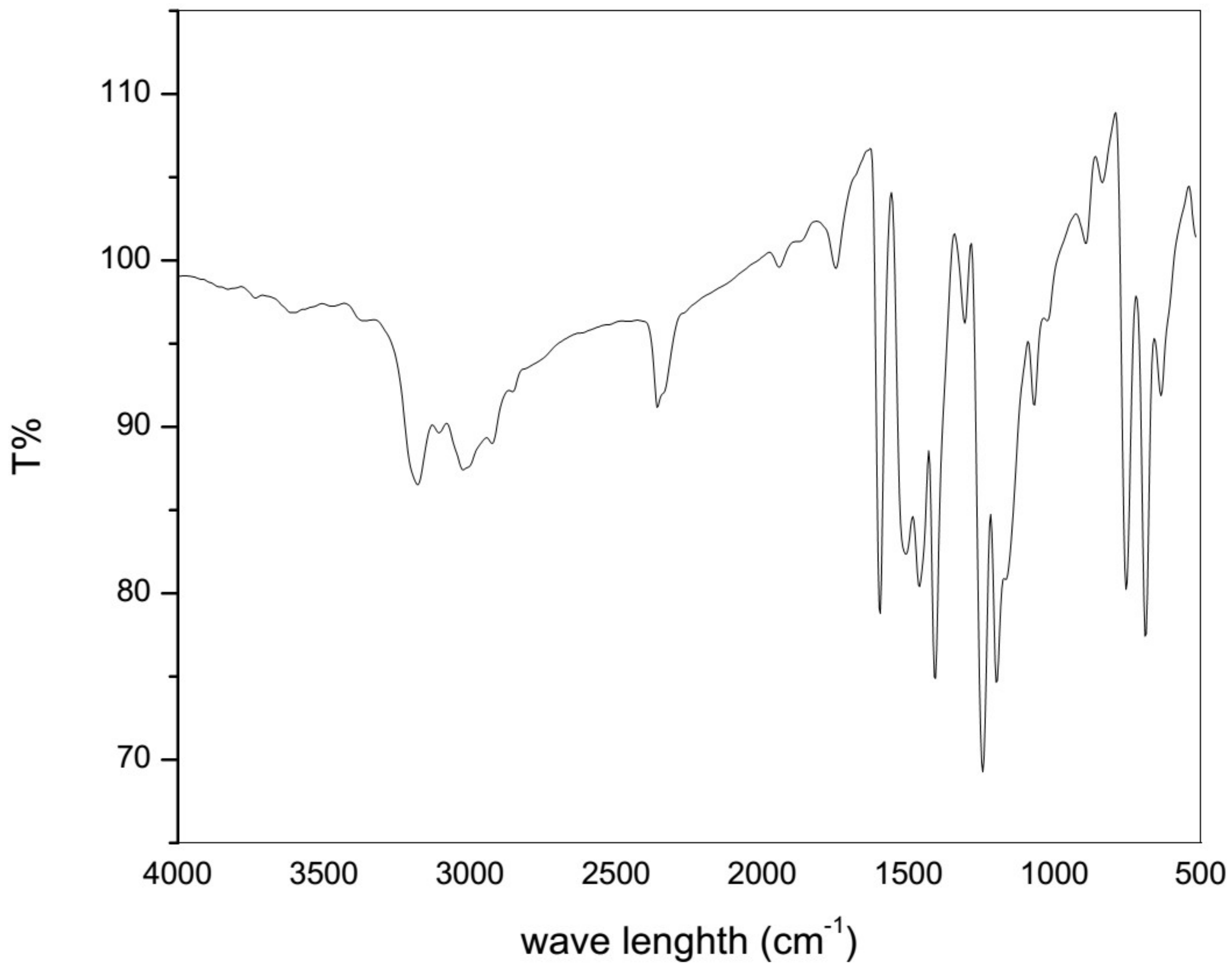

Figure : IR chart of compound 5c

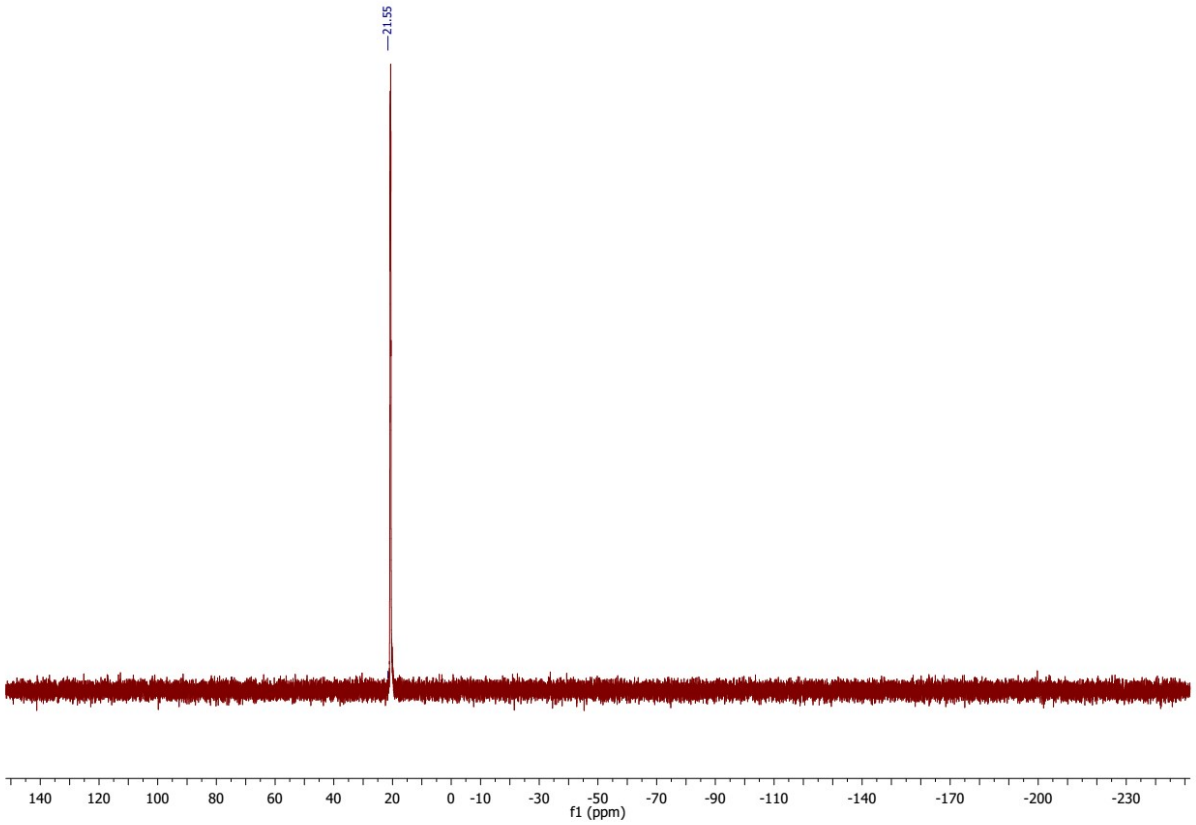

Figure : 13P NMR of compound 5c

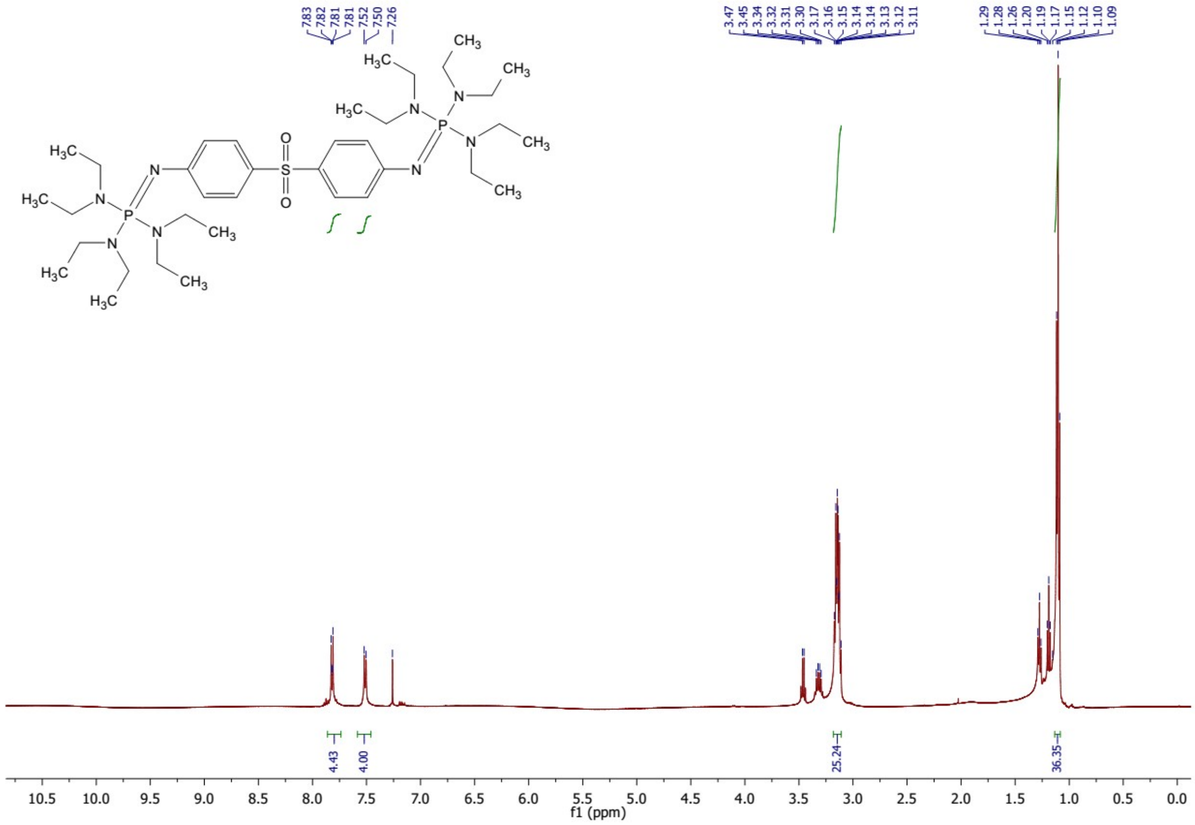

Figure : 1H NMR of compound 5d

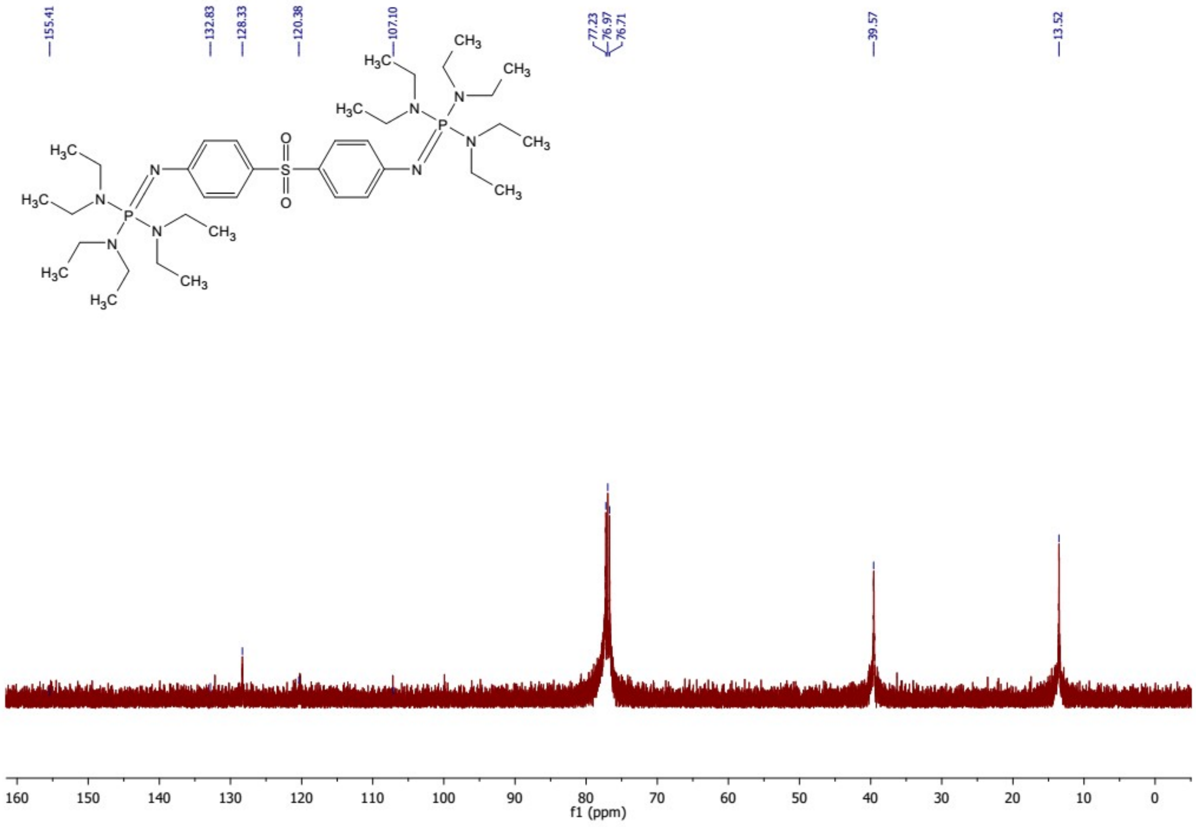

Figure : 13C NMR of compound 5d

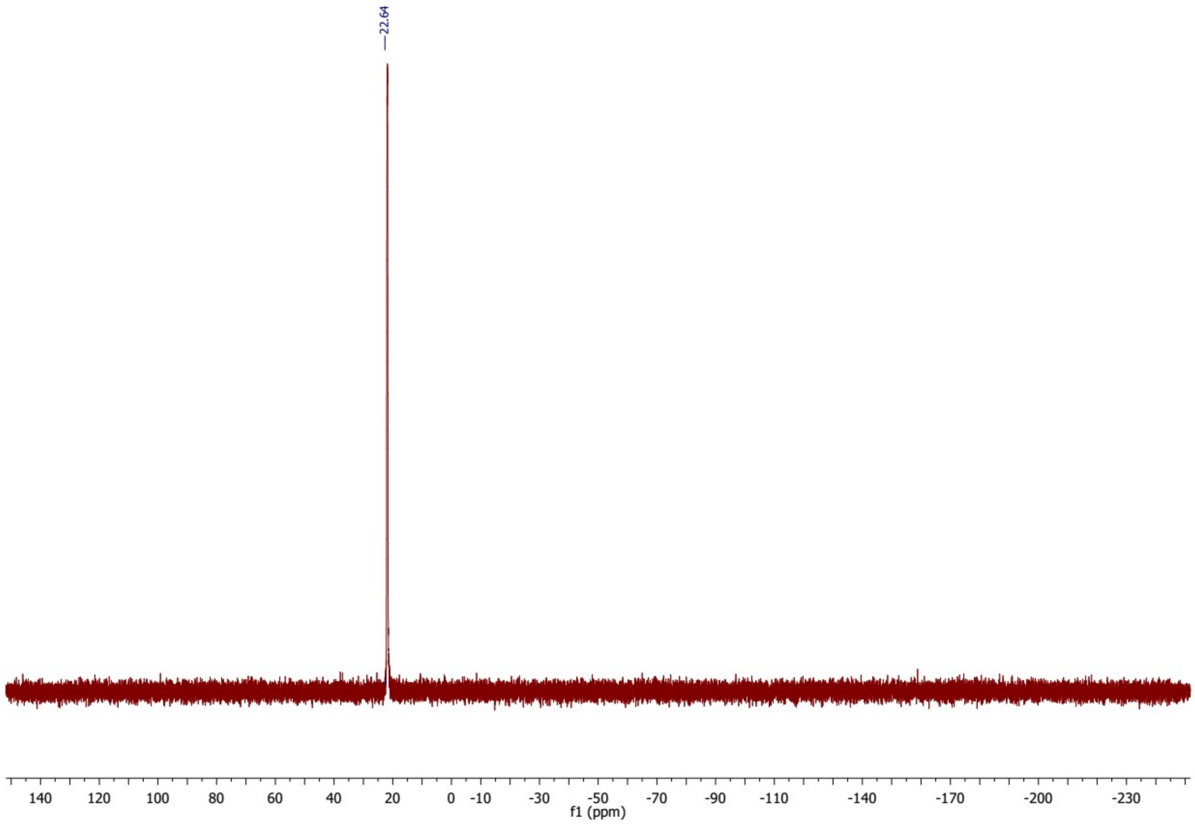

Figure : 13P NMR of compound 5d

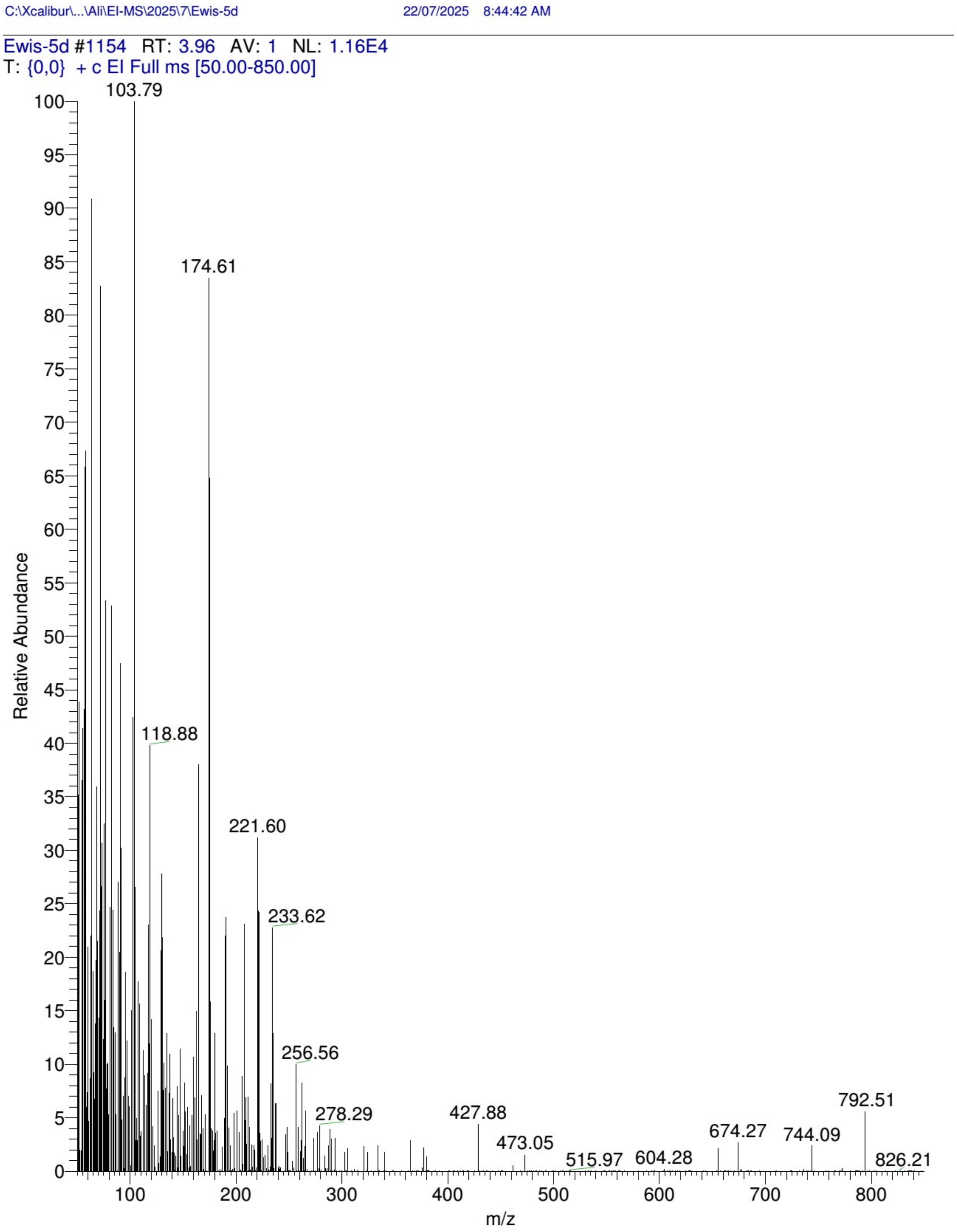

Figure : Mass spectrum of compound 5d

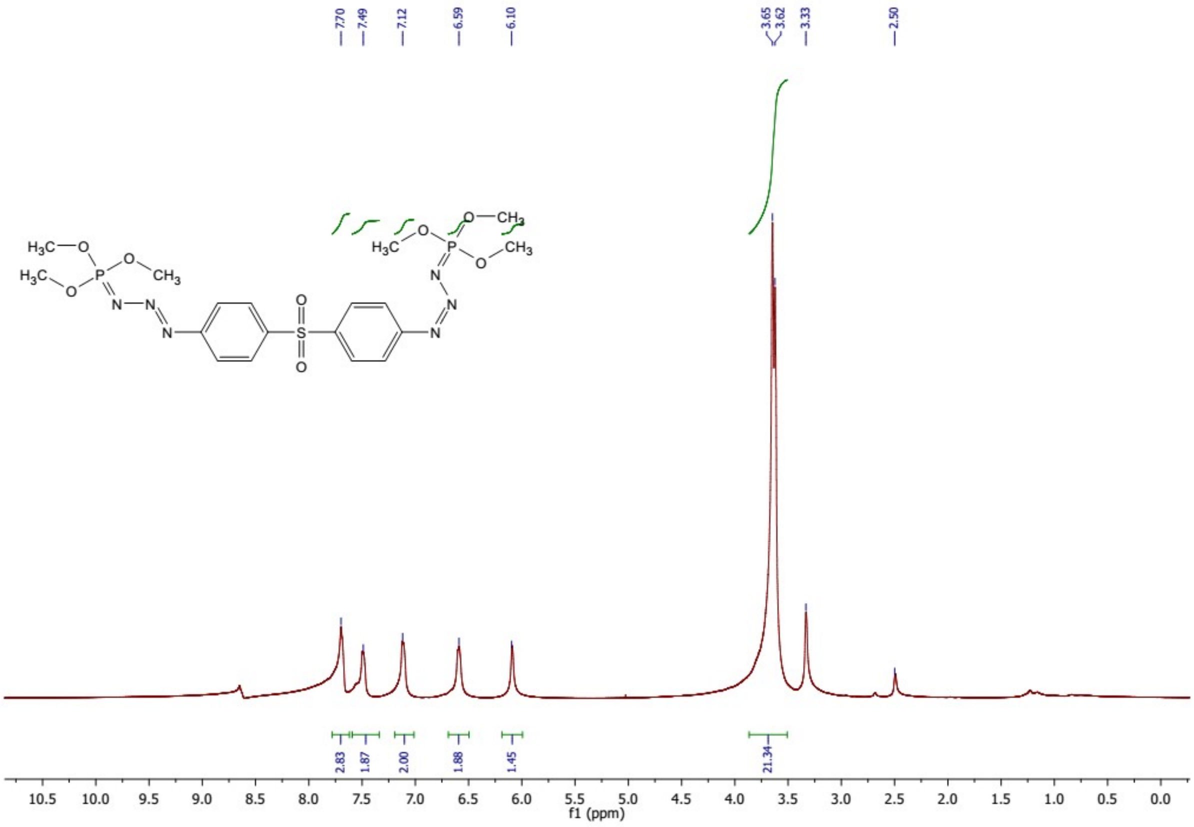

Figure : 1H NMR of compound 6a

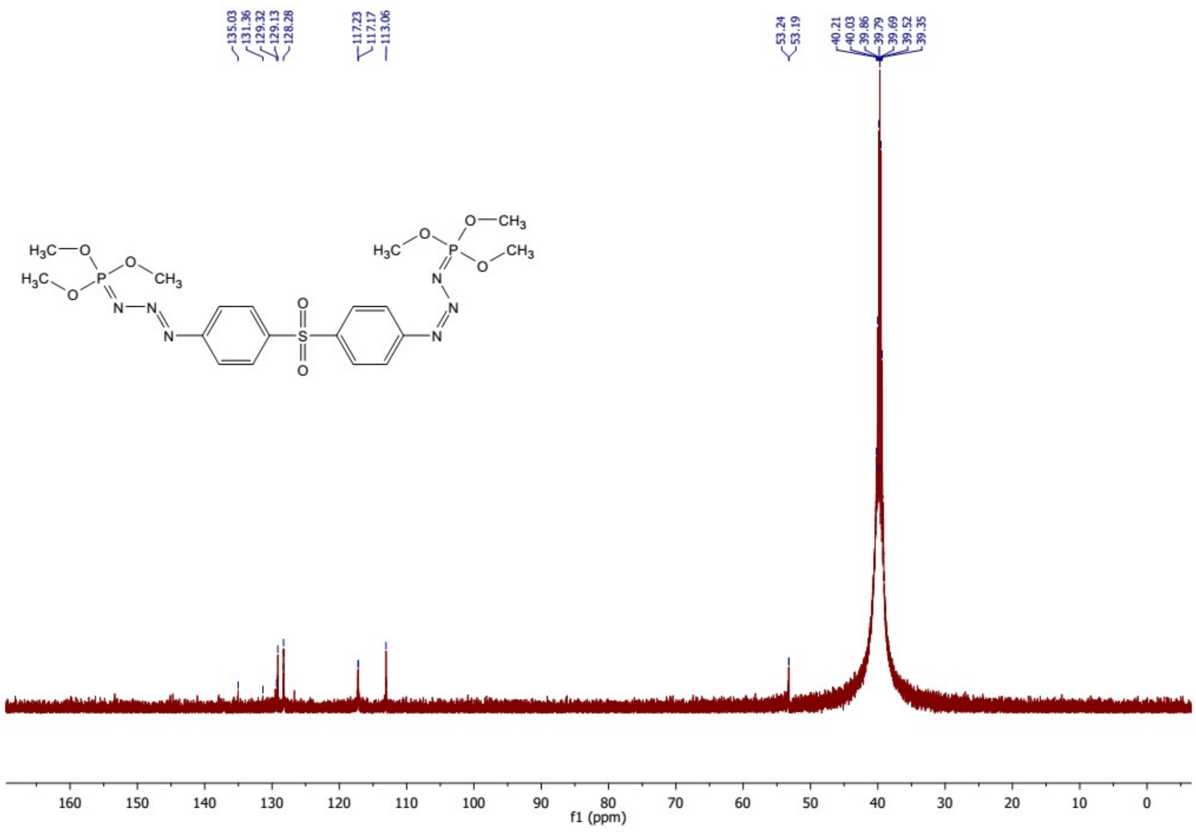

Figure : 13C NMR of compound 6a

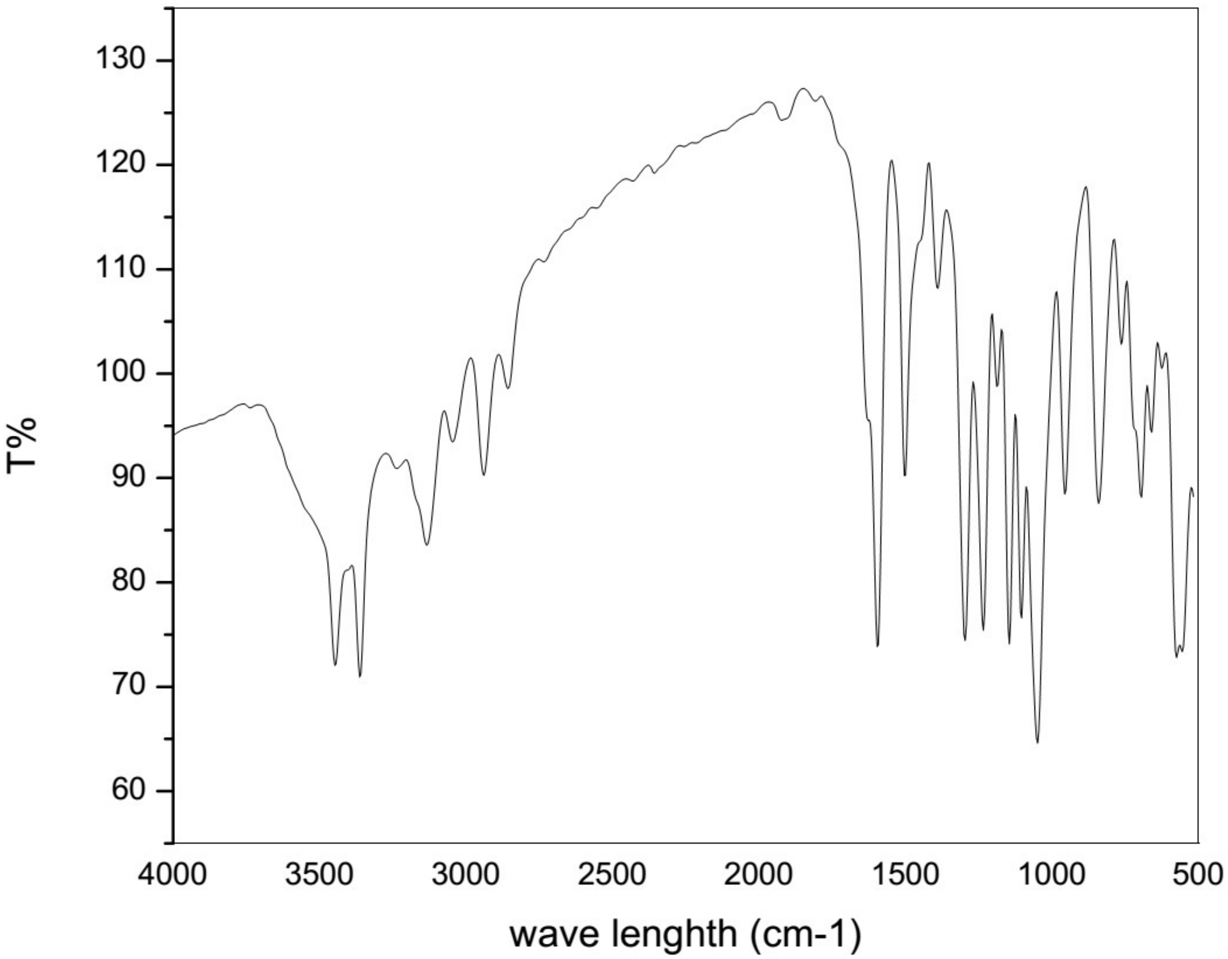

Figure : IR chart of compound 6a

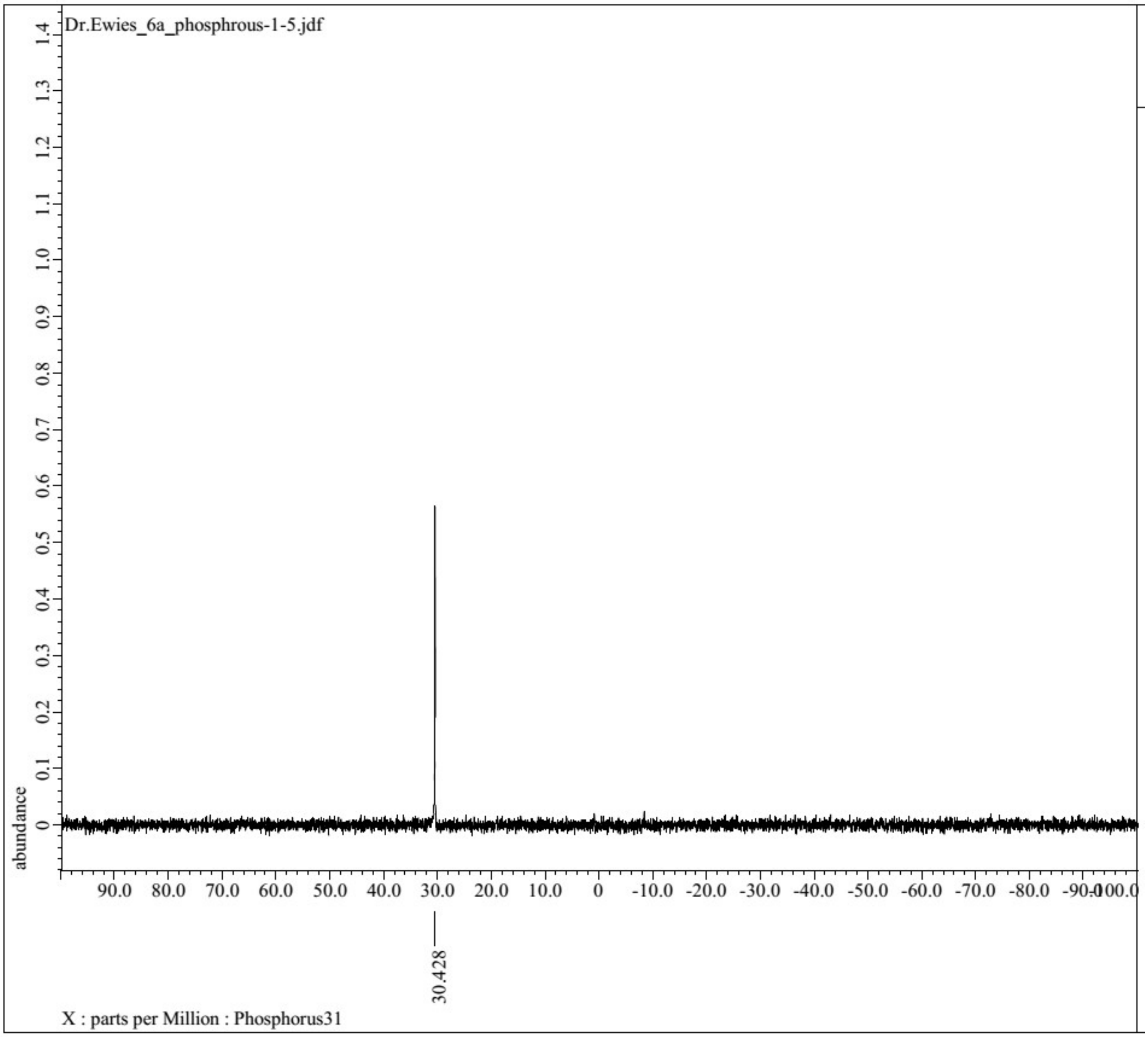

Figure : 31P NMR of compound 6a

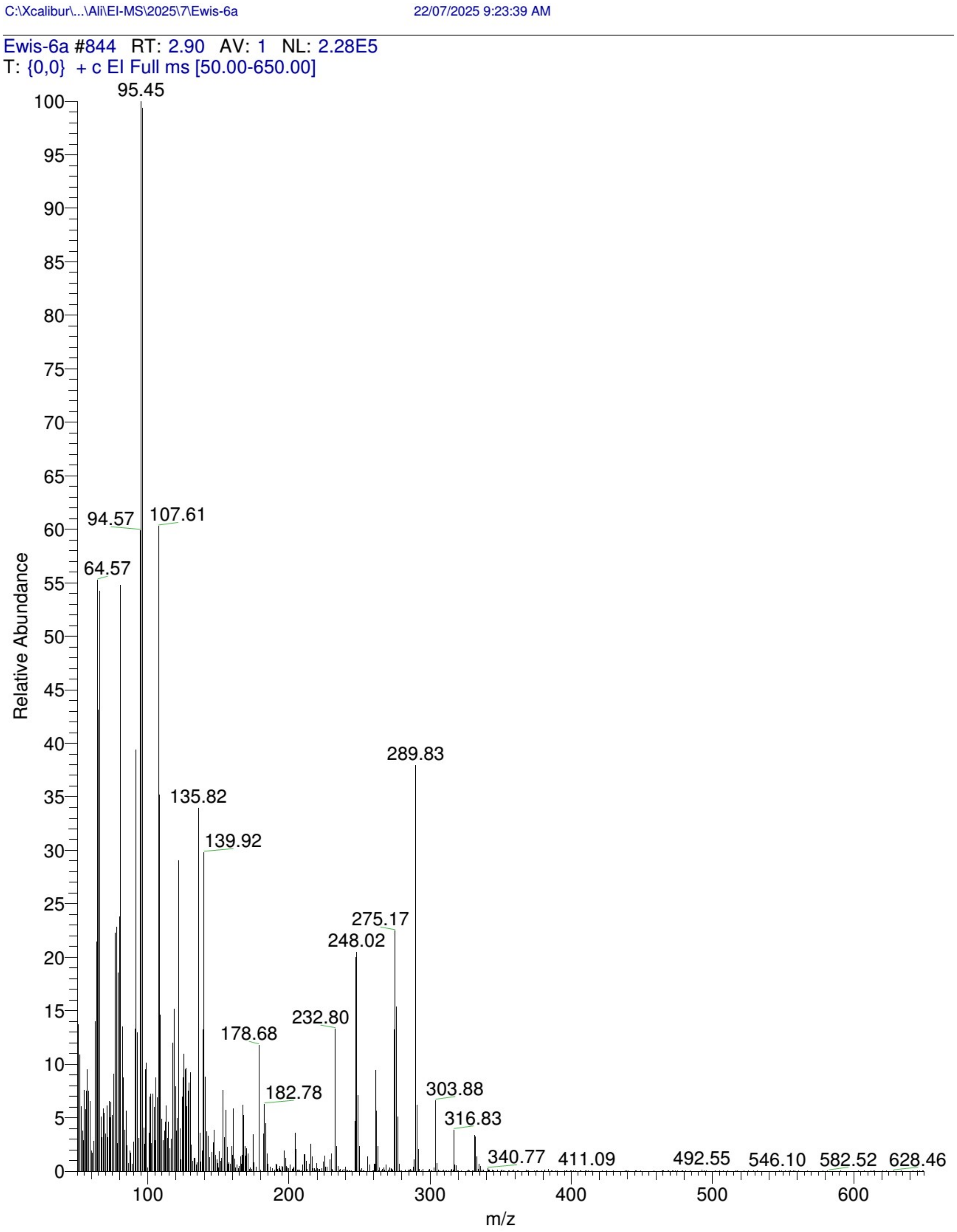

Figure : Mass spectrum of compound 6a

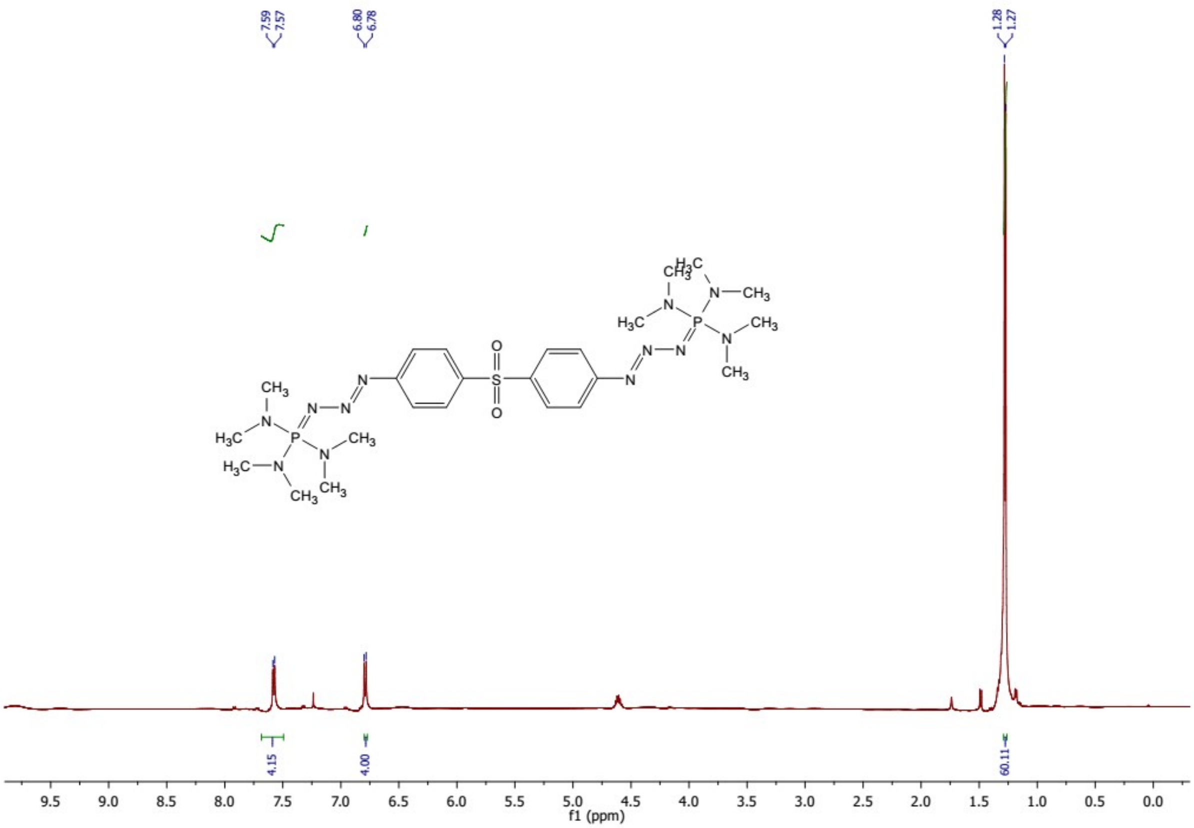

Figure : 1H NMR of compound 6b

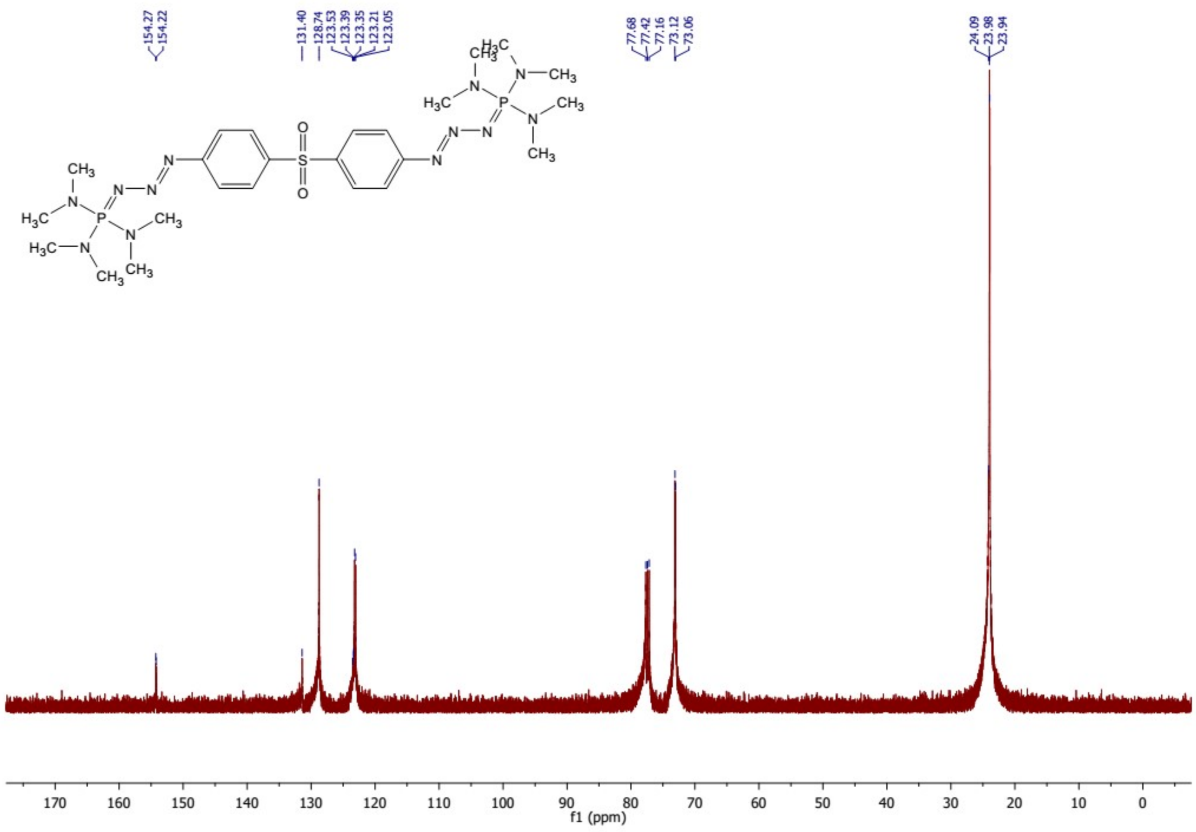

Figure : 13C NMR of compound 6b

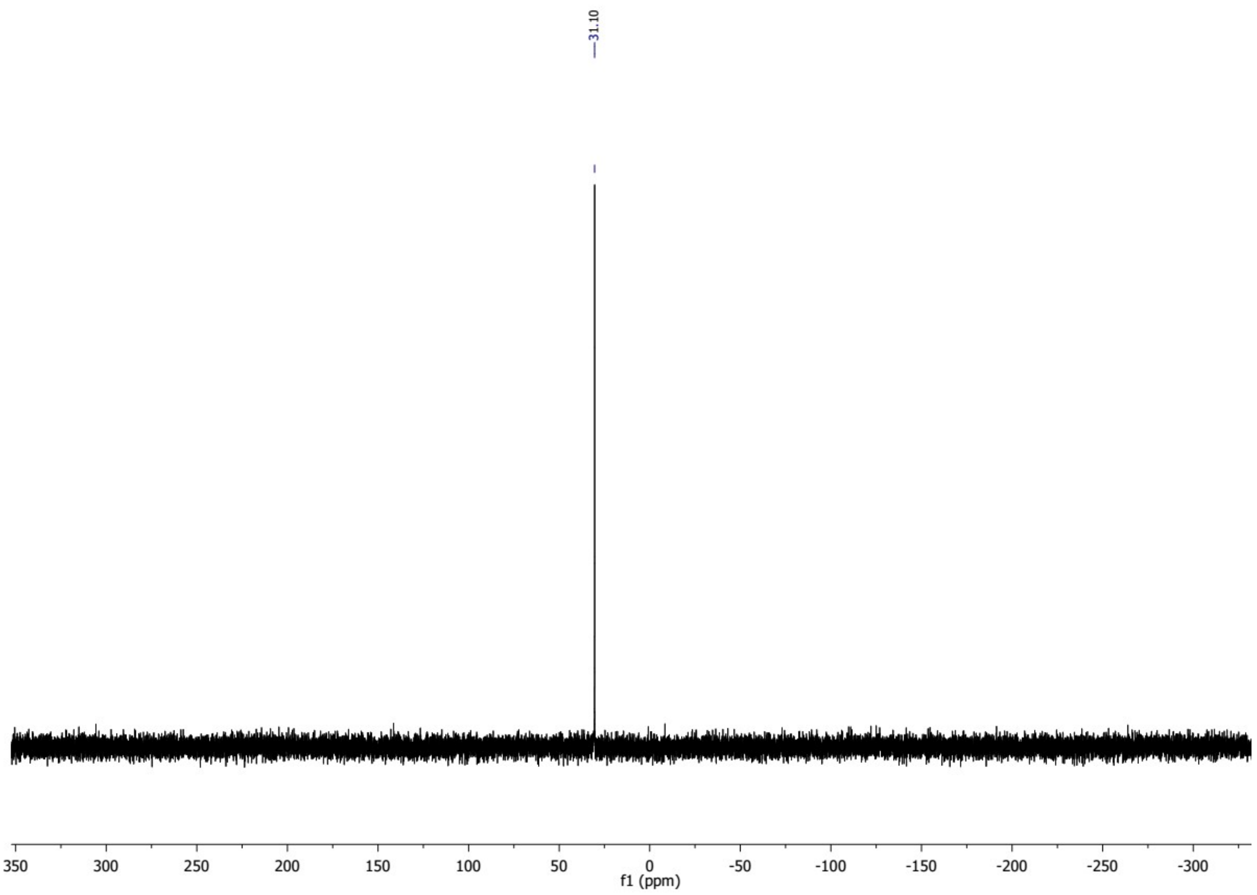

Figure : 31P NMR of compound 6b

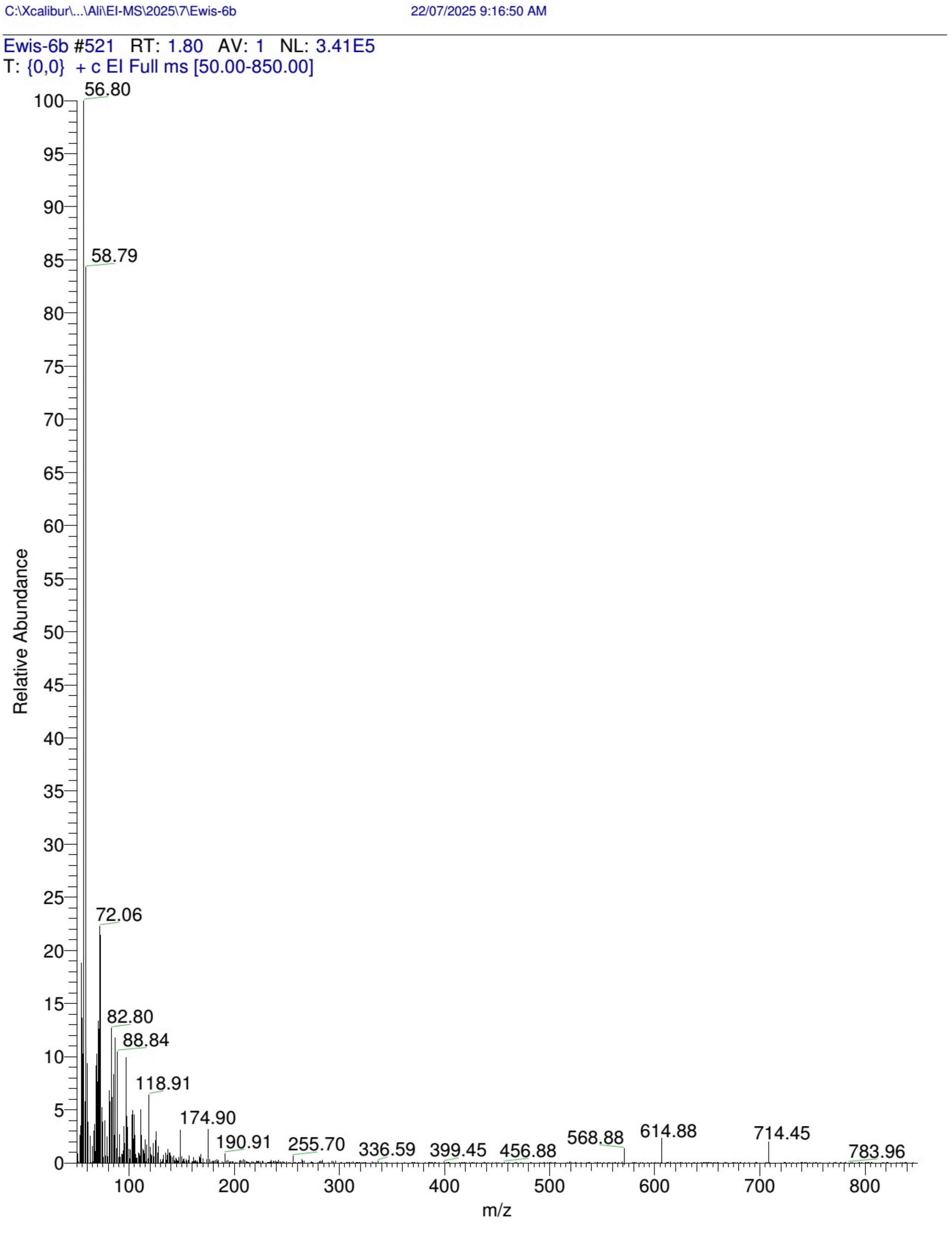

Figure : Mass spectrum of compound 6b

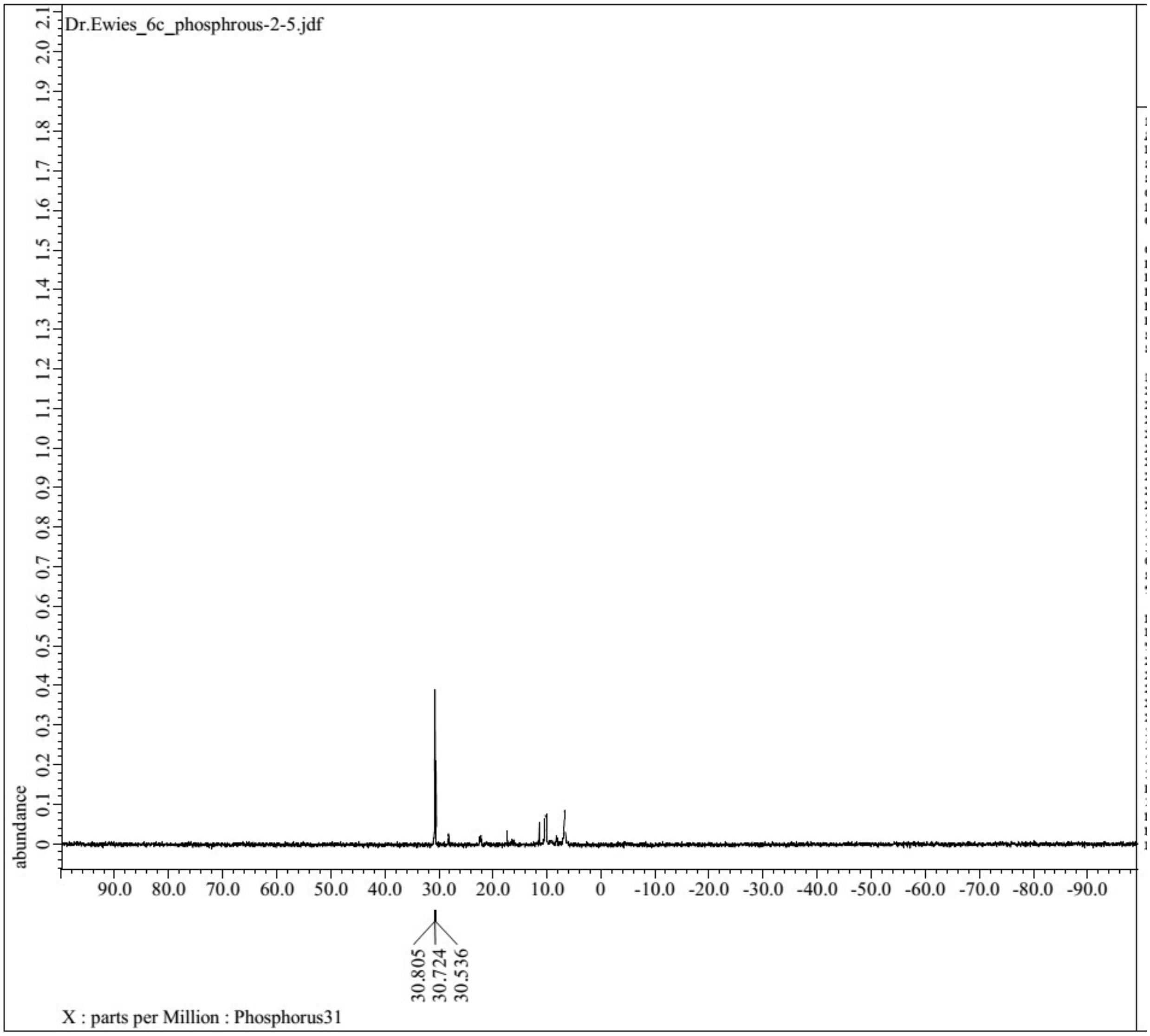

Figure : 31P NMR of compound 6c

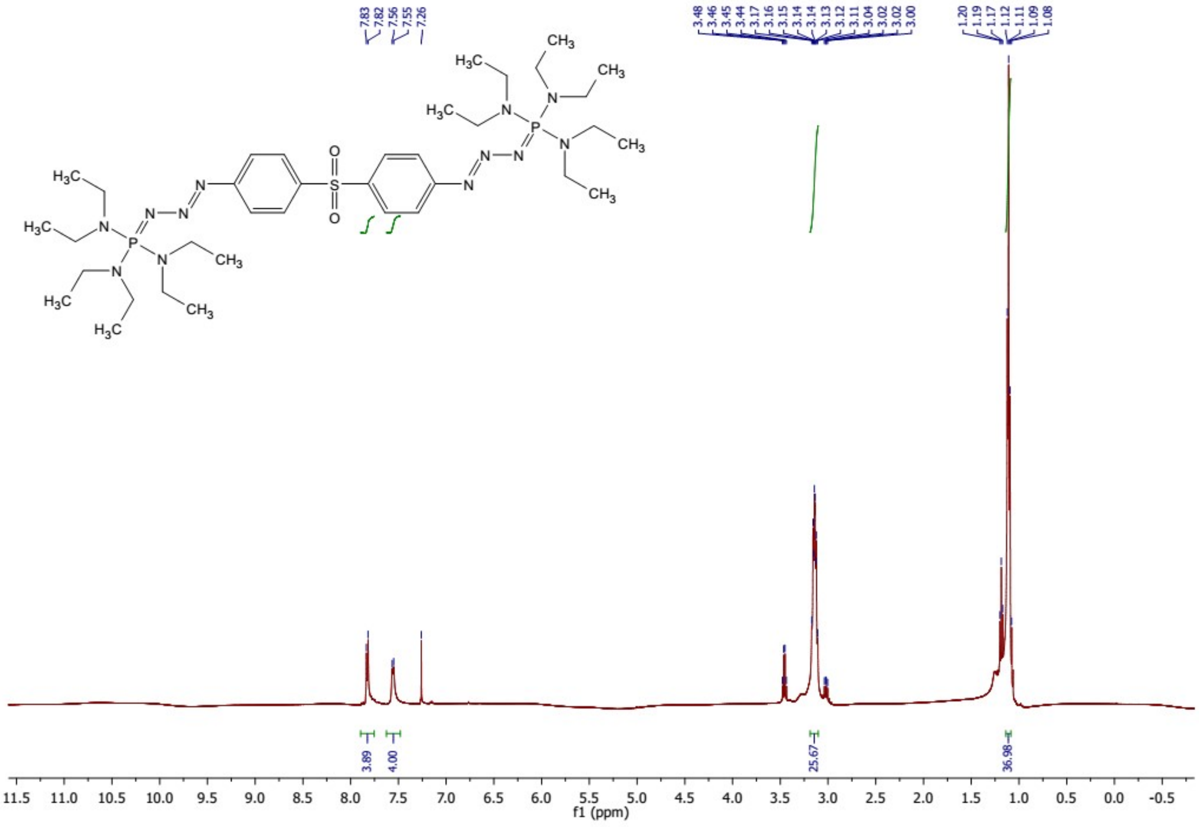

Figure : 1H NMR of compound 6d

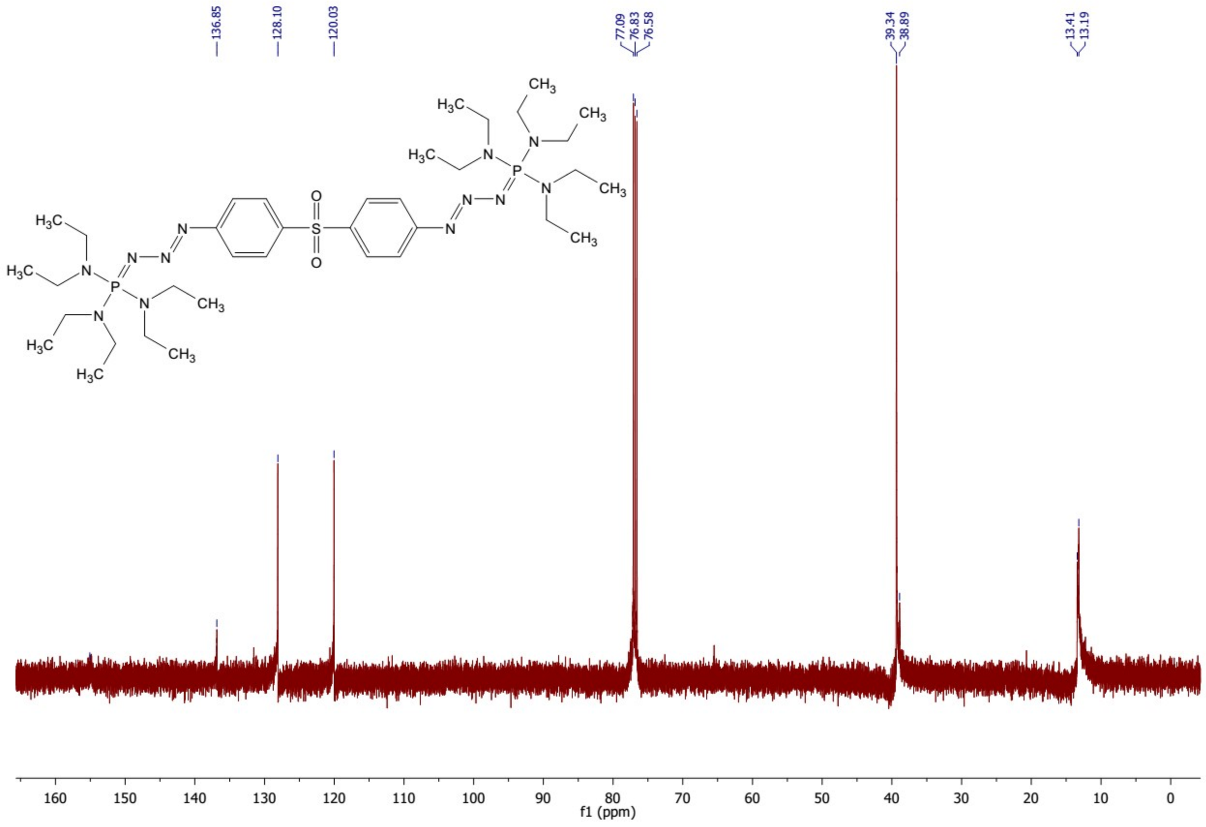

Figure : 13C NMR of compound 6d

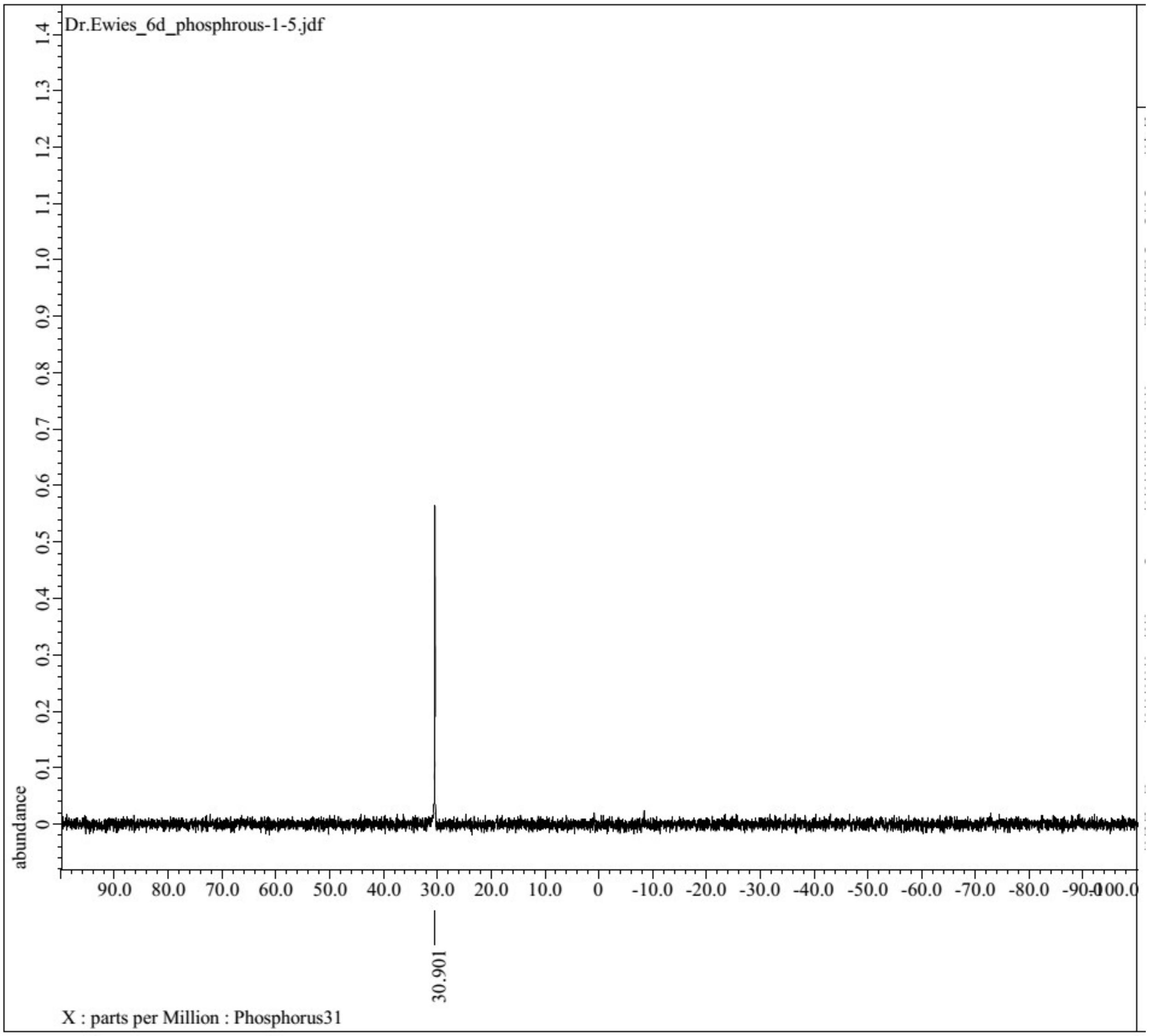

Figure : 31P NMR of compound 6d

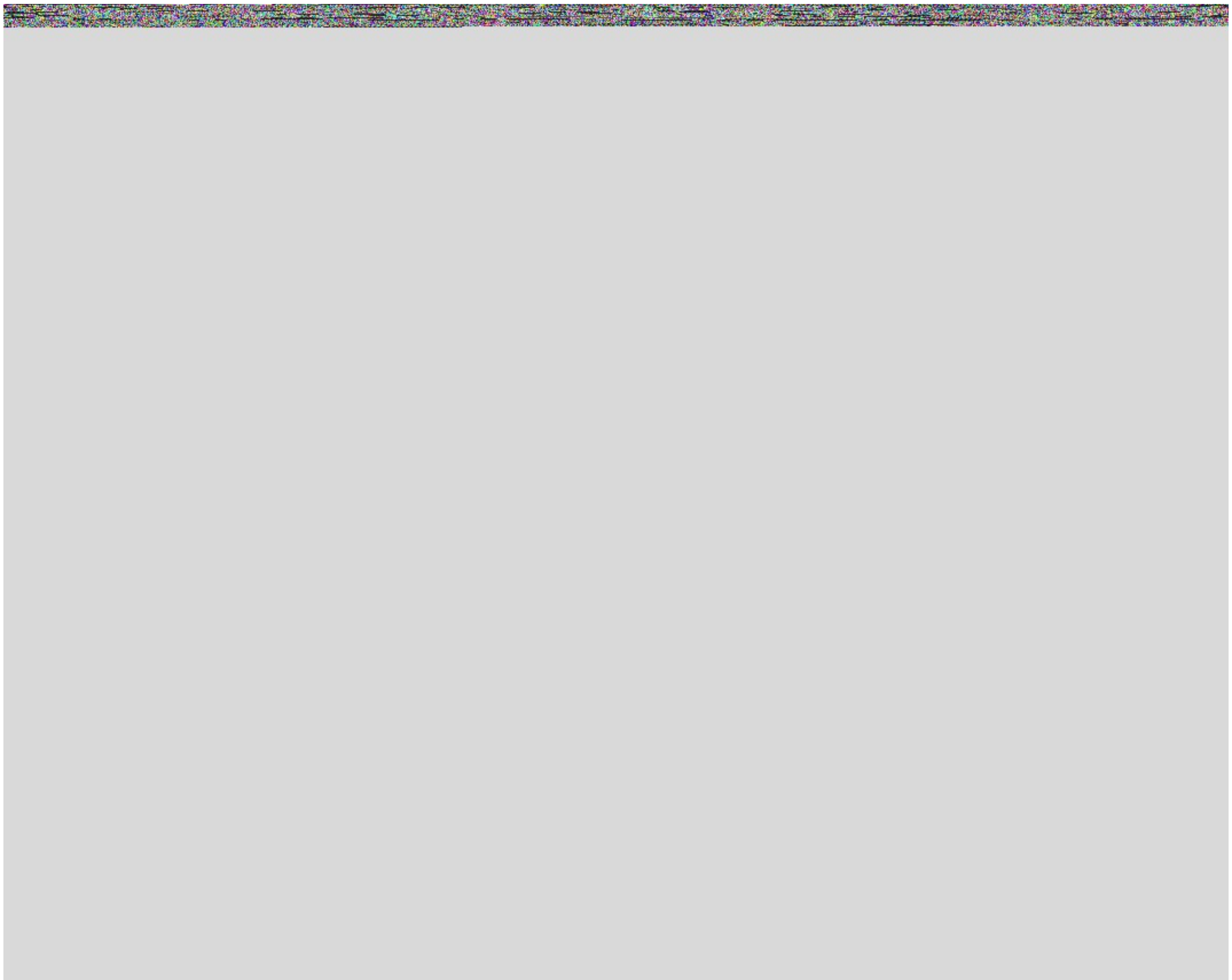

Figure : IR chart of compound 6d

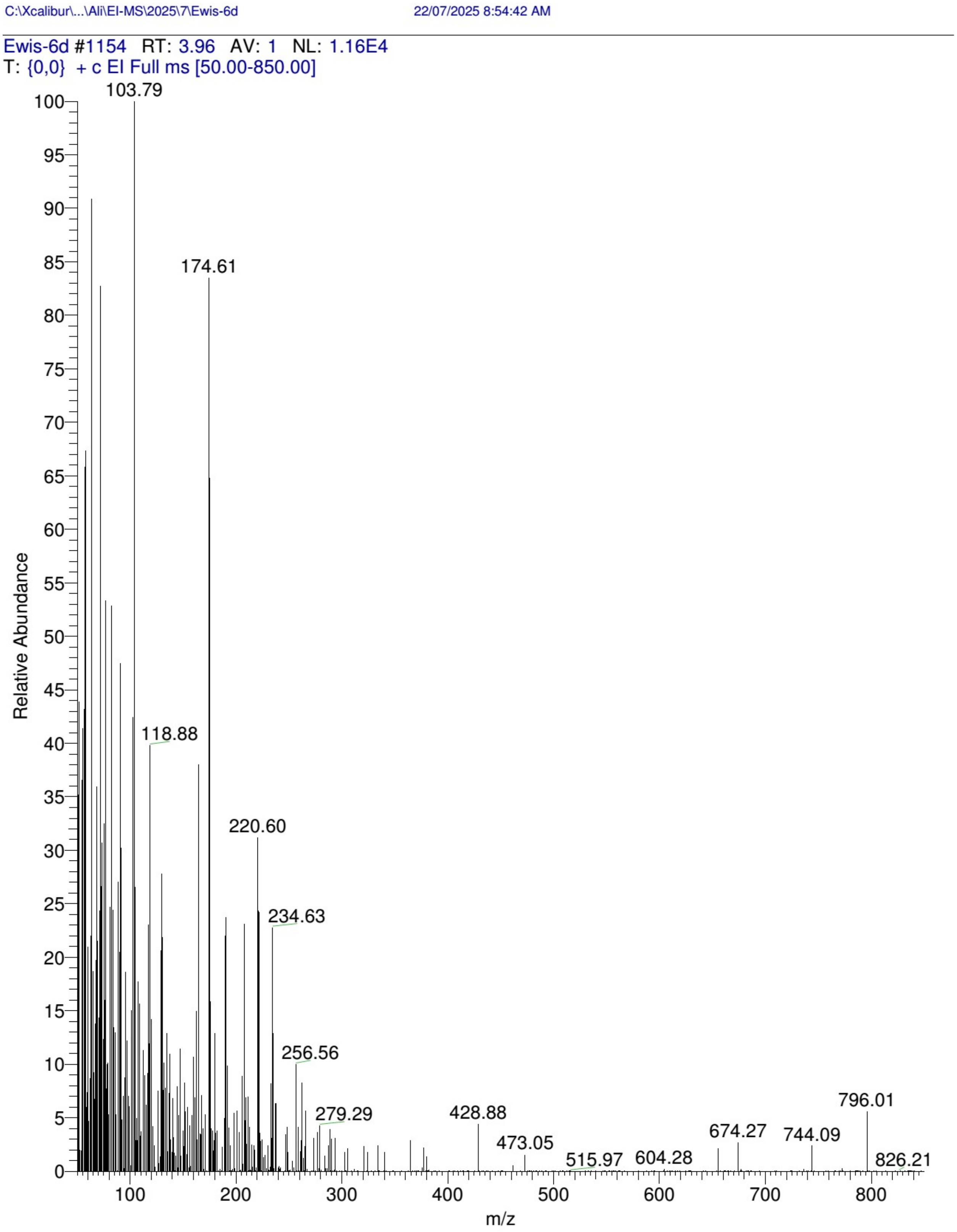

Figure : mass spectrum of compound 6d
